# Modern contraceptive use, unmet need, and demand satisfied among women of reproductive age who are married or in a union in the focus countries of the Family Planning 2020 initiative: a systematic analysis using the Family Planning Estimation Tool

**DOI:** 10.1016/S0140-6736(17)33104-5

**Published:** 2018-03-03

**Authors:** Niamh Cahill, Emily Sonneveldt, John Stover, Michelle Weinberger, Jessica Williamson, Chuchu Wei, Win Brown, Leontine Alkema

**Affiliations:** a The Department of Biostatistics and Epidemiology, University of Massachusetts Amherst, Amherst, MA, USA; b The School of Mathematics and Statistics, University College Dublin, Dublin, Ireland; c Avenir Health, Glastonbury, CN, USA; d Avenir Health, Washington, DC, USA; e The Bill & Melinda Gates Foundation, Seattle, WA, USA

## Abstract

**Background:**

The London Summit on Family Planning in 2012 inspired the Family Planning 2020 (FP2020) initiative and the 120×20 goal of having an additional 120 million women and adolescent girls become users of modern contraceptives in 69 of the world's poorest countries by the year 2020. Working towards achieving 120 × 20 is crucial for ultimately achieving the Sustainable Development Goals of universal access and satisfying demand for reproductive health. Thus, a performance assessment is required to determine countries' progress.

**Methods:**

An updated version of the Family Planning Estimation Tool (FPET) was used to construct estimates and projections of the modern contraceptive prevalence rate (mCPR), unmet need for, and demand satisfied with modern methods of contraception among women of reproductive age who are married or in a union in the focus countries of the FP2020 initiative. We assessed current levels of family planning indicators and changes between 2012 and 2017. A counterfactual analysis was used to assess if recent levels of mCPR exceeded pre-FP2020 expectations.

**Findings:**

In 2017, the mCPR among women of reproductive age who are married or in a union in the FP2020 focus countries was 45·7% (95% uncertainty interval [UI] 42·4–49·1), unmet need for modern methods was 21·6% (19·7–23·9), and the demand satisfied with modern methods was 67·9% (64·4–71·1). Between 2012 and 2017 the number of women of reproductive age who are married or in a union who use modern methods increased by 28·8 million (95% UI 5·8–52·5). At the regional level, Asia has seen the mCPR among women of reproductive age who are married or in a union grow from 51·0% (95% UI 48·5–53·4) to 51·8% (47·3–56·5) between 2012 and 2017, which is slow growth, particularly when compared with a change from 23·9% (22·9–25·0) to 28·5% (26·8–30·2) across Africa. At the country level, based on a counterfactual analysis, we found that 61% of the countries that have made a commitment to FP2020 exceeded pre-FP2020 expectations for modern contraceptive use. Country success stories include rapid increases in Kenya, Mozambique, Malawi, Lesotho, Sierra Leone, Liberia, and Chad relative to what was expected in 2012.

**Interpretation:**

Whereas the estimate of additional users up to 2017 for women of reproductive age who are married or in a union would suggest that the 120 × 20 goal for all women is overly ambitious, the aggregate outcomes mask the diversity in progress at the country level. We identified countries with accelerated progress, that provide inspiration and guidance on how to increase the use of family planning and inform future efforts, especially in countries where progress has been poor.

**Funding:**

The Bill & Melinda Gates Foundation, through grant support to the University of Massachusetts Amherst and Avenir Health.

## Introduction

The international community has agreed that the right to health includes the right to control one's health and body, including sexual and reproductive freedom.^1^ The Family Planning 2020 (FP2020) initiative is a global movement that supports this right and therefore the rights of women and girls to decide freely and for themselves whether, when, and how many children they want to have. The initiative is an outcome of the 2012 London Summit on Family Planning where more than 20 national governments made commitments to address the policy, financing, delivery, and sociocultural barriers to women accessing contraceptive information, services, and supplies, and donors pledged US$2·6 billion in funding.^2^ The overall goal of FP2020 is to reach 120 million additional users of modern contraceptive methods in the world's poorest countries by 2020 (the 120 × 20 goal).^3^ Working towards the goal is crucial to meeting Sustainable Development Goals (SDGs) related to health (Goal 3)^4^ and gender equality and women's empowerment (Goal 5).^4^

**Research in context**

**Evidence before this study**

Estimates of family planning indicators are produced annually by the UN Population Division and by the Track20 project. Both sets of estimates are based on the family planning estimation model (FPEM). Track20 supports national efforts to collect, analyse, and use existing data to track annual progress in family planning for the Family Planning 2020 (FP2020) initiative. The previous FP2020 publication included estimates up to the year 2016.

**Added value of this study**

In this study, we evaluate progress in family planning indicators in the focus countries of the FP2020 initiative between 2012 and 2017 using an updated database, including data obtained from Track20 annual monitoring and evaluation workshops, and an updated version of the FPEM. The updated FPEM is better equipped to capture recent changes (ie, changes that occur between the two most recent observations) in contraceptive prevalence and includes additional survey-specific information (uncertainty estimates and reference periods). Our study presents a counterfactual analysis to distinguish countries that have made the best or worst progress relative to pre-FP2020 expectations. The comparison of estimates for the current modern contraceptive prevalence rate (mCPR) with counterfactual projections reflecting expectations for mCPR at the beginning of FP2020, shows that, overall, more than half of the countries with data after 2012 exceeded pre-FP2020 expectations to some degree. Kenya and Mozambique are highlighted as making the most progress in terms of increasing modern contraceptive use.

**Implications of all the available evidence**

Family planning programmes need to focus on areas where progress is lacking. Countries, such as Kenya, that have made the most progress in terms of increasing mCPR and decreasing unmet need can provide case studies for achieving success in other countries.

Progress towards achieving the 120 × 20 goal is monitored by the Track20 project.^5^ Track20 engages at the country level helping to develop effective programme strategies and plans. In particular, the project implements annual monitoring and evaluation workshops, which support national efforts to collect, analyse, and use data to track family planning progress, relying mainly on modelling applications such as the Family Planning Estimation Tool^6^ (FPET). FPET is a web application that uses historical survey data and family planning service data to produce estimates and projections of family planning indicators over time, used currently for women who are married or in a union of reproductive age. FPET is a country-specific (local) implementation (appendix p 17) of the global family planning estimation model^7^ used by the UN Population Division.

In this study, we update the global family planning estimation model and subsequently the FPET tool and use it to evaluate the performance of 68 out of the 69 countries involved in the FP2020 initiative. Western Sahara, which has no data, was omitted from the analysis, as it was not included in the Track20 reporting for the initiative. Analysis includes the assessment of four core family planning indicators: the modern contraceptive prevalence rate (mCPR); the number of additional women married or in a union of reproductive age who are users of modern methods of contraception; the percentage of women with an unmet need for modern methods of contraception; and the percentage of women with their demand for contraception satisfied with a modern method.

## Methods

### Definitions and data sources

Modern methods of contraception include female and male sterilisation, oral hormonal pills, the intra-uterine device, male and female condoms, injectables, the implant (including Norplant; Wyeth-Ayerst, Collegeville, PA, USA), vaginal barrier methods, standard days method, lactational amenorrhoea method, and emergency contraception. Traditional methods of contraception include abstinence, the withdrawal method, the rhythm method, douching, and folk methods.

Contraceptive prevalence was measured as the percentage of women who report themselves or their partners as currently using at least one contraceptive method of any type (modern or traditional). Unmet need for family planning was defined as the percentage of women who want to stop or delay childbearing but who are not currently using any method of contraception to prevent pregnancy. Observations of unmet need for family planning in our database were, whenever possible, based on the revised algorithm of the indicator designed to improve comparability within and across countries.^8^ The estimates reported in this study are for women of reproductive age (15–49 years) who are currently married or in a union. With the exception of India, the FP2020 country data uses the UN Population Division database for contraceptive prevalence and unmet need for family planning^9^ as a base (appendix p 4). These data were obtained from nationally representative household surveys, the Demographic and Health Surveys (DHS), Performance Monitoring and Accountability 2020 (PMA2020) surveys, the Multiple Indicator Cluster Surveys, and the Reproductive Health Surveys. In India, state-level data for family planning indicators were obtained from multiple rounds of the DHS (also known as the National Family Health Survey [NFHS]), the District Level Household and Facility Survey, and the Annual Health Survey (appendix p 6). In some countries, survey data for modern contraceptive use are supplemented with family planning service data^10^ (service statistics; appendix pp 7, 18–21) that have been obtained through Track20 monitoring and evaluation workshops. The estimates presented in this report are based on 733 survey observations of contraceptive prevalence between 1969 and 2017 from 67 countries and 32 Indian states, and 462 survey observations of unmet need for family planning from 64 countries and 32 Indian states. Additionally, 85 service statistic observations for estimated modern contraceptive use are included in the database for 13 countries.

### The family planning estimation model

The family planning estimation model combines a Bayesian hierarchical model with country-specific time trends to yield estimates of contraceptive prevalence and unmet need for family planning for women of reproductive age who are married or in a union aged 15–49 years. The model accounts for differences by data source, sample population, and contraceptive methods included in the measure.^7^ For every country, the family planning estimation model models contraceptive prevalence with an expected trend that assumes contraceptive prevalence will begin with a gradual increase, the increase will subsequently become more rapid, and then it will begin to slow down when high levels of prevalence are reached. The parameters that control the trend are estimated hierarchically, such that estimates are based on the data available in the country of interest, and also the sub-regional, regional, and global experience. Distortions are added to capture how the level of the observed data deviates from the level of the expected trend, producing estimates of contraceptive prevalence with uncertainty. The family planning estimation model produces projections for contraceptive prevalence that are informed by the most recent levels of the indicator, as well as by the historical trends. In some cases the effect of the historical trend results in changes between recent observations having less impact on current estimates and projections. We have adjusted the family planning estimation model to correct for this, the details of which are described below. Estimates of unmet need were obtained by modelling the relationship between contraceptive prevalence and unmet need. Similar to the model for contraceptive prevalence, a hierarchical approach was used to estimate parameters and time-dependent distortions were added to capture country-specific changes in trends of unmet need.

The global family planning estimation model and subsequently the FPET tool were updated to address some limitations of the original model, specifically, to better capture changes in recently observed data and include survey sample-specific uncertainties, to ultimately provide more precise estimates, projections, and associated uncertainties. The updates can be summarised as follows. Time-dependent distortions added to the expected trend for total contraceptive prevalence now capture how rates of change in the observed data (ie, faster or slower rates of change in contraceptive prevalence) deviate from the rates of change indicated by the expected trend. Projections are informed by recent changes that have occurred in contraceptive prevalence (ie, the difference between the two most recent surveys) as well as past experience (appendix pp 9–13). For observations that belong to the DHS survey source, survey-specific sampling error variances were computed from the corresponding micro-data (appendix pp 14, 15). Survey-specific sampling error variances were imputed for the remaining observations (appendix p 15). For each survey source, sampling variances were included in the model in combination with an estimated non-sampling error to fully account for the uncertainty in the observed data (appendix pp 16, 17). Information relating to the start and end year of when a survey was done has been included in the model, therefore a survey observation is no longer assumed to only provide information for one calendar year (appendix p 15).

### Assessment of contraceptive use between 2012 and 2017

Estimates and projections of modern contraceptive prevalence, unmet need for, and demand satisfied with modern contraceptive methods were produced by the FPET for 68 FP2020 countries individually and for regional and overall aggregates.^11^ Demand satisfied was calculated based on the definition provided by Fabic and colleagues^12^ in 2015. In India, the FPET was applied at the state level and the state estimates were aggregated to provide national totals. Trends were analysed in terms of the level of the indicators and the changes that have been seen between 2012 and 2017. If we consider that all FP2020 countries have a demand for contraception, progress within individual countries and overall was deemed positive if we see an increase in mCPR and demand satisfied with modern methods.

### Contribution of women of reproductive age who are married or in a union to the 120×20 goal

The number of additional users is the difference in the total number of modern contraceptive users between two points in time. Following this, estimates for mCPR combined with population data for women of reproductive age who are married or in a union^13^, ^14^ (appendix p 8) were used to determine the changes in the number of women using modern methods of contraception between 2012 and 2017. This provided an estimate for the number of married or in a union users of reproductive age who have contributed to the FP2020 120 × 20 goal thus far.

### Comparison of expected versus observed levels of mCPR since 2012

We quantified the progress that has been made towards increasing modern contraceptive use since 2012 by analysing a reduced input dataset (leaving out all observations that had a start date greater than 2012). The reduced input data provided counterfactual model-based trends that reflect pre-FP2020 expectations for contraceptive use (appendix pp 21, 22). By comparing estimates and counterfactual projections in the years where data were removed, we assessed whether progress in the FP2020 countries has been faster or slower than expected. We calculated attainment probabilities that provided a continuous measure of how current estimates for post-2012 observations compare with 2012 expectations. The attainment probabilities directly measure the likelihood of observing an outcome that is equal to or greater than the estimate based on the full dataset (eg, a 10% attainment probability means that there was a 10% chance of attaining the level observed based on pre-2012 expectations). This metric is directly comparable across all countries (the interpretation is the same across countries and does not need to be combined with an uncertainty interval) and the combined set of probabilities provides an informative and concise summary of progress as compared with what was expected for all countries. When assessing country-specific trends, modern contraceptive use was deemed to have had made positive progress since the beginning of the FP2020 initiative in 2012 if two criteria were met: there was an increase in observed levels of mCPR compared with expected levels of mCPR; and expected trends indicated that the increase in mCPR had a low attainment probability (<50%).

### Role of the funding source

The funder of the study had no role in study design, data collection, data analysis, data interpretation, or writing of the report. All authors had full access to all data in the study and had final responsibility for the decision to submit for publication.

## Results

**Figure 1:**
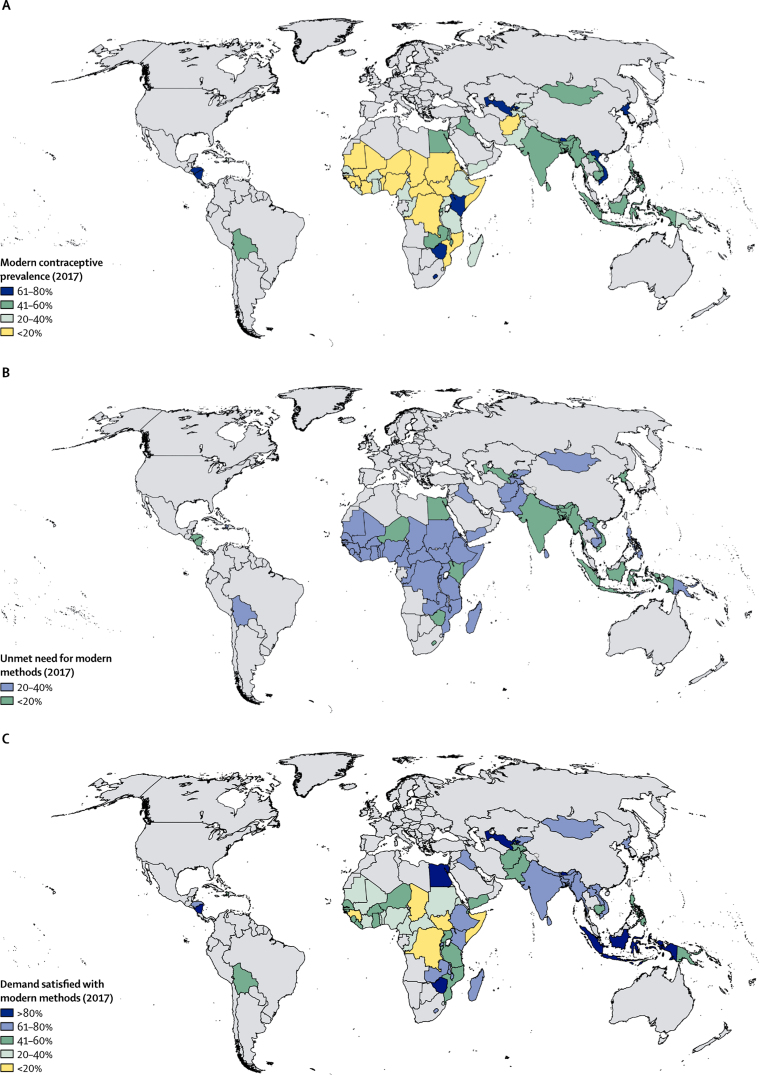
2017 estimates for family planning indicators Median estimates of (A) modern contraceptive prevalence, (B) unmet need for, and (C) demand satisfied with modern methods in 2017 for 68 Family Planning 2020 countries.

Modelled estimates and projections for all FP2020 countries, regional aggregates, and an all-country aggregate are shown in the appendix (pp 25–112) for the three indicators, mCPR, unmet need for, and demand satisfied with modern contraceptive methods. An overview of the current median levels of these three indicators at the country level is shown in figure 1. In 2017, mCPR ranges from 2·6% (95% uncertainty interval [UI] 0·7–7·7) in Somalia to 77·4% (67·3–85·0) in Nicaragua (table 1). As well as having the highest levels of mCPR in 2017, Nicaragua had the best performance in terms of other indicators (unmet need for modern methods was 10·2% [95% UI 6·0–16·6] and the demand satisfied with modern methods was 88·4% [80·4–93·4]). By contrast, the Democratic Republic of the Congo had the highest unmet need for modern methods in 2017 of 39·9% (95% UI 32·1–48·4) and Somalia had the lowest demand satisfied of 7·8% (2·3–20·1).

**Table 1:** Modern contraceptive prevalence rate (mCPR), unmet need for modern methods, and demand satisfied with modern methods in 2017 for 68 Family Planning 2020 countries

	**mCPR (%)**	**Unmet need for modern methods (%)**	**Demand satisfied with modern methods (%)**
Afghanistan	20·9% (16·0–27·1)	27·9% (22·9–33·4)	42·7% (34·7–51·7)
Bangladesh	56·4% (46·1–65·7)	19·2% (13·7–26·3)	74·5% (64·3–82·5)
Benin	13·0% (8·0–19·6)	36·1% (28·6–44·1)	26·5% (17·4–36·8)
Bhutan	64·6% (48·1–78·9)	12·7% (6·0–23·2)	83·4% (68·0–92·8)
Bolivia	44·1% (26·7–60·4)	36·7% (23·2–53·5)	54·5% (33·5–71·9)
Burkina Faso	24·7% (21·1–28·8)	27·2% (21·6–33·6)	47·6% (40·9–55·1)
Burundi	25·2% (20·4–30·7)	33·1% (28·6–37·8)	43·2% (36·4–50·5)
Cambodia	42·8% (31·8–53·4)	28·8% (21·0–39·9)	59·8% (45·4–71·2)
Cameroon	21·8% (13·2–29·7)	33·2% (25·6–42·1)	39·6% (26·0–50·8)
Central African Republic	14·5% (6·8–27·4)	30·0% (20·6–41·5)	32·4% (17·8–51·1)
Chad	4·3% (2·7– 6·8)	22·7% (18·0–28·2)	16·0% (10·4–23·6)
Comoros	18·7% (11·1–29·4)	35·9% (28·6–44·0)	34·1% (22·1–48·0)
Congo (Brazzaville)	23·9% (14·5–35·6)	37·9% (28·3–49·1)	38·5% (24·3–54·0)
Côte d'Ivoire	15·8% (12·1–20·0)	29·0% (22·4–36·6)	35·3% (27·6–43·6)
Democratic Republic of the Congo	10·0% (5·8–16·2)	39·9% (32·1–48·4)	20·0% (12·1–30·3)
Djibouti	24·3% (15·1–36·1)	30·0% (19·6–42·1)	44·7% (30·4–61·1)
Egypt	59·7% (49·3–69·5)	12·9% (8·4–18·3)	82·2% (73·3–89·1)
Eritrea	9·7% (5·2–18·2)	30·4% (21·7–40·6)	24·4% (13·8–38·8)
Ethiopia	37·5% (31·6–43·6)	23·7% (19·9–27·9)	61·2% (54·3–67·8)
Gambia	9·8% (6·0–15·3)	26·7% (20·6–33·9)	26·8% (17·6–38·2)
Ghana	27·1% (22·0–32·9)	34·0% (28·7–39·7)	44·3% (37·3–52·0)
Guinea	6·2% (3·4–10·8)	25·6% (19·5–32·8)	19·5% (11·5–30·4)
Guinea-Bissau	16·3% (9·2–26·6)	23·2% (15·6–32·6)	41·2% (26·4–57·4)
Haiti	35·4% (24·2–48·4)	35·5% (27·0–43·9)	49·8% (36·6–63·6)
Honduras	65·4% (52·8–75·8)	18·6% (11·6–28·4)	77·8% (65·4–86·6)
India	52·8% (44·8–60·7)	18·9% (14·7–24·3)	73·7% (65·8–80·0)
Indonesia	59·4% (51·6–67·0)	13·7% (9·7–18·7)	81·2% (74·1–87·1)
Iraq	43·6% (27·9–59·1)	27·4% (16·8–41·0)	61·2% (41·6–77·3)
Kenya	62·3% (54·0–69·6)	16·8% (12·4–22·3)	78·8% (71·2–84·7)
Kyrgyzstan	39·1% (27·9–51·3)	20·3% (14·8–26·5)	65·7% (53·2–76·7)
Laos	49·4% (35·7–62·2)	23·2% (15·6–32·6)	67·9% (53·3–79·8)
Lesotho	60·9% (50·6–70·4)	17·8% (12·1–24·7)	77·4% (67·6–85·1)
Liberia	23·6% (16·0–30·1)	31·5% (25·5–38·1)	42·7% (32·2–51·6)
Madagascar	39·5% (27·4–52·8)	24·9% (17·4–33·5)	61·3% (46·9–74·2)
Malawi	58·5% (50·6–66·1)	19·2% (14·6–24·3)	75·3% (67·9–81·8)
Mali	13·5% (8·7–20·1)	26·5% (20·8–33·1)	33·7% (23·6–44·9)
Mauritania	16·0% (9·9–24·4)	32·6% (24·4–41·8)	32·9% (22·0–46·0)
Mongolia	53·2% (39·8–65·6)	20·0% (12·7–29·7)	72·7% (58·5–83·3)
Mozambique	31·4% (19·7–38·1)	24·9% (20·2–30·6)	55·7% (41·3–63·9)
Myanmar	52·2% (44·9–59·2)	17·0% (13·2–21·3)	75·4% (68·4–81·4)
Nepal	50·8% (39·6–61·9)	25·9% (18·2–34·3)	66·2% (54·2–77·1)
Nicaragua	77·4% (67·3–85·0)	10·2% (6·0–16·6)	88·4% (80·4–93·4)
Niger	14·7% (10·9–19·2)	20·9% (16·2–26·6)	41·3% (32·9–50·1)
Nigeria	13·1% (9·8–17·3)	25·3% (20·6–30·7)	34·1% (26·6–42·4)
North Korea	64·3% (48·7–77·6)	16·5% (8·9–27·6)	79·6% (64·6–89·6)
Pakistan	30·6% (24·4–38·0)	29·7% (23·3–37·3)	50·8% (41·6–60·1)
Palestine	47·4% (32·8–61·6)	25·0% (15·7–37·5)	65·5% (48·1–79·2)
Papua New Guinea	28·7% (14·8–46·4)	32·1% (21·6–44·6)	46·9% (28·0–66·2)
Philippines	41·4% (30·0–53·4)	31·9% (23·5–42·1)	56·4% (42·4–69·1)
Rwanda	50·2% (41·7–58·5)	23·3% (17·9–29·3)	68·3% (59·3–76·2)
São Tomé and Príncipe	46·8% (33·9–60·0)	31·2% (21·5–41·2)	59·9% (45·6–73·4)
Senegal	21·7% (15·6–29·2)	26·9% (22·5–31·6)	44·6% (35·5–54·3)
Sierra Leone	19·5% (13·1–26·2)	27·1% (21·3–33·4)	41·8% (31·0–51·8)
Solomon Islands	31·2% (17·1–48·4)	27·4% (17·9–38·9)	52·7% (34·3–71·1)
Somalia	2·6% (0·7– 7·7)	31·4% (19·8–45·5)	7·8% (2·3–20·1)
South Sudan	3·6% (1·7– 8·0)	30·6% (19·9–43·2)	10·7% (4·9–22·0)
Sri Lanka	58·5% (38·5–74·7)	21·7% (11·1–39·3)	72·8% (50·5–86·8)
Sudan	13·0% (7·6–21·2)	29·4% (21·2–39·4)	30·6% (19·3–44·5)
Tajikistan	30·0% (19·6–42·8)	24·9% (18·7–32·0)	54·4% (40·8–67·8)
Timor-Leste	25·2% (20·1–31·1)	27·4% (22·9–32·1)	47·9% (40·2–55·7)
Togo	20·9% (14·8–26·2)	35·3% (29·3–41·7)	37·2% (28·3–45·1)
Uganda	34·3% (28·8–40·1)	33·2% (28·7–38·0)	50·7% (44·1–57·6)
Tanzania	34·9% (27·4–42·3)	27·8% (22·8–33·5)	55·6% (46·2–64·1)
Uzbekistan	65·7% (47·0–80·8)	12·6% (5·9–22·9)	83·8% (68·1–93·0)
Vietnam	66·3% (55·6–75·5)	17·4% (11·0–26·6)	79·2% (68·0–87·1)
Yemen	33·6% (23·8–45·4)	31·8% (24·5–39·3)	51·3% (39·2–64·0)
Zambia	48·2% (37·2–59·7)	23·6% (17·0–30·8)	67·1% (55·5–77·5)
Zimbabwe	66·3% (56·1–73·0)	11·9% (8·0–18·0)	84·7% (76·2–89·9)

Data in parentheses are 95% uncertainty intervals.

**Figure 2:**
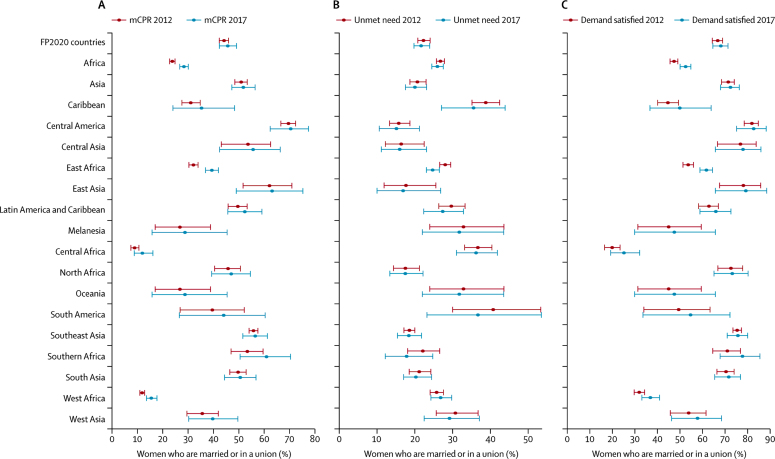
Aggregate estimates for 2012–17 of family planning indicators Percentage of women of reproductive age who are married or in a union (A) who use (mCPR), (B) have an unmet need for, or (C) have demand satisfied with, modern methods of contraception. Estimates and 95% uncertainty intervals are shown for all Family Planning 2020 countries combined and for the country subregions. mCPR=modern contraceptive prevalence.

Figure 2 and the appendix (p 24) provides aggregate estimates of mCPR, unmet need for, and demand satisfied with modern methods in 2012 and 2017. Overall, in the FP2020 countries in 2017, mCPR was 45·7% (95% UI 42·4–49·1) and demand satisfied with modern methods was 67·9% (64·4–71·1). Unmet need for modern methods was estimated to be 21·6% (95% UI 19·7–23·9) in the same year. At the regional level, Asia, which includes the most highly populated countries in the initiative, has seen mCPR among women of reproductive age who are married or in a union grow from 51·0% (95% UI 48·5–53·4) to 51·8% (47·3–56·5) between 2012 and 2017. This is relatively slow growth compared with a change from 23·9% (95% UI 22·9–25·0) to 28·5% (26·8–30·2) across all of Africa. Within the African sub-regions that have more than one FP2020 country, the fastest growth occurred in east Africa with an increase in mCPR from 32·2% (95% UI 30·5–34·0) to 39·5% (37·0–42·0) between 2012 and 2017. In terms of additional users of modern methods, in 2017, women of reproductive age who are married or in a union in FP2020 countries have contributed 28·8 million (95% UI 5·8–52·5) to the 120 × 20 target.

**Figure 3:**
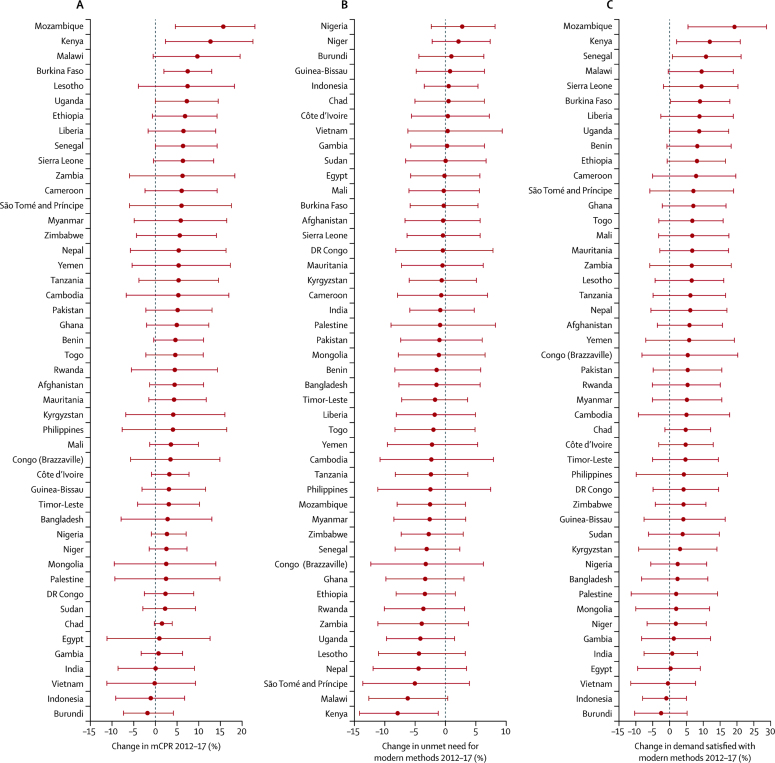
Changes in family planning indicators between 2012 and 2017 Estimated changes in (A) modern contraceptive prevalence (mCPR), (B) unmet need for, and (C) demand satisfied with modern methods between 2012 and 2017 for 47 Family Planning 2020 countries that have data after 2012. Horizontal lines are the 95% uncertainty intervals. Countries are ordered by decreasing point estimate. The red line indicates 0 percentage point change.

Of the 47 countries that have data after 2012, based on median estimates, 44 countries had an increase in mCPR and demand satisfied in the period between 2012 and 2017 (table 2). Mozambique and Kenya had the largest positive changes, with an increase in mCPR of 15·7% (95% UI 4·6–23·0) in Mozambique and 12·7% (2·3–22·5) in Kenya over the 5 years (figure 3). In absolute numbers, the increase in mCPR in Kenya translates to an additional 1·26 million (95% UI 0·58–1·89) women of reproductive age who are married or in a union using modern methods of contraception (table 2). Conversely, mCPR and demand satisfied have declined in Vietnam, Indonesia, and Burundi in the 5 years since the introduction of FP2020, with the largest decrease in these indicators occurring in Burundi (mCPR, 1·8 percentage points [95% UI −4·2 to 7·3]; demand satisfied, 2·5 percentage points [–5·2 to 10·3]). The 2012 national survey appears to be a high outlier relative to other datapoints in Burundi. Without this survey included, Burundi had an increase in mCPR of 4·5 percentage points (95% UI −1·8 to 11·1) between 2012 and 2017. In terms of unmet need, 37 countries had a decrease between 2012 and 2017 with Kenya making the most progress and decreasing unmet need by 7·8 percentage points (95% UI 1·1 to 14·1) in 5 years, followed by Malawi with a drop of 6·1 percentage points (−0·4 to 12·6) over the same period. Of the countries that have seen an increase in unmet need, Nigeria had the largest increase of 2·8 percentage points (−2·3 to 8·2).

**Table 2:** Changes between 2012 and 2017 in the modern contraceptive prevalence rate (mCPR), unmet need for modern methods, demand satisfied with modern methods, and the number of women of reproductive age who are married or in a union using modern methods of contraception

	**Change in mCPR (percentage points)**	**Change in unmet need for modern methods (percentage points)**	**Change in demand satisfied with modern methods (percentage points)**	**Change in the number of women of reproductive age who are married or in a union using modern contraception (millions)**
Afghanistan*	4·5 (−1·3 to 11·1)	−0·3 (−6·6 to 5·8)	5·9 (−3·6 to 15·7)	0·37 (0·08 to 0·71)
Bangladesh*	2·8 (−7·9 to 13·1)	−1·4 (−7·6 to 5·8)	2·4 (−8·2 to 11·3)	2·11 (−1·76 to 5·82)
Benin*	4·6 (−0·4 to 11·1)	−1·4 (−8·3 to 5·8)	8·2 (−0·7 to 18·2)	0·10 (0·01 to 0·21)
Bhutan	3·7 (−8·2 to 14·6)	−1·6 (−7·4 to 5·0)	2·6 (−7·4 to 11·0)	0·02 (0·00 to 0·03)
Bolivia	4·3 (−8·6 to 17·3)	−3·9 (−15·2 to 7·8)	5·1 (−9·8 to 19·3)	0·11 (−0·08 to 0·30)
Burkina Faso*	7·5 (2·0 to 13·0)	−0·2 (−5·7 to 5·4)	9·0 (0·3 to 17·8)	0·32 (0·15 to 0·49)
Burundi*†	−1·8 (−7·3 to 4·2)	1·0 (−4·3 to 6·4)	−2·5 (−10·3 to 5·2)	0·04 (−0·05 to 0·13)
Cambodia*	5·3 (−6·7 to 17·0)	−2·3 (−10·7 to 7·9)	5·0 (−9·2 to 17·8)	0·22 (−0·10 to 0·54)
Cameroon*	6·1 (−2·4 to 14·3)	−0·6 (−7·8 to 7·0)	7·9 (−5·0 to 19·6)	0·29 (−0·02 to 0·59)
Central African Republic	3·0 (−2·9 to 12·5)	−0·2 (−6·9 to 6·9)	5·0 (−6·0 to 18·1)	0·04 (−0·02 to 0·14)
Chad*	1·5 (−0·2 to 3·9)	0·6 (−5·0 to 6·4)	4·8 (−1·3 to 12·2)	0·05 (0·01 to 0·11)
Comoros	3·9 (−3·2 to 14·0)	−1·1 (−7·8 to 6·1)	5·6 (−5·4 to 18·5)	0·01 (0·00 to 0·02)
Congo (Brazzaville)*	3·5 (−5·7 to 14·9)	−3·2 (−12·2 to 6·3)	5·4 (−8·1 to 20·2)	0·04 (−0·02 to 0·11)
Côte d'Ivoire*	3·2 (−0·9 to 7·8)	0·4 (−5·5 to 7·3)	4·8 (−3·2 to 12·9)	0·10 (−0·04 to 0·26)
Democratic Republic of the Congo*	2·3 (−2·5 to 8·9)	−0·4 (−8·1 to 7·9)	4·2 (−4·8 to 14·5)	0·43 (−0·19 to 1·27)
Djibouti	5·1 (−3·5 to 16·2)	−1·2 (−8·4 to 5·6)	6·5 (−6·1 to 19·6)	0·01 (0·00 to 0·02)
Egypt*	0·9 (−11·2 to 12·6)	−0·1 (−5·7 to 5·7)	0·4 (−9·4 to 9·1)	1·03 (−0·96 to 2·95)
Eritrea	2·0 (−1·8 to 8·7)	0·3 (−6·3 to 7·3)	3·8 (−4·9 to 14·8)	0·02 (−0·01 to 0·08)
Ethiopia*	6·9 (−0·7 to 14·3)	−3·3 (−8·1 to 1·7)	8·1 (−0·7 to 16·6)	1·66 (0·58 to 2·76)
Gambia*	0·7 (−3·3 to 6·3)	0·3 (−5·7 to 6·5)	1·3 (−8·2 to 12·1)	0·01 (−0·01 to 0·02)
Ghana*	5·0 (−2·0 to 12·4)	−3·3 (−9·7 to 3·1)	7·1 (−2·1 to 16·7)	0·28 (0·00 to 0·58)
Guinea	1·3 (−1·4 to 5·7)	0·8 (−4·9 to 7·4)	3·1 (−4·4 to 13·0)	0·04 (−0·02 to 0·13)
Guinea-Bissau*	3·1 (−3·1 to 11·6)	0·8 (−4·8 to 6·5)	4·1 (−7·5 to 16·5)	0·01 (0·00 to 0·03)
Haiti	4·3 (−6·4 to 16·4)	−3·2 (−11·1 to 4·6)	5·1 (−7·1 to 18·1)	0·13 (−0·06 to 0·34)
Honduras	1·5 (−10·0 to 11·3)	−1·2 (−7·8 to 7·6)	1·5 (−9·7 to 9·8)	0·11 (−0·05 to 0·23)
India*	0·1 (−8·6 to 9·0)	−0·8 (−5·8 to 4·8)	0·8 (−7·5 to 8·3)	6·39 (−15·53 to 29·01)
Indonesia*	−1·1 (−9·1 to 6·8)	0·6 (−3·4 to 5·4)	−0·9 (−7·9 to 5·0)	0·43 (−3·65 to 4·38)
Iraq	3·4 (−8·8 to 15·6)	−1·2 (−9·8 to 8·5)	2·9 (−11·6 to 15·5)	0·49 (−0·22 to 1·17)
Kenya*	12·7 (2·3 to 22·5)	−7·8 (−14·1 to −1·1)	11·9 (2·1 to 20·9)	1·26 (0·58 to 1·89)
Kyrgyzstan*	4·1 (−6·8 to 16·1)	−0·6 (−5·9 to 5·1)	3·1 (−9·1 to 14·0)	0·06 (−0·06 to 0·18)
Laos	5·4 (−6·7 to 17·0)	−2·7 (−9·6 to 5·3)	5·0 (−7·6 to 15·5)	0·15 (−0·02 to 0·31)
Lesotho*	7·4 (−3·9 to 18·3)	−4·3 (−11·0 to 3·3)	6·6 (−4·2 to 16·1)	0·03 (0·00 to 0·05)
Liberia*	6·5 (−1·7 to 13·9)	−1·7 (−8·0 to 5·0)	8·9 (−2·6 to 18·9)	0·05 (0·00 to 0·10)
Madagascar	6·2 (−5·1 to 18·5)	−2·3 (−9·2 to 5·1)	6·2 (−7·0 to 18·5)	0·45 (−0·03 to 0·98)
Malawi*	9·7 (−0·5 to 19·6)	−6·1 (−12·6 to 0·4)	9·5 (−0·4 to 18·9)	0·48 (0·21 to 0·73)
Mali*	3·6 (−1·3 to 10·0)	−0·2 (−6·0 to 5·6)	6·7 (−3·2 to 17·6)	0·16 (0·00 to 0·37)
Mauritania*	4·3 (−1·5 to 11·8)	−0·4 (−7·2 to 6·3)	6·7 (−2·9 to 17·5)	0·03 (0·00 to 0·08)
Mongolia*	2·5 (−9·5 to 14·0)	−1·0 (−7·7 to 6·6)	2·0 (−9·9 to 11·8)	0·02 (−0·04 to 0·08)
Mozambique*	15·7 (4·6 to 23·0)	−2·5 (−7·9 to 3·3)	19·2 (5·5 to 28·7)	0·77 (0·28 to 1·09)
Myanmar*	5·9 (−4·9 to 16·5)	−2·5 (−8·4 to 3·4)	5·2 (−5·0 to 15·5)	0·61 (−0·32 to 1·53)
Nepal*	5·4 (−5·7 to 16·3)	−4·4 (−11·9 to 3·5)	6·2 (−5·5 to 17·0)	0·62 (−0·06 to 1·28)
Nicaragua	0·7 (−8·7 to 7·8)	−0·4 (−4·4 to 5·2)	0·5 (−6·6 to 5·2)	0·06 (−0·04 to 0·13)
Niger*	2·6 (−1·4 to 7·3)	2·2 (−2·1 to 7·4)	1·9 (−6·6 to 10·9)	0·20 (0·04 to 0·40)
Nigeria*	2·7 (−0·9 to 7·1)	2·8 (−2·3 to 8·2)	2·4 (−5·5 to 11·0)	1·11 (0·06 to 2·42)
North Korea	1·1 (−10·1 to 11·1)	−0·7 (−6·9 to 6·7)	1·0 (−9·3 to 9·2)	0·02 (−0·47 to 0·45)
Pakistan*	5·2 (−2·2 to 13·1)	−0·9 (−7·3 to 6·1)	5·4 (−4·8 to 15·5)	2·45 (0·16 to 4·95)
Palestine*	2·5 (−9·3 to 14·9)	−0·8 (−8·9 to 8·3)	2·0 (−11·3 to 14·2)	0·05 (−0·02 to 0·12)
Papua New Guinea	1·9 (−8·0 to 13·7)	−1·0 (−8·8 to 6·3)	2·4 (−10·1 to 15·7)	0·06 (−0·07 to 0·22)
Philippines*	4·1 (−7·7 to 16·5)	−2·4 (−11·1 to 7·5)	4·3 (−9·8 to 17·2)	1·12 (−0·77 to 3·12)
Rwanda*	4·5 (−5·5 to 14·3)	−3·6 (−10·0 to 3·2)	5·4 (−5·1 to 15·0)	0·14 (−0·01 to 0·29)
São Tomé and Príncipe*	6·0 (−6·0 to 17·6)	−5·0 (−13·5 to 4·0)	7·1 (−5·8 to 18·9)	0·00 (0·00 to 0·01)
Senegal*	6·4 (0·0 to 14·3)	−3·0 (−8·2 to 2·4)	10·8 (0·9 to 21·2)	0·20 (0·05 to 0·40)
Sierra Leone*	6·4 (−0·4 to 13·5)	−0·4 (−6·3 to 5·8)	9·5 (−1·7 to 20·3)	0·08 (0·01 to 0·15)
Solomon Islands	1·8 (−8·5 to 13·6)	−0·4 (−7·4 to 6·4)	1·7 (−11·1 to 14·5)	0·00 (−0·01 to 0·02)
Somalia	0·7 (−0·4 to 3·9)	0·2 (−6·9 to 7·5)	1·8 (−1·5 to 9·2)	0·01 (0·00 to 0·06)
South Sudan	0·8 (−0·7 to 4·1)	0·4 (−6·5 to 7·4)	2·2 (−2·3 to 10·0)	0·03 (0·00 to 0·10)
Sri Lanka	1·9 (−11·0 to 14·3)	−1·1 (−10·7 to 9·4)	1·6 (−11·9 to 14·0)	0·05 (−0·39 to 0·48)
Sudan*	2·3 (−2·9 to 9·3)	0·1 (−6·5 to 6·7)	3·9 (−6·2 to 14·7)	0·20 (−0·11 to 0·65)
Tajikistan	3·5 (−6·6 to 15·5)	−0·5 (−6·2 to 5·5)	3·4 (−9·5 to 15·8)	0·09 (−0·07 to 0·28)
Timor-Leste*	3·1 (−4·1 to 10·2)	−1·7 (−7·1 to 3·7)	4·7 (−5·0 to 14·5)	0·00 (−0·01 to 0·01)
Togo*	4·6 (−2·2 to 11·1)	−1·9 (−8·2 to 4·9)	6·8 (−3·1 to 15·9)	0·08 (0·00 to 0·16)
Uganda*	7·3 (0·0 to 14·5)	−4·1 (−9·6 to 1·5)	8·8 (0·0 to 17·5)	0·66 (0·27 to 1·04)
Tanzania*	5·4 (−3·8 to 14·6)	−2·3 (−8·2 to 3·7)	6·2 (−4·8 to 16·6)	0·72 (0·03 to 1·42)
Uzbekistan	1·2 (−10·3 to 11·6)	−0·4 (−5·7 to 5·7)	0·6 (−8·5 to 8·3)	0·30 (−0·35 to 0·89)
Vietnam*	−0·2 (−11·2 to 9·3)	0·4 (−6·1 to 9·4)	−0·5 (−11·4 to 7·7)	0·59 (−1·49 to 2·37)
Yemen	5·4 (−5·4 to 17·3)	−2·2 (−9·5 to 5·3)	5·8 (−7·0 to 19·2)	0·40 (−0·05 to 0·90)
Zambia*	6·3 (−6·0 to 18·4)	−3·9 (−11·1 to 3·8)	6·6 (−5·8 to 18·3)	0·29 (0·01 to 0·56)
Zimbabwe*	5·6 (−4·3 to 14·1)	−2·7 (−7·2 to 3·0)	4·2 (−4·2 to 10·8)	0·35 (0·08 to 0·56)

Data in parentheses are 95% uncertainty intervals (UIs).

* Country with survey or service statistics data available after 2012.

† Results for Burundi excluding the 2012 national survey: change in mCPR, 4·5 (95% UI −1·8 to 11·1); change in unmet need, −1·0 (−6·7 to 4·7); change in demand satisfied, 5·6 (−3·1 to 14·3); and change in number of women using modern contraception, 0·12 million (0·02 to 0·22).

**Figure 4:**
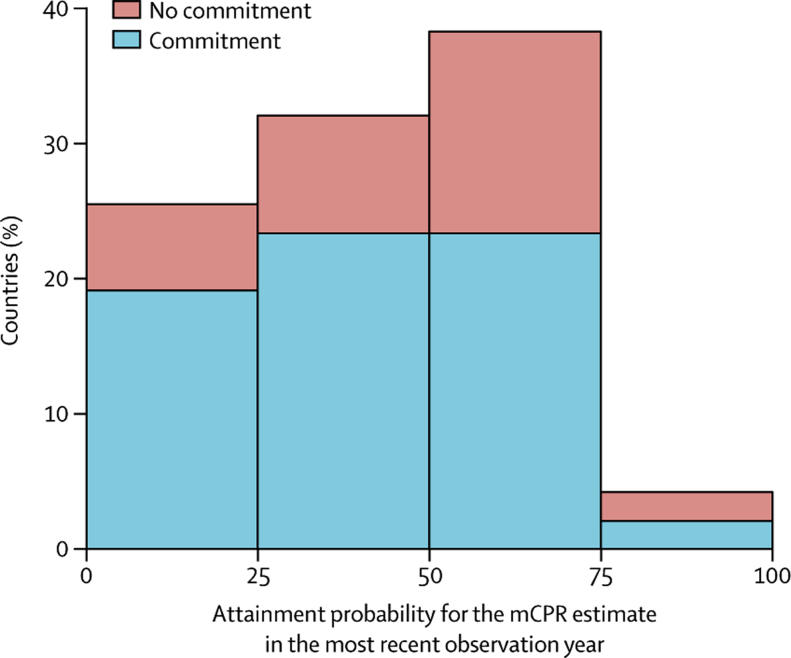
Attainment probabilities for modern contraceptive prevalence (mCPR) estimates in the most recent observation year for countries that made a commitment or not to FP2020 Results are for countries that had data available after 2012.

Table 3 shows the counterfactual analysis of changes in mCPR since 2012. The counterfactual analysis is presented for the 47 countries with data after 2012, 33 of which have made commitments to FP2020 (appendix p 5). The distribution of FP2020 commitment countries versus non-commitment countries shown in figure 4 shows that overall, 27 (57%) countries had levels of mCPR greater than or equal to pre-FP2020 expectations (ie, in 2012 the probability that they would reach current levels of mCPR was ≤50%), including 20 (61%) of the commitment countries in the analysis. The distribution also shows that 12 (26%) countries reached levels of mCPR that had less than or equal to 25% probability of attainment pre-FP2020. Conversely, only two (4%) dropped to levels of mCPR that had greater than 75% probability of attainment pre-FP2020. Among the countries that we consider to have made the most progress relative to what was expected are Chad, Sierra Leone, Mozambique, and Kenya, of which the latter three have made commitments to FP2020. These four countries attained levels of mCPR post-2012 that had a less than 10% chance of being observed pre-FP2020. In Kenya, the estimate for mCPR in 2015, the most recent observation year was 14·3 percentage points higher than the pre-FP2020 expectations for this year. This estimate equates to an additional 0·92 million women of reproductive age who are married or in a union using modern methods of contraception in 2015 compared with what was expected. Conversely, Burundi, which is a commitment country and Gambia, a non-commitment country, had lower levels of mCPR relative to expectations with a greater than 90% chance that the mCPR would be higher than current estimates. In terms of absolute numbers, the drop in mCPR in Burundi in 2016 relative to what was expected equates to 0·15 million women of reproductive age who are married or in a union not using modern methods of contraception. Excluding the 2012 national survey from the counterfactual analysis for Burundi still results in the 2016 estimate for mCPR being lower than 2012 expectations; however without this survey the median difference would be 1·3 percentage points rather than 9·5 percentage points.

**Table 3:** Results of a counterfactual analysis of changes in modern contraceptive prevalence rate (mCPR) since 2012

	**Year of most recent data**	Estimated mCPR (%)—pre-FP2020 expectation for mCPR (%)*	**Estimated number of women using modern methods (millions)—pre-FP2020 expectation for the number of women using modern methods (millions)**†	**Attainment probability for estimated mCPR (%)**‡	**Country commitment to FP2020**
**≤25% attainment probability**					
Mozambique	2016	14·1	0·61	0	Yes
Kenya	2015	14·3	0·92	2	Yes
Sierra Leone	2016	7·7	0·08	3	Yes
Chad	2014	1·4	0·03	7	No
Liberia	2016	7·7	0·05	10	Yes
Lesotho	2014	7·5	0·02	11	No
Malawi	2015	8·4	0·21	11	Yes
Senegal	2015	3·2	0·08	15	Yes
Zimbabwe	2016	5·2	0·13	18	Yes
Burkina Faso	2017	4·6	0·15	19	Yes
São Tomé and Príncipe	2014	5·7	0·00	22	No
Benin	2015	1·5	0·03	25	Yes
**26–74% attainment probability**					
Mali	2015	1·5	0·05	26	Yes
Mauritania	2015	1·8	0·01	32	Yes
Zambia	2013	3·4	0·07	32	Yes
Cameroon	2016	1·5	0·05	36	Yes
Ethiopia	2016	2·1	0·31	38	Yes
Kyrgyzstan	2014	1·3	0·01	38	No
Palestine	2014	1·2	0·01	43	No
Myanmar	2016	1·1	0·10	44	Yes
Guinea-Bissau	2014	0·5	0·00	45	No
Togo	2016	0·3	0·00	45	Yes
Afghanistan	2016	0·3	0·02	47	Yes
Philippines	2013	0·3	0·05	47	Yes
Ghana	2016	0·2	0·01	49	Yes
Uganda	2016	0·1	0·01	49	Yes
Sudan	2014	0·0	0·00	50	No
Nigeria	2017	−0·2	−0·05	51	Yes
Nepal	2015	−0·4	−0·02	53	Yes
Pakistan	2016	−0·1	−0·02	53	No
Niger	2016	−0·4	−0·01	54	Yes
Côte d'Ivoire	2016	−0·6	−0·02	56	Yes
Tanzania	2016	−1·4	−0·11	57	Yes
Cambodia	2014	−1·2	−0·03	58	No
India	2016	−1·5	−3·69	58	Yes
Yemen	2013	−1·7	−0·07	58	No
Bangladesh	2014	−1·0	−0·37	59	Yes
Vietnam	2014	−0·7	−0·12	60	Yes
Mongolia	2013	−1·9	−0·01	63	No
Rwanda	2016	−2·3	−0·03	63	Yes
Timor-Leste	2016	−2·9	0·00	67	No
Congo (Brazzaville)	2014	−2·0	−0·01	68	No
Egypt	2014	−4·0	−0·64	72	No
Democratic Republic of the Congo	2013	−1·6	−0·19	74	Yes
Indonesia	2015	−3·2	−1·59	74	Yes
**≥75% attainment probability**					
Burundi	2016	−9·5	−0·15	96	Yes
Gambia	2013	−6·2	−0·02	97	No

Countries are listed according to increasing attainment probability.

* Difference between the current point estimate for mCPR and the counterfactual point estimate for mCPR (pre-FP2020 expectation) in the most recent observation year.

† Difference between the current point estimate for the number of married or in union women aged between 15 and 49 years using modern methods of contraception and the counterfactual point estimate (pre-FP2020 expectation) in the most recent observation year.

‡ Pre-FP2020 probabilities of attaining the current estimates of mCPR for the most recent observation year.

## Discussion

At the regional level, median mCPR growth across Asian countries has been less than 1·0 percentage point since 2012. The more positive FP2020 results are occurring across the African continent where much of the overall increase in population between now and 2050 is projected to occur.^15^ Since 2012, mCPR in Africa has increased from 23·9% (95% UI 22·9–25·0) in 2012 to 28·5% (26·8–30·2) in 2017, with the fastest growth occurring in east Africa. In particular for east Africa, Mozambique and Kenya, both of which have made commitments to FP2020, can be seen to have made the most progress of all the countries in the initiative both in terms of increasing mCPR and demand satisfied and exceeding pre-FP2020 expectations. Within west Africa, Nigeria, which currently has the most rapidly growing population among the ten largest countries in the world,^15^ has seen a change in mCPR of 2·7 percentage points (95% UI −0·9 to 7·1) between 2012 and 2017 (0·54 points per year; table 1).

Kenya stood out both in terms of reducing unmet need, increasing mCPR, and exceeding pre-FP2020 expectations and can provide a case study for achieving success in other countries. For example, it is likely that the success in Kenya can be partly attributed to Tupange, a programme to scale up family planning services by integrating family planning into existing health services and working with Kenyan health officials and community groups.^16^ The programme ran up to 2014 and implemented tools such as commodity security and service delivery to improve the availability of family planning commodities as service delivery points and to improve service provider capacity, quality of service, and choice of methods to the lowest level of health care.

In this analysis, the FPET provided probabilistic estimates and projections allowing us to assess the current levels of key FP2020 indicators, as well as current and future progress, with uncertainty quantification. The objective of this analysis was not to undertake a rigorous impact evaluation of the FP2020 global initiative, as the degree to which family planning programmes can affect the pace of growth in mCPR is difficult to quantify and will depend on a number of factors. Such factors might include the amount of exposure people are receiving towards family planning within a country, whether the exposure was positive and resulted in an adoption of a family planning method and hence resulted in mCPR growth, and the willingness of people to communicate new information and positive experiences to promote further growth. Providing answers to these questions would involve a different analytical design to what we have presented here, as well as the inclusion of a more comprehensive set of explanatory indicators and covariates. The purpose of this study was to show the advantage of using a statistical approach and the information that can be obtained by estimating and projecting trends for key FP2020 indicators at the aggregate and country levels. At the country level, using FPET estimates, we have determined the countries that have seen positive changes since the beginning of FP2020 as well as the countries that appear to be lagging behind. Furthermore, pre-FP2020 counterfactual projections allowed us to highlight the countries that have exceeded pre-FP2020 expectations. This resulted in highlighting countries such as Chad. In Chad, a focus on the increase in mCPR alone (which is relatively small) would not reflect that this increase was greater than expected based on past trends. An area for future work is an assessment of the contribution of specific contraceptive methods to changes in mCPR to provide insight into what methods in particular are driving the changes.

The analysis is not without limitations, particularly with respect to the availability of recent data. 21 of the 68 countries in this analysis lack any survey or service statistics data (or both) after 2012 resulting in less information to inform projections. For a country without recent data, projections become more reliant on past experience as well as on past experiences in other nearby countries (appendix p 12). Hence, current estimates in these countries are subject to change due to the addition of new data as well as due to possible model changes. Of the 47 countries that have data after 2012, two are relying only on service statistics rather than surveys or a combination of both to inform recent trends. Furthermore, surveys including the DHS and PMA2020 are subject to non-sampling error^17^, ^18^ and future analysis is necessary to better understand the direction and size of such errors in diverse settings. The lack of recent data and uncertainty associated with available data results in uncertainty surrounding the estimates that might be substantial. This is true in particular for the uncertainty associated with additional users (which is based on changes in mCPR), which depends on the uncertainty associated with recent mCPR levels. In addition to data limitations, we recognise that producing national estimates of family planning indicators might mask diversity at the subnational level, particularly in highly populated countries such as Nigeria and India. However, with the exception of India (for reasons stated below) progress for FP2020 is currently only monitored at a national level. Lastly, in this study we focus only on women who are married or in a union. The upcoming FP2020 2017 report^19^ will present progress for all women in the FP2020 countries.

In India, which accounts for more than 40% of all modern method users in the 69 FP2020 countries, we had the ability to apply the FPET at the state level and provide national totals that reflect changes in sub-national trends.^20^ In 2015–16, the fourth round of NFHS was done covering around 800 000 men and women. The preliminary results showed substantial declines of up to 15 percentage points in mCPR for several states including Gujarat, Bihar, Arunachal Pradesh, Karnataka, and Madhya Pradesh. Since the full datasets have not yet been released it is difficult to understand if the declines are real or due to methodological problems. The situation was reviewed at a meeting of national technical experts in New Delhi in April, 2017.^21^ The experts recommended that, until the datasets are released, the NFHS-4 results should be excluded from the estimates for any state in which they fall outside 2 SDs from the projected mCPR trend for 2016. Given how important trends in India are to the aggregate trends for all countries, we have heeded this advice. Thus, the India national aggregate estimates of family planning indicators include NFHS-4 results for all states except four of the five mentioned above. For Bihar, the NFHS-4 result was included along with the estimates from the Bihar national survey of women of reproductive age who are married or in a union and the Sector Wide Approach to Strengthen Health Survey, 2015–16. The estimates will be revised once the final datasets are released and final estimates can be studied in detail.

It remains to be seen if the FP2020 initiative will achieve its 120×20 goal. Our results show that progress is being made for women of reproductive age who are married or in a union, who make up almost three quarters of the total number of additional users of modern methods of contraception estimated between 2012 and 2017 at 38·8 million,^19^ but the rate of change is not as fast as desired. However, we know that secular changes in behaviours like contraceptive use across vast populations do not change rapidly over an 8 year period. We recognise that the animation of the international family planning field after the London Summit in 2012 did not immediately result in implementation of new programmes and new interventions. Rather, the global family planning community has arguably still not assembled the critical mass of new programmes, policies, and interventions in the field that are commensurate with the ambitious 120×20 goal. Additionally, despite funding pledges, funding has probably not increased substantially since 2012, as is suggested in the recent FP2020 report^19^ and is inadequate.^22^ Analyses such as those we have presented here can serve to re-ignite commitment toward achieving the 120×20 goal and place focus on the countries and regions where intervention is needed the most. At the follow-up London Family Planning Summit of July 11, 2017, policy makers, countries, donors, civil society, and the private sector committed new resources to the effort, and the overall mediocre estimates of progress has the potential to be offset by a renewed energy and commitment directed toward FP2020 at global and country levels.^23^
