# Supplementary material for: Modern contraceptive use, unmet need, and demand satisfied among women of reproductive age who are married or in a union in the focus countries of the Family Planning 2020 initiative: a systematic analysis using the Family Planning Estimation Tool
Source: Lancet. 2018 Mar 3;391(10123):870–82. doi: 10.1016/S0140-6736(17)33104-5 (PMC5854461; doi:10.1016/S0140-6736(17)33104-5)
Supplement: Supplementary appendix [file mmc1.pdf]

THE LANCET

Supplementary appendix

This appendix formed part of the original submission and has been peer reviewed. We post it as supplied by the authors.

Supplement to: Cahill N, Sonneveldt E, Stover J, et al. Modern contraceptive use, unmet need, and demand satisfied among women of reproductive age who are married or in a union in the focus countries of the Family Planning 2020 initiative: a systematic analysis using the Family Planning Estimation Tool. *Lancet* 2017; published online Dec 5. http://dx.doi.org/10.1016/S0140-6736(17)33104-5.

Contraceptive use, unmet need and demand satisfied among women of reproductive age who are married or in a union in the focus countries of the Family Planning 2020 Initiative: a systematic analysis using the Family Planning Estimation Tool

Supplementary webappendix

Niamh Cahill[1], Emily Sonneveldt[2], John Stover[2], Michelle Weinberger[3], Jessica Williamson[3], Chuchu Wei[1], Win Brown[4], and Leontine Alkema[1]

[1]Department of Biostatistics and Epidemiology, University of Massachusetts-Amherst, Amherst, USA
[2]Avenir Health, Washington D.C., USA
[3]Avenir Health, Glastonbury, USA

[4]Bill & Melinda Gates Foundation, Seattle, USA

Contents

1 Data **3**

1.1 Definitions of regions and sub-regions . . . 3

1.2 Survey data on contraceptive prevalence and unmet need for family planning . . . 3

1.3 FP2020 Countries and Database . . . 4

1.4 Women of reproductive age (15-49) who are married or in a union . . . 8

2 Overview of The Family Planning Estimation Model **8**

2.1 Modeling time trends in contraceptive prevalence . . . 8

2.2 Technical specification of the model for $P_{c,t}$ . . . 9

2.2.1 Model specification for the expected rate of change $\delta_{c,t}$ . . . 10

2.3 Bayesian hierarchical model . . . 12

2.4 The data model . . . 13

2.5 Survey specific standard errors . . . 14

2.6 Reference periods of observations . . . 15

2.7 Prior distributions . . . 16

2.8 Non-sampling error-variance parameters . . . 16

3 A country specific implementation of FPEM **17**

4 Utilizing Service Statistics Data **18**

4.1 Data . . . 18

4.2 Deriving Estimated Modern Use (EMU) . . . 18

4.3 Using EMU data in FPET . . . 20

5 Counter-factual projections and mCPR attainment probabilities **21**

6 References **22**

7 Supplementary Table **24**

8 Supplementary Figures **25**

Throughout this appendix, where relevant and required, information from Alkema et al., 2013 [1] that describes the family planning estimation model (FPEM) is copied directly.

1 Data

1.1 Definitions of regions and sub-regions

The definition of regions and sub-regions follows that implemented in the United Nations Standard Country or Area Codes for Statistical Use [2]. The terms 'developed countries' and 'developing countries' are used for statistical convenience and do not necessarily express a judgment about the stage reached by a particular country or area in the development process. The term 'country' as used in this study also refers, as appropriate, to territories or areas.

1.2 Survey data on contraceptive prevalence and unmet need for family planning

Contraceptive prevalence is measured as the percentage of women who report themselves or their partners as currently using at least one contraceptive method of any type (modern or traditional). Modern methods of contraception include female and male sterilization, oral hormonal pills, the intra-uterine device (IUD), male and female condoms, injectables, the implant (including Norplant), vaginal barrier methods and emergency contraception. Traditional methods of contraception include rhythm (periodic abstinence), withdrawal, prolonged abstinence, breastfeeding, douching, lactational amenorrhea method (LAM) and folk methods.

Unmet need for family planning is defined as the percentage of women who want to stop or delay childbearing but are not using any method of contraception to prevent pregnancy. Measurements of unmet need for family planning requires survey data on desire for another birth and its timing, pregnancy, post-partum amenorrhea, infecundity, and wantedness of a current pregnancy or last live birth and are, whenever possible, based on the revised algorithm of the indicator designed to improve comparability within and across countries [3].

The contraceptive prevalence indicator is generally based on nationally representative household surveys with questions on current use of contraception. Surveys that commonly include this information are: Demographic and Health Surveys (DHS), Reproductive Health Surveys (RHS), Multiple Indicator Cluster Surveys (MICS), Performance Monitoring and Accountability 2020 (PMA 2020) surveys and other national surveys. On average, surveys are undertaken every three to five years.

UNPD Database Data on contraceptive prevalence and unmet need for family planning are assessed and compiled on a regular basis by the United Nations Population Division. The most recent database is World Contraceptive Use 2017 [4]. The UNPD database includes 1119 observations on contraceptive prevalence (any method) for 195 countries and areas. Observations from the database are excluded in cases where they are not related to the current use of contraception, but pertain to 'past or current use' or to 'method used since the last

pregnancy'. Figure 1 gives an overview on data availability by region and period, for observations on total prevalence and unmet need.

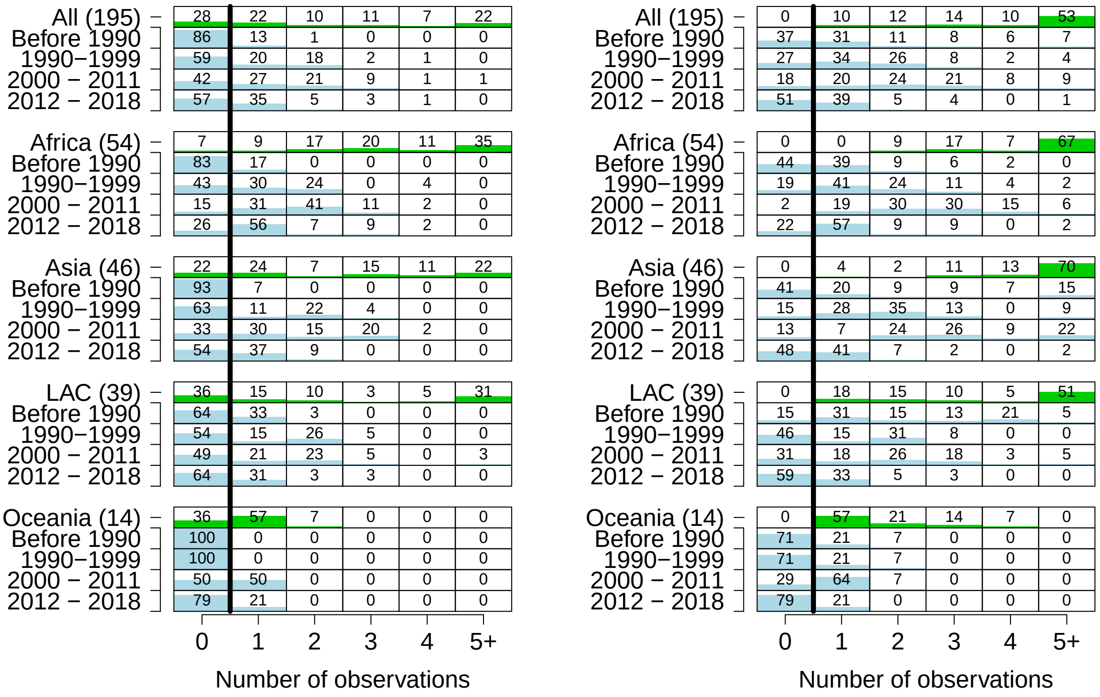

Figure 1: Overview of the percentage of countries in the UNPD database with 0,1,2,3,4 or 5+ observations on total contraceptive prevalence (left) or unmet need (right), summarized for all countries, for all developed countries combined, and by region for the developing countries. The first row (green) for each group of countries refers to the entire observation period; each cell contains the percentage of countries with 0,1,2,3,4 or 5+ observations. Similarly, subsequent rows (blue) show the percentage of countries by number of observations in the period before 1990, from 1990 to 1999, from 2000 to 2011 and from 2012 to 2016. The colored shading visualizes the percentage of countries in each cell. The number with the subgroup refers to the total number of countries within that subgroup. LAC refers to Latin America and the Caribbean.

1.3 FP2020 Countries and Database

The FP2020 database The survey database used by Track20 [5] is a subset of the UNPD global database and includes 519 survey observations of contraceptive prevalence from 68 countries (Figure 2) with the addition of 217 state level survey observations for India (Table 1). Of these 69 FP2020 countries (shown below), 37 have made a pledge to the FP2020 initiative (labeled with a $*$). Countries labeled with a § have survey data available after 2012.

Eastern and Southern Africa

- Burundi*§
- Comoros
- Djibouti
- Eritrea
- Ethiopia*§
- Kenya*§
- Lesotho§
- Madagascar*
- Malawi*§
- Mozambique*§
- Rwanda*§
- Somalia*
- Tanzania*
- Uganda*§
- Zambia*§
- Zimbabwe*§

Central Africa

- Cameroon*§
- Central African Republic
- Chad§
- Congo§
- DR Congo§
- Sao Tome and Principe

Western Africa

- Benin*§
- Burkina Faso*§
- Cote dIvoire*§
- Gambia§
- Ghana*§
- Guinea*
- Guinea-Bissau§
- Liberia*§
- Mali*§
- Mauritania*§
- Niger*§
- Nigeria*§
- Senegal*§
- Sierra Leone*§
- Togo*§

Middle East and Northern Africa

- Egypt§
- Iraq
- South Sudan
- State of Palestine§
- Sudan§
- Western Sahara [a]
- Yemen§

[a] Western Sahara which has no data, has been left out of the analysis, as it is not currently included in the reporting for the initiative.

Eastern and Central Asia

- Kyrgyzstan§
- Mongolia§
- DPR Korea
- Tajikistan
- Uzbekistan

South Asia

- Afghanistan*§
- Bangladesh*§
- Bhutan
- India*§
- Nepal*§
- Pakistan*§
- Sri Lanka

South East Asia and Oceania

- Cambodia§
- Indonesia*§
- Lao PDR*
- Myanmar*§
- Papua New Guinea
- Philippines*§
- Solomon Islands*
- Timor-Leste§
- Vietnam*§

Latin America and Caribbean

- Bolivia
- Haiti
- Honduras
- Nicaragua

Data series	Number of states/UTs with an observation
Annual Health Survey 2010-2011	9
Annual Health Survey 2011-2012	9
Annual Health Survey 2012-2013	9
District Level Household and Facility Survey 1998-1999	1
District Level Household and Facility Survey 2002-2004	30
District Level Household and Facility Survey 2007-2008	29
District Level Household and Facility Survey 2012-2013	18
National Family Health Survey 1992-1993	25
National Family Health Survey 1998-1999	25
National Family Health Survey 2005-2006	29
National Family Health Survey 2015-2016	28
Other National surveys 2014-2016	3
Performance Monitoring and Accountability 2016-2017	2
Total number of observations	217

Table 1: An overview of the survey observations available at the subnational (state/UT-level) in India

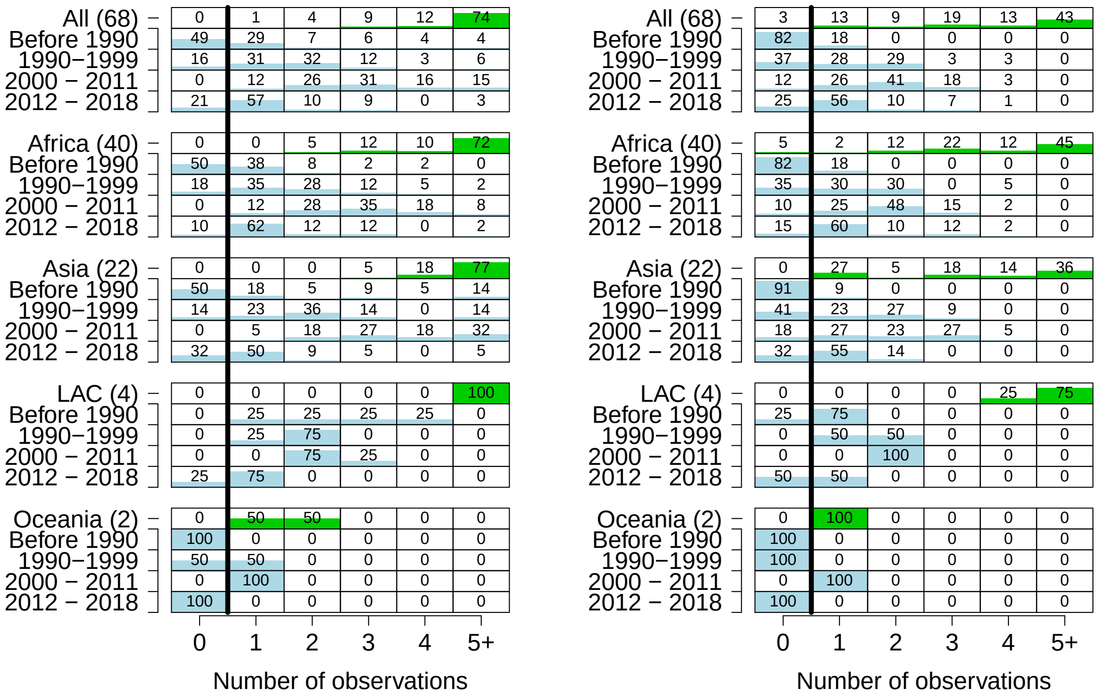

Figure 2: Overview of the percentage of countries in the FP2020 database with 0,1,2,3,4 or 5+ observations on total contraceptive prevalence (left) or unmet need (right), summarized for all countries, and by region. The first row (green) for each group of countries refers to the entire observation period; each cell contains the percentage of countries with 0,1,2,3,4 or 5+ observations. Similarly, subsequent rows (blue) show the percentage of countries by number of observations in the period before 1990, from 1990 to 1999, from 2000 to 2011 and from 2012 to 2016. The colored shading visualizes the percentage of countries in each cell. The number with the subgroup refers to the total number of countries within that subgroup. LAC refers to Latin America and the Caribbean.

Service Statistics Service Statistics (SS) enable countries to use their administrative data to track contraceptive use. SS data systems (e.g., the District Health Information Software) report data related to the distribution of short-term contraceptive methods, such as oral pills, condoms, and injectables, and long-acting and permanent methods, such as IUDs, implants, and sterilisation. However, the data are prone to under reporting from the private sector, as well as other limitations, preventing it from being used as a reliable indicator for the level of modern contraceptive prevalence. As a result, SS data are only currently used to provide a proxy for modern contraceptive prevalence, termed estimated modern use (EMU), that is assumed biased with respect to the true level of the indicator [5]. The Track20 survey database is supplemented with with 85 service statistic observations, from 13 countries, which are deemed reliable enough to inform estimates and projections of modern contraceptive use. Section 4 provides further technical details for the use of SS data.

1.4 Women of reproductive age (15-49) who are married or in a union

Estimates of contraceptive prevalence were constructed for the base population of women who are married or in a union and of reproductive age (MWRA). Estimates and projections of the number of women of reproductive age were provided by the United Nations population division using the same methods as described in Alkema et al., 2013 [1] with updated population and marital status data [6, 7].

2 Overview of The Family Planning Estimation Model

The goal of FPEM is the categorization of MWRA into four groups (Figure 3), women who use traditional contraceptive methods, women who use modern contraceptive methods, women who have unmet need for contraceptive methods, and women who do not need any method (who are not avoiding a pregnancy). In other words, the outcomes of interest are a compositional vector, here denoted by $p_{c,t} = (p_{c,t,1}, p_{c,t,2}, p_{c,t,3}, p_{c,t,4})$, where $p_{c,t,m}$ denotes the proportions of women in country c, in year t, who use traditional methods $(m = 1)$, modern methods $(m = 2)$, have unmet need for contraceptive methods $(m = 3)$, or do not use and do not need contraceptive methods $(m = 4)$. Compositional vector $p_{c,t}$ is not observed, instead we observe $y_i = y_{i,1:4}$, where $y_{i,m}$ denotes the proportion of women in category m for observation $i = 1, \ldots, I$ for country $c[i]$ and year $t[i]$.

2.1 Modeling time trends in contraceptive prevalence

Modeling components of the compositional vector The components of compositional vector $p_{c,t}$ are restricted to add up to one and cannot be modeled independently; independent models for each component $p_{c,t,m}$ for $m = 1, 2, 3, 4$ would not guarantee that they add up to one. We guarantee that the components do add up to one by modeling total contraceptive method use over time, to then categorize the women who use any contraceptive method into modern and traditional methods, and the women who do not use any method into women with unmet need and women without need for contraceptive methods. Specifically, if $P_{c,t}$ denotes total contraceptive prevalence, $R_{c,t}$ the ratio of modern to total prevalence, and $Z_{c,t}$ the ratio of unmet need to no contraceptive use in country c, year t,

$$
\begin{aligned}
P_{c,t} &= p_{c,t,1} + p_{c,t,2}, \\
R_{c,t} &= \frac{p_{c,t,2}}{p_{c,t,1} + p_{c,t,2}}, \\
Z_{c,t} &= \frac{p_{c,t,3}}{p_{c,t,3} + p_{c,t,4}}.
\end{aligned}
$$

$R_{c,t}$ and $Z_{c,t}$ are modeled as they are in Alkema et al., 2013 [1]. These quantities are modeled by systematic (latent) trends with autocorrelated distortions added to them. The specification of $P_{c,t}$ is explained below.

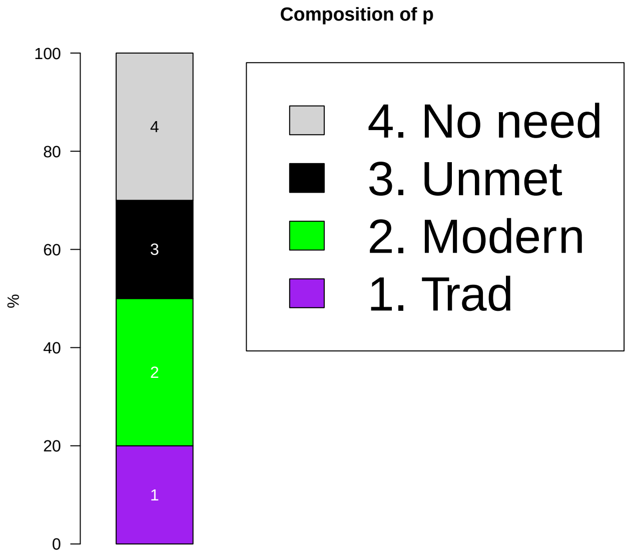

Figure 3: Illustration of composition $p_{c,t} = p_{c,t,1:4}$. Categorization of women who use traditional contraceptive methods, women who use modern contraceptive methods, women who have unmet need for contraceptive methods, and women who do not need any method (who are not avoiding a pregnancy).

2.2 Technical specification of the model for $P_{c,t}$

In line with Alkema et al., 2013, systematic trends in contraceptive prevalence are captured by logistic growth curves. An example of a logistic growth curve $P^*_{c,t}$ to capture changes in contraceptive use is given in Figure 4, where

$$P^*_{c,t} = \frac{\tilde{P}_c}{1 + exp(-\omega_c(t - \Omega_c))}, \tag{1}$$

with pace parameter ω_c, timing determined by Ω_c and contraceptive use asymptote $\tilde{P}_c$. Figure 4 also illustrates the corresponding growth of logit-transformed prevalence

$$S^*_{c,t} = logit(P^*_{c,t}), \tag{2}$$

and the annual differences in logit-transformed prevalence $S^*_{c,t} - S^*_{c,t-1}$. The latter, referred to as the rate of change, decreases to zero as $P^*_{c,t}$ increases to $\tilde{P}_c$.

The model for total prevalence $P_{c,t}$ takes account of logistic growth and deviations away from logistic growth as follows:

$$logit(P_{c,t}) \quad = \quad logit(P_{c,t-1}) + \delta_{c,t} + \epsilon_{c,t}, \tag{3}$$

where $\delta_{c,t}$ refers to an expected rate of change that captures logistic growth as explained in more detail below, and $\epsilon_{c,t}$ refers to an autocorrelation distortion term that captures the deviation away from the expected rate of change. The deviations are captured with an AR(1) model:

$$\epsilon_{c,t} \sim N(\rho_\epsilon \cdot \epsilon_{c,t-1}, \tau_\epsilon^2). \quad (4)$$

Equations 3 and 4 specify changes in total contraceptive use $P_{c,t}$ but not its level. Hence, we complete the specification by including P_{c,t^*} for some year t^* (here chosen to be 1990) as a model parameter.

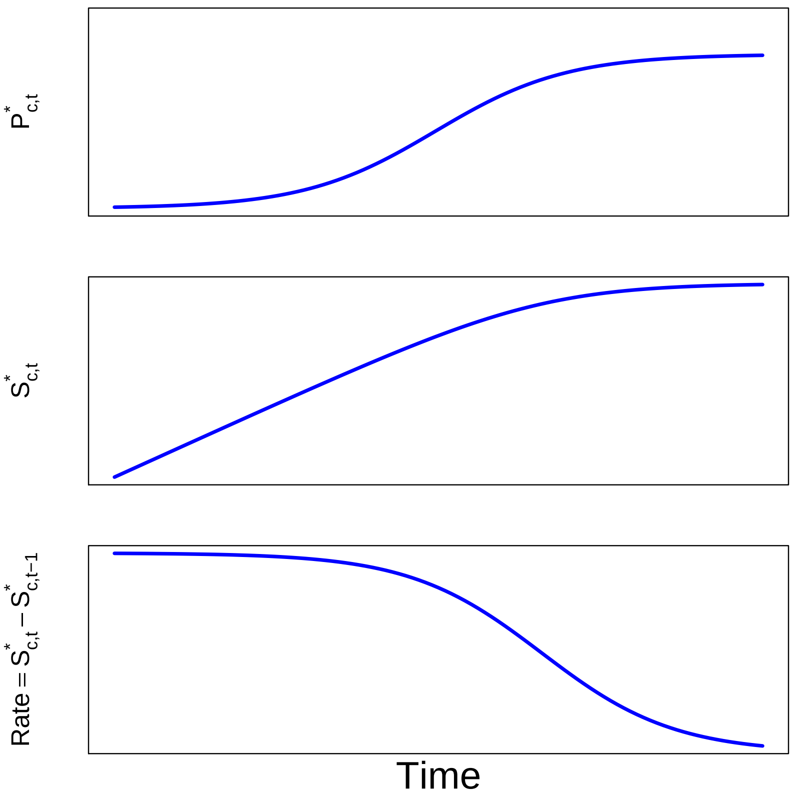

Figure 4: An example of a logistic growth curve (top panel), a logistic growth curve on the logit scale (middle panel) and the resulting rate curve on the logit scale (bottom panel) for total contraceptive prevalence.

2.2.1 Model specification for the expected rate of change $\delta_{c,t}$

The expected change in $P_{c,t}$ on the logit scale, $\delta_{c,t}$, is defined such that without deviations away from the expected rate of change, $P_{c,t}$ follows the logistic growth curve as specified in Eq. 1. In other words, if $\epsilon_{c,t} = 0$ for all years, then

$$\delta_{c,t} = logit(P^*_{c,t}) - logit(P^*_{c,t-1}),$$

where $P^*_{c,t}$ is defined as in Eq. 1 (such that in this setting, $P_{c,t}$ would be equal to $P^*_{c,t}$).

We first explain the parametrization of $\delta_{c,t}$ and $P_{c,t}$ in the simplified set-up with an asymptote of contraceptive use $\tilde{P}_c = 1$, and then provide the details for the final setting with $\tilde{P}_c < 1$ (which is more realistic given that the contraceptive use asymptote will never be at 100%).

Model specification for $P_{c,t}$ when $\tilde{P}_c = 1$ (model simplification) With $\tilde{P}_c = 1$, Eq. 1 simplifies to

$$P^*_{c,t} = \frac{1}{1 + exp(-\omega_c(t - \Omega_c))},$$

and the rate of change on the logit scale associated with logistic growth is as follows:

$$logit(P^*_{c,t}) - logit(P^*_{c,t-1}) = \omega_c,$$

for any year t or prevalence level $P^*_{c,t-1}$. In this simplified setting, the model for total prevalence $P_{c,t}$ is defined as follows:

$$logit(P_{c,t}) \;\; = \;\; logit(P_{c,t-1}) + \delta_{c,t} + \epsilon_{c,t},$$

where $\delta_{c,t} = \omega_c$ such that, as desired, in absence of deviations (when $\epsilon_{c,t} = 0$), $P_{c,t}$ follows a logistic growth curve.

Model specification when $\tilde{P}_c < 1$ (final model used) When $\tilde{P}_c < 1$, the following holds true:

$$logit\left(P^*_{c,t}/\tilde{P}_c\right) - logit\left(P^*_{c,t-1}/\tilde{P}_c\right) = \omega_c.$$

or equivalently,

$$P^*_{c,t} = \tilde{P}_c \cdot logit^{-1}\left(logit\left(\frac{P^*_{c,t-1}}{\tilde{P}_c}\right) + \omega_c\right), \text{ for } P^*_{c,t-1} < \tilde{P}_c. \quad (5)$$

Based on Eq. 5, we find that

$$logit(P^*_{c,t}) - logit(P^*_{c,t-1}) =$$

$$\begin{cases} logit\left(\tilde{P}_c \cdot logit^{-1}\left(logit\left(\frac{P^*_{c,t-1}}{\tilde{P}_c}\right) + \omega_c\right)\right) - logit(P^*_{c,t-1}), & \text{when } P^*_{c,t-1} < \tilde{P}_c, \\ 0 & \text{otherwise.} \end{cases} \quad (6)$$

In this set-up, the expression for $logit\left(P^*_{c,t}\right) - logit\left(P^*_{c,t-1}\right)$ is not constant with time but depends on the level $P^*_{c,t-1}$, as illustrated in Figure 4. In the model for $P_{c,t}$, the expected rate of change $\delta_{c,t}$ is given by the left-hand side of Eq. 6, evaluated at $P_{c,t-1}$. As a result, (i) the expected rate of change in $P_{c,t}$ depends on its previous level $P_{c,t-1}$, as well as ω_c and $\tilde{P}_c$, and (ii), $P_{c,t}$ is given by $P^*_{c,t}$ in the absence of distortions. The remainder of this section provides the derivation of the expression of $P_{c,t}$ used.

To obtain the expression for the expected rate of change $\delta_{c,t}$ in Eq. 3 we replace $P^*_{c,t-1}$ in Eq. 6 by $P_{c,t-1}$ to obtain:

$$\delta_{c,t} = \begin{cases} logit\left(\tilde{P}_c \cdot logit^{-1}\left(logit\left(\frac{P_{c,t-1}}{\tilde{P}_c}\right) + \omega_c\right)\right) - logit(P_{c,t-1}), & \text{when } P_{c,t-1} < \tilde{P}_c, \\ 0 & \text{otherwise.} \end{cases} \quad (7)$$

The expression for $logit(P_{c,t})$ is thus given by:

$$logit(P_{c,t}) = logit(P_{c,t-1}) + \delta_{c,t} + \epsilon_{c,t} \quad (8)$$

$$= \begin{cases} logit\left(\tilde{P}_c \cdot logit^{-1}\left(logit\left(\frac{P_{c,t-1}}{\tilde{P}_c}\right) + \omega_c\right)\right) + \epsilon_{c,t}, & \text{when } P_{c,t-1} < \tilde{P}_c, \\ logit(P_{c,t-1}) + \epsilon_{c,t} & \text{otherwise.} \end{cases} \quad (9)$$

With similar reasoning, back-extrapolations (to obtain estimates $P_{c,t}$ for $t < t^*$) are obtained as follows:
$logit(P_{c,t}) =$

$$\begin{cases} logit\left(\tilde{P}_c \cdot logit^{-1}\left(logit\left(\frac{logit^{-1}(logit(P_{c,t+1})-\epsilon_t)}{\tilde{P}_c}\right) - \omega_c\right)\right), & \text{when } logit^{-1}(logit(P_{c,t+1}) - \epsilon_{c,t}) < \tilde{P}_c, \\ logit(P_{c,t+1}) - \epsilon_{c,t}, & \text{otherwise.} \end{cases}$$

2.3 Bayesian hierarchical model

Estimating country specific parameters presents a challenge because of the limited number of observations for each country. We used a Bayesian hierarchical model to estimate the parameters in each country, such that the estimates are based on the observations in the country of interest, as well as on the (sub)-regional and/or global experience. For classifying countries into (sub)-regions, we used the United Nations Population Division classification described earlier.

Different levels of hierarchy were used for different country parameters to best incorporate expected differences and similarities across countries and (sub)-regions. Country-specific asymptotes $\tilde{P}_c$ for total contraceptive prevalence were estimated with a hierarchical model with one level (world-country):

$$log\left(\frac{\tilde{P}_c - 0.5}{1 - \tilde{P}_c}\right) \sim N(\tilde{P}_w, \kappa_p^{(c)}),$$

where asymptotes were restricted to be between 50% and 100% and $\tilde{P}_w$ is the world mean and $\kappa_p^{(c)}$ the variance of the transformed $\tilde{P}_c$.

For pace parameters ω_c a three-level hierarchical model was used because these parameters are expected to vary across countries, sub-regions and regions. The transformation

$$\omega_c^* = log\left(\frac{\omega_c - 0.01}{0.5 - \omega_c}\right)$$

is used, such that ω_c is restricted to be between 0.01 and 0.5 (a reasonable range, corresponding to the transition from 10% to 90% of $\tilde{P}_c$ taking at least 10 years, and at most 4 centuries), and the hierarchical distributions are given by:

$$\omega_c^* \sim N(\omega_{s[c]}^*, \kappa_\omega^{(c)}),$$
$$\omega_s^* \sim N(\omega_{r[s]}^*, \kappa_\omega^{(s)}),$$
$$\omega_r^* \sim N(\omega_w^*, \kappa_\omega^{(r)}),$$

such that the (logit-transformed) ω_c's are distributed around a sub-regional mean $\omega_{s[c]}^*$ ($s[c]$ refers to the subregion of country c), and the sub-regional means are distributed around a regional mean $\omega_{r[s]}^*$ ($r[s]$ is the region of sub-region s). The variances on the country, sub-regional and regional level are given by $\kappa_\omega^{(c)}$, $\kappa_\omega^{(s)}$ and $\kappa_\omega^{(r)}$ respectively.

For P_{c,t^*}, the level of contraceptive prevalence at a fixed time point $t^* = 1990$, a three-level hierarchical model was used for developing countries (denoted $c \in L$) because the level is expected to vary across countries, sub-regions and regions. For developed countries (denoted $c \in D$) there is no distinction between regions; we do not expect differences in sub-regional means across developed countries. The transformation

$$S_{c,t^*} = logit(P_{c,t^*})$$

is used, such the the level is estimated on the logit scale. The hierarchical distributions are given by:

$$S_{c,t^*} \sim N(S_{s[c],t^*}, \kappa_S^{(c)}), \qquad c \in L,$$
$$S_{s,t^*} \sim N(S_{r[s],t^*}, \kappa_S^{(s)}),$$
$$S_{r,t^*} \sim N(S_{L,t^*}, \kappa_S^{(L)}),$$

$$S_{c,t^*} \sim N(S_{D,t^*}, \kappa_S^{(D)}), \quad c \in D,$$

such that the (logit-transformed) P_{c,t^*}'s are distributed around a sub-regional mean $S_{s,t^*[c]}$ ($s[c]$ refers to the subregion of country c), and the sub-regional means are distributed around a regional mean $S_{r,t^*[s]}$ ($r[s]$ is the region of sub-region s). The variances on the country, sub-regional and regional level are given by $\kappa_S^{(c)}$, $\kappa_S^{(s)}$ and $\kappa_S^{(r)}$ respectively.

2.4 The data model

Let $y_{i,m}$ denote the proportion of women in category m for observations $i = 1, \ldots, I$ where m refers to 1) women using a modern method, 2) women using a traditional method, 3) women with unmet need and 4) women without need for contraceptive methods (m = 1, 2, 3, 4). The data model corresponds to assuming a multivariate normal distribution for the log-ratios of all-but-one

of the categories in a compositional vector with respect to a left-out category. The justification for this data model is outlined in Alkema et al., 2013.

$$\begin{bmatrix} \log\left(\frac{y_{i,1}}{y_{i,3+4}}\right) \\ \log\left(\frac{y_{i,2}}{y_{i,3+4}}\right) \end{bmatrix} \sim N\left(\begin{bmatrix} \log\left(\frac{q_{i,1}}{q_{i,3+4}}\right) \\ \log\left(\frac{q_{i,2}}{q_{i,3+4}}\right) \end{bmatrix}, \boldsymbol{\Sigma}_i\right), \tag{10}$$

$$\log\left(\frac{y_{i,3}}{y_{i,4}}\right) \sim N\left(\log\left(\frac{q_{i,1}}{q_{i,4}}\right), \sigma^2_{i,3}\right), \tag{11}$$

where $q_{i,m}$ is the perturbed and bias-adjusted proportion of women in category m for observation i. Methods for applying perturbations and bias-adjustments are explained in detail in Alkema et al., 2013. Σ_i is the variance covariance matrix for the bivariate normal distribution of the log ratios.

For observation with outcomes on total contraceptive prevalence only (traditional + modern), the data model for these observations is given by:

$$log\Big(\frac{y_{i,1+2}}{1-y_{i,1+2}}\Big) \sim N\Big(log\Big(\frac{q_{i,1+2}}{1-q_{i,1+2}}\Big), \sigma_T^2\Big). \tag{12}$$

2.5 Survey specific standard errors

Calculating sampling errors Sampling errors for the prevalence ratios were calculated from microdata using a standard approach based on a Taylor approximation [8, 9]. For example, for the ratio of modern use to the no use category, y_1/y_{3+4} (leaving out the survey-specific indicator to simplify notation), the sampling variance is given by:

$$\begin{aligned} \text{Samp. var}(y_1/y_{3+4}) &= \frac{1}{t^2_{3+4}} \sum_{h=1}^{H} \frac{n_h}{n_h-1} \sum_{i=1}^{n_h} \left(x_{h,i} - \frac{x_h}{n_h}\right)^2, \\ x_{h,i} &= t_{1,h,i} - y_1/y_{3+4} \cdot t_{3+4,h,i}, \\ x_h &= t_{1,h} - y_1/y_{3+4} \cdot t_{3+4,h}, \end{aligned}$$

where H is the number of strata in the survey, n_h the number of clusters in stratum h, and $t_{g,\cdot}$ refers to the total number of women in category g in the survey, stratum or cluster, taking into account survey weights:

$$\begin{aligned} t_{g,h,i} &= \sum_j w_{h,i,j} \cdot u_{g,h,i,j}, \\ t_{g,h} &= \sum_{i=1}^{n_h} t_{g,h,i}, \\ t_g &= \sum_{h=1}^{H} t_{g,h}, \end{aligned}$$

where $u_{g,h,i,j} = 1$ if the j-th woman in cluster i in stratum g is in category k, $u_{k,h,i,j} = 0$ otherwise, and $w_{h,i,j}$ is the survey weight for that woman.

Sampling errors for the log-ratios are calculated by the Delta method [10]. For example, for $\log(y_1/y_{3+4})$:

$$\text{Samp. var}\big(log(y_1/y_{3+4})\big) = \frac{\text{Samp. var}(y_1/y_{3+4})}{(y_1/y_{3+4})^2}.$$

Imputing sampling errors We have two scenarios where we need to impute sampling errors:
1. Countries that have no observations with known sampling errors
2. Countries that observations with both known and unknown sampling errors.

For scenario 1 the imputation is carried out by calculating the maximum of known sampling errors across all other countries and setting the unknown sampling errors equal to the median of these maximums. For scenario 2 we impute the sampling errors by setting them equal to the maximum of the known sampling errors in that country.

Incorporating survey specific sampling errors

$$\Sigma_i = \begin{bmatrix} \sigma^2_{i,1} & \rho_i \sigma_{i,1}\sigma_{i,2} \\ \rho_i \sigma_{i,1}\sigma_{i,2} & \sigma^2_{i,2} \end{bmatrix}$$

where $\sigma^2_{i,k}$ are observation specific variances of the log ratios 1(traditional) and 2(modern) and 3(unmet) and ρ_i is the correlation between the log ratios 1 and 2 for observation i. The source specific variances combine known or imputed observation specific sampling errors for the log ratios ($\delta_{i,k}$) with an estimated source specific non-sampling error for the log ratios ($\tau_{S[i],k}$), where $S[i]$ is the survey source associated with observation i. $r_{S[i]}$ is the source specific correlation estimated for observation i.

$$\begin{aligned} \sigma^2_{i,k} &= \delta^2_{i,k} + \tau^2_{S[i],k}, \quad \text{for } S = 1,2,3,4 \text{ and } k = 1,2, \\ \sigma^2_{i,3} &= \delta^2_{i,3} + \tau^2_{S[i],3}, \quad \text{for } S = 1,2, \\ \rho_i &= r_{S[i]}, \qquad\quad \text{for } S = 1,2,3,4. \end{aligned}$$

2.6 Reference periods of observations

An extension to the Alkema et al., 2013 model [1] is to adjust the model so that the means in the data models given in equations 10, 11 and 12 refer to the entire reference period of the observation. Let s_i and e_i be the start calendar year and end calendar year of observation i. The unperturbed proportion $p_{i,m}$ for observation i and category m is given as follows:

$$p_{i,m} = \frac{1}{\sum_{t=s_i}^{e_i} v_{i,t}} \sum_{t=s_i}^{e_i} v_{i,t} p_{c[i],t,m},$$

where $p_{c[i],i_h,m}$ refers to the proportion in the country $c[i]$ (corresponding to the observation) and calendar year t, and $v_{i,t}$ refers to the proportion of the calendar year t associated with the i-th observation.

2.7 Prior distributions

Unless specified here, prior distributions for the model parameters are the same as those outlined in Alkema et al., 2013[1].

A vague prior distribution was used for the world-level mean of S_{c,t^*} such that,

$$S_{L,t^*} \sim N(-1, 10^2) \quad \text{and}$$
$$S_{D,t^*} \sim N(-1, 10^2).$$

Uninformative uniform prior distributions were used for the across country standard deviations $\sqrt{\kappa_{\cdot}^{(c)}}$ such that,

$$\sqrt{\kappa_{\cdot}^{(c)}} \sim U(0, 5).$$

Uniform prior distributions were used for the non sampling errors $\tau_{S,k}$ of the log ratios $k = 1$(traditional) and 2(modern) and 3(unmet) for each source breakdown:

$$\tau_{S,k} \sim U(0.01, 2), \quad \text{for } S = 1,2,3,4 \text{ and } k = 1,2,$$
$$\tau_{S,3} \sim U(0.01, 2), \quad \text{for } S = 1,2.$$

A flat uniform prior distribution was used for the correlation parameters r_S for each source, assuming that all values in the range of values that r_S can take are equally likely:

$$r_S \sim U(-1, 1).$$

2.8 Non-sampling error-variance parameters

The estimates for the standard deviations for observed log ratios, as well as the estimated correlation between the log ratios, are shown in Figure 5 (panels 1 and 2) for the four source categories (DHS, MICS, Other international survey programmes and National surveys). Errors tend to be smaller for the log-ratio of modern to no contraceptive prevalence compared to traditional to no contraceptive use. A comparison across sources shows that MICS tends to have higher error for both log-ratios as compared to the other 3 source categories, especially for the log-ratio of traditional to no contraceptive use. The correlation (panel 2) is small but positive for all source categories, suggesting that if a survey overestimates (underestimates) the ratio of modern to no contraceptive use, the ratio of traditional to no contraceptive use also tends to be overestimated (underestimated). Estimates of the error standard deviation of the log-ratio of unmet need for contraceptives to no need for observations from a DHS, and observations from other sources are shown in Figure 5 (panel 3). The non-sampling error is much smaller for observations from DHS compared to other sources and moreover, the error of other sources is very large, suggesting that these log-ratios are subject to substantial non-sampling errors.

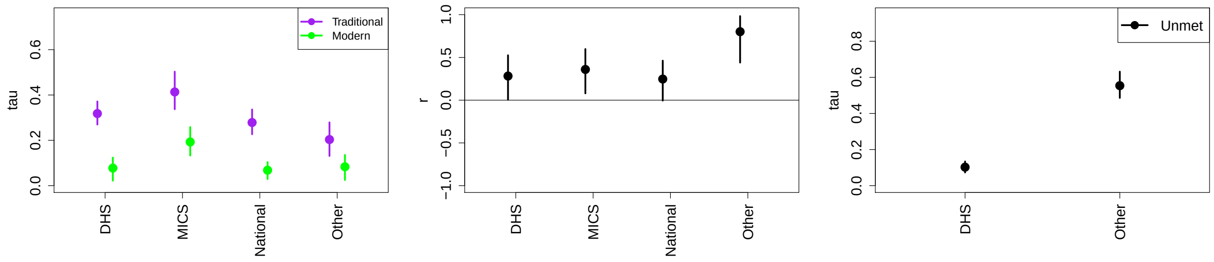

Figure 5: **Overview of estimates of source specific non-sampling errors of observed log ratios.** Estimated non-sampling errors (tau) and correlations (r) for the log ratios of traditional and modern contraceptive use to no use by source. Right graph: Estimated non-sampling errors (tau) for the log ratio of unmet need to no need for contraceptive methods, by source ('Other refers to any source other than DHS). Vertical lines display 95% uncertainty.

3 A country specific implementation of FPEM

FPEM produces estimates for all countries simultaneously and is thus thought of as a global model. The global FPEM uses a Bayesian hierarchical model set-up so that country-specific parameters are not solely estimated from country data but the global, regional and subregional experiences are also taken into account (section 2.3). Other parameters, such as the correlation coefficients and variances of the AR(1) processes, are global model parameters and are estimated using data from all countries.

Fitting the global FPEM is computationally intensive; it takes several days of parallel computing to obtain estimates. The computational burden makes it cumbersome for monitoring on a country level; updating a country's estimates based on one new data point takes a long time. A country specific implementation of FPEM provides an alternative to the global model estimation approach, which we refer to as the local FPEM. The key features of the local FPEM is that all non-country-specific parameters are fixed based on the results from the global FPEM rather than estimated. The local FPEM can be considered as a model with informative priors informed by the global FPEM (e.g., New et al., 2017 [11]). The flowchart in Figure 6 describes the relationship between the global FPEM to the local FPEM.

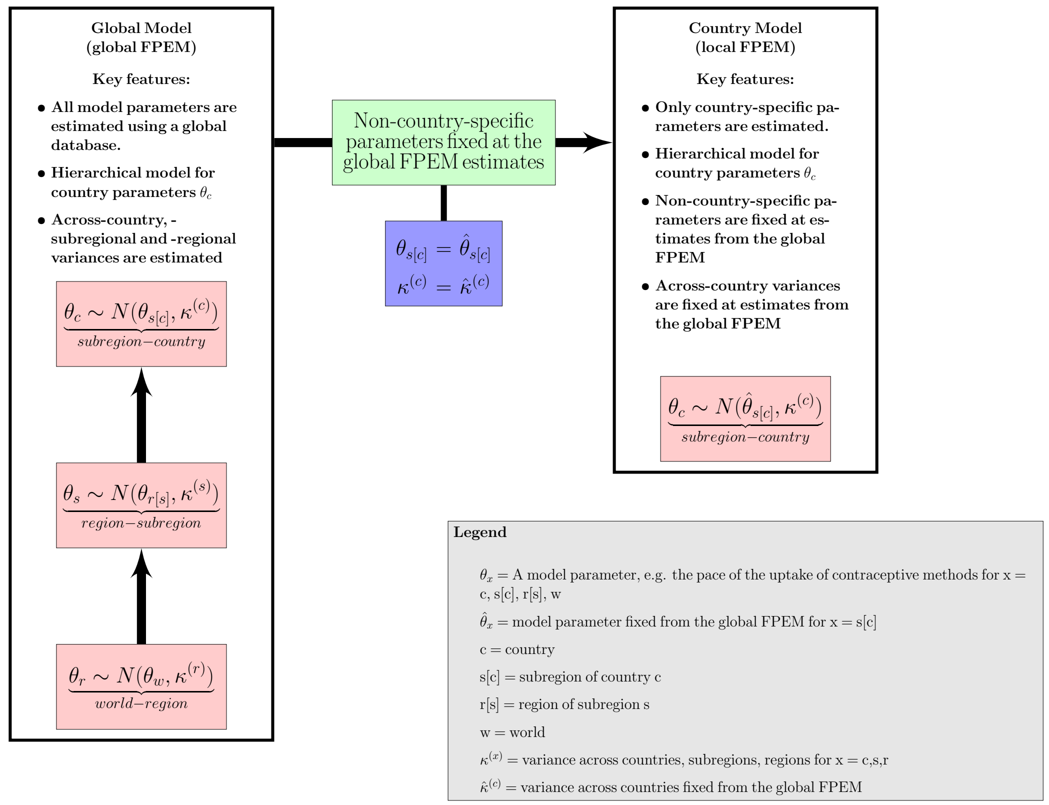

Figure 6: A flowchart to illustrate the steps required to transition from the global implementation of the family planning model (global FPEM) to the country specific implementation (local FPEM).

4 Utilizing Service Statistics Data

4.1 Data

SS data are routinely recorded at service delivery points in connection with family planning service delivery [5]. The data include information on the number of family planning service visits and the number of commodities distributed or sold to clients. Commodities and/or visits data are broken down by short term methods (STMs) and long-acting and permanent methods (LAPMs). STMs are those contraceptive methods that require multiple commodities for one year of coverage, such as oral contraceptive pills, condoms, and injectables. LAPMs are methods that offer multiple years of coverage per commodity distributed such as IUD, implant and sterilization.

4.2 Deriving Estimated Modern Use (EMU)

SS data are used to derive EMU in 2 steps:

1. For each method, the number of commodities distributed and/or the number of service

visits is converted into estimates of the number of users of that method.

2. In instances of underreporting into the countries' SS information systems (usually from the private sector), method specific adjustment factors are applied to make the numbers representative of the full market.

Conversion of SS data to users The calculation method for the conversion in step 1 differs between STMs and LAPMs. The number of STM users can be estimated by counting a 'user' as having full coverage for the entire year. For example, the distribution of 13 pill cycles is attributed to a single contraceptive pill user over the course of the year [12]. Hence, for STM m, the number of commodities distributed and/or the number of service visits in year t [$S_m(t)$] is converted into estimates of the number of contraceptive method users [$L_m(t)$] as follows:

$$L_m(t) \quad = \quad S_m(t)/\delta_m, \tag{13}$$

where STM-conversion parameter δ is the number of units needed for 1 year of coverage (i.e., 13 if $S_m(t)$ represents the number of pill cycles distributed) or the number of visits needed for 1 year of coverage if $S_m(t)$ refers to visit data. The values used for conversions of STMs are given in Table 2.

For LAPMs, estimating the number of users is more comprehensive than for STMs because long acting methods offer multiple years of coverage. Therefore, to obtain the number of users in a given year t, LAPM clients from past years need to be taken into account by applying method specific continuation rates to commodities data for years prior to t. The conversion formula for a LAPM m with maximum continuation of d_m years is as follows:

$$L_m(t) \quad = \quad \sum_{j=1}^{d_m} \gamma_{m,j} \cdot S_m(t-(j-1)), \tag{14}$$

where as for STMs, $L_m(t)$ refers to the number of users of method m estimated for year t, $S_m(t)$ refers to the number of commodities distributed in year t, and the $\gamma_{m,j}$s refer to continuation rates corresponding to the proportion of users that are still using the method in the j-th year of use. Where commodities data S_t are not available up to $d_m - 1$ years before the year of interest t, the data are estimated through a linear extrapolation backward from the earliest year with available data. The maximum number of years of use for LAPMs are given in Table 2.

Accounting for underreporting Once SS data have been converted into the corresponding number of method users, we have a representation of the number of users from the sector (private/public) that reported into the countries SS information systems. In instances of underreporting (usually from the private sector) method specific adjustment factors are applied to make the numbers representative of the full market. The adjusted number of users can then be summed across each method and divided by population numbers U(t) to provide EMU:

$$EMU(t) = \frac{\sum_m L_m(t)/\rho_m(t)}{U(t)}, \tag{15}$$

where $\rho_m(t)$ is the method-and-year specific proportion of users captured in the SS information systems. The current derivation for EMU does not quantify any uncertainty associated with this measure.

Method	Commodity conversion parameters	Visit conversion parameters
Condom	120 per user per year	3 visits per year of use
Injectable	2-13 per user per year	2-13 visits per year of use
Pill	15 per user per year	3 visits per year of use
Lactational Amenorrhea Method	4 per user per year	-
Vaginal Barrier	1 per user per year	1 visit per year of use
Spermicides	120 per user per year	3 visits per year of use
Emergency contraception	20 per user per year	20 visits per year of use
Sterilization	10 years of protection	10 years of protection
Implant	maximum 5 years of protection	maximum 5 years of protection
IUD	maximum 10 years of protection	maximum 10 years of protection

Table 2: Conversion parameters for short-acting methods and the maximum years of continuation for long acting and permanent methods.

4.3 Using EMU data in FPET

For modeling purposes, SS data are only used to provide information on the trend in mCPR since the last available survey and certain criteria need to be met if SS data is to be deemed reliable enough for trend estimation. The criteria are: 1. consistent levels of reporting of SS data over time; 2. at least 3 years of consistent data with at least one year overlapping with a survey; 3. At least one year of SS data reported *after* the most recent survey to ensure that the bias associated with the SS data can be estimated.

To estimate the SS bias two consecutive model runs are carried out. In the first run, only survey data that provide estimates of mCPR are used. The results of this run are used to estimate the relative bias of the service statistic EMU data. To estimate the bias for country c, we select the service statistics EMU observation in year t^x, the year closest or equal to the most recent survey year. We then assume that the observed EMU proportion in country c and year t^x is a perturbed version of the true mCPR in the same country and year, as follows:

$$z_{c,t^x} = \frac{p_{c,t^x,2} \cdot \hat{\chi}_c}{1 - p_{c,t^x,2} + p_{c,t^x,2} \cdot \hat{\chi}_c} \tag{16}$$

where z_{c,t^x} denotes the EMU of women in country c at time t^x, $p_{c,t^x,2}$ denotes the true mCPR in the same country and year and $\hat{\chi}_c$ denotes the estimated bias multiplier in country c. This set-up ensures that for a given multiplier, that service statistic EMU will always be restricted to be between 0 and 100%. For $\hat{\chi}_c > 1$, the true mCPR is smaller than z_{c,t^x} , for $\hat{\chi}_c < 1$ the true mCPR is

greater than z_{c,t^x}.

The bias multiplier for country c can be calculated by rewriting equation 16:

$$\hat{\chi}_c = \frac{z_{c,t^x} \cdot p_{c,t[t^x],2} - z_{c,t^x}}{z_{c,t^x} \cdot p_{c,t^x,2} - p_{c,t^x,2}}, \tag{17}$$

the calculation in equation 17 is based on the service statistic EMU in country c and year t^x and the estimated true mCPR in country c and year t^x obtained from the initial run (using the median model based estimate).

In the second run, EMU observations $z_{c,t[j]}$ with observation years $t[j] > t^x$ are included in the model to inform the trend of the true contraceptive prevalence as follows:

$$z_{c,t[j]} \sim N(p'_{c,t[j],2}, \epsilon^2), \quad \text{for } t[j] > t^x \tag{18}$$

$$p'_{c,t[j],2} = \frac{p_{c,t[j],2} \cdot \hat{\chi}_c}{1 - p_{c,t[j],2} + p_{c,t[j],2} \cdot \hat{\chi}_c}, \quad \text{for } t[j] > t^x \tag{19}$$

where $p'_{c,t[j],2}$ denotes the bias-adjusted EMU in country c and years $t[j] > t^x$ and ϵ denotes the standard error of the EMU observations, set to 2.5% (absolute, not relative to modern contraceptive prevalence).

5 Counter-factual projections and mCPR attainment probabilities

A reduced input dataset where all data after 2012 have been removed is used in FPET to produce estimates and projections of mCPR that reflect pre-FP2020 expectations (i.e., the expectations for mCPR before the introduction of the FP2020 initiative). We refer to these as counter-factual projections. For the FP2020 countries that have data after 2012, we compare the counter-factual projections for mCPR with current FPET estimates for mCPR in the most recent observation year.

Our comparison is made up of two components. The first is the difference between the counter-factual point estimate and the current point estimate for mCPR in the most recent observation year. The second is the probability of attaining the current estimate for mCPR in the most recent observation year based on what we knew in 2012 (i.e., based on the counter-factual projections).

To illustrate the calculation of the attainment probabilities we use Kenya as an example. The counter-factual projections shown in Figure 7. What underlies the counter-factual projections is a set of trajectories. We calculate the percentage of the trajectories that are greater than or equal to the mCPR estimate in the most recent observation year. This percentage provides the 2012 expectation for attaining the estimated mCPR or above and hence is referred to as an attainment probability. For example, we see that in Kenya the estimate for the most recent observation falls just outside of the 95% counter-factual uncertainty interval. This means that prior to the introduction of FP2020 there was a very low probability ($< 5\%$) that Kenya would reach the levels of mCPR currently estimated.

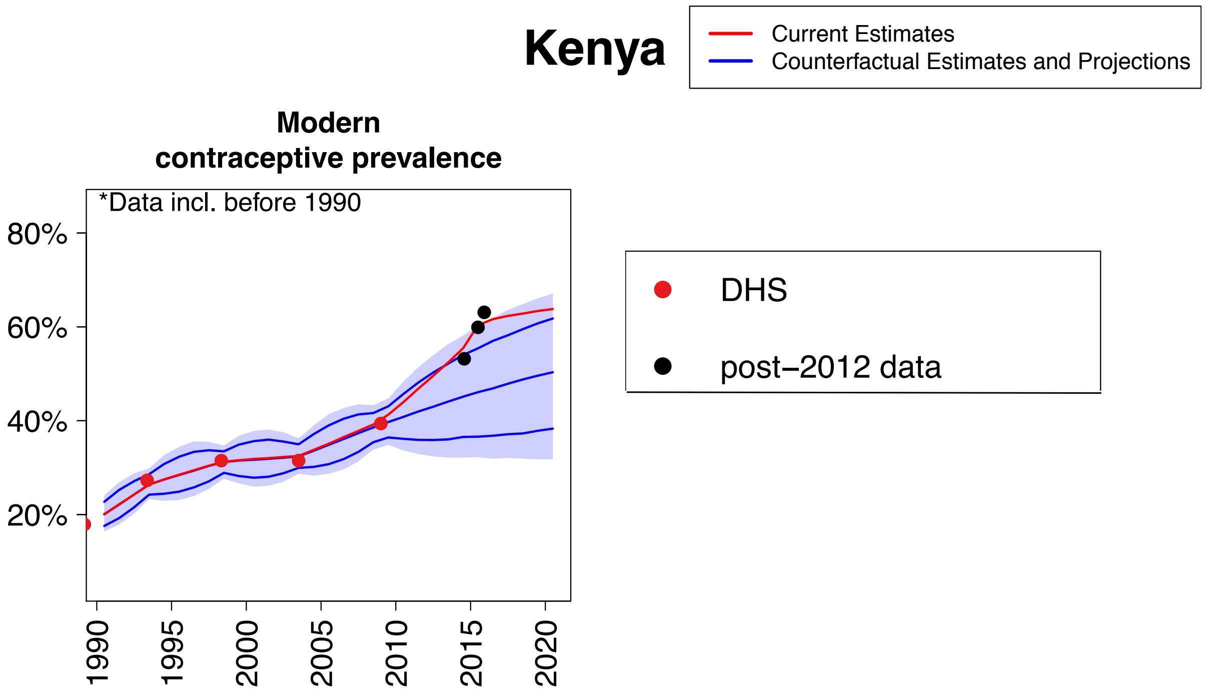

Figure 7: Estimates and counter-factual projections of mCPR in Kenya. The black lines represent the median fit and 80% uncertainty intervals and the blue shaded areas represent 95% uncertainty intervals for the counter-factual projections. The red line represents the current estimates of mCPR for Kenya. The red points are the available pre-2012 data which are surveys from the DHS. The black points are the data that were removed for the counter-factual model run and include one DHS survey and two PMA2020 surveys.

6 References

References

[1] Leontine Alkema, Vladimira Kantorova, Clare Menozzi, and Ann Biddlecom. National, regional, and global rates and trends in contraceptive prevalence and unmet need for family planning between 1990 and 2015: a systematic and comprehensive analysis. *The Lancet*, (9878):1642–1652, 2013.

[2] United Nations. Standard country or area codes for statistical use (m49), 2017. URL `https://unstats.un.org/unsd/methodology/m49/`.

[3] Sarah E.K. Bradley, Trevor N. Croft, Joy D. Fishel, and Charles F. Westoff. Revising unmet need for family planning. DHS Analytical Studies 25, USAID, Maryland, USA, 2012.

[4] Department of Economic United Nations and Population Division Social Affairs. World contraceptive use 2017, 2017.

[5] Track20. Monitoring progress in family planning., 2017. URL `http://www.track20.org`.

[6] Department of Economic United Nations and Population Division Social Affairs. Estimates and projections of the number of women aged 15-49 who are married or in a union: 2016 revision., 2016.

[7] Department of Economic United Nations and Population Division Social Affairs. National, regional and global estimates and projections of the number of women aged 15-49 who are married or in a union, 1970-2030. Technical Report No. 2013/2, New York: United Nations, 2013.

[8] Sharon L. Lohr. *Sampling: Design and Analysis*. Brooks/Cole, second edition edition, 2010.

[9] MEASURE DHS. Guide to dhs statistics, September 2006. URL `https://dhsprogram.com/publications/publication-DHSG1-DHS-Questionnaires-and-Manuals.cmf`.

[10] George Casella and Roger L. Berger. *Statistical Inference*. Thomson Learning, second edition edition, 2002.

[11] JR New, N Cahill, J Stover, YP Gupta, and L Alkema. Levels and trends in contraceptive prevalence, unmet need, and demand for family planning for 29 states and union territories in india: a modelling study using the family planning estimation tool. *The Lancet Global Health*, 5(3):e350–e358, 2017.

[12] Michelle B Weinberger, Kenzo Fry, Tania Boler, and Kristen Hopkins. Estimating the contribution of a service delivery organisation to the national modern contraceptive prevalence rate: Marie Stopes International's Impact 2 model. *BMC Public Health*, 13(Suppl 2):S5–S5, 2013.

7 Supplementary Table

Country	mCPR (%) 2012	Unmet need for modern methods (%) 2012	Demand satisfied with modern methods (%) 2012	mCPR (%) 2017	Unmet need for modern methods (%) 2017	Demand satisfied with modern methods (%) 2017
FP2020 countries	44.3 (42.4-46.0)	22.3 (20.8-24.0)	66.5 (64.1-68.7)	45.7 (42.4-49.1)	21.6 (19.7-23.9)	67.9 (64.4-71.1)
Caribbean	31.2 (27.7-34.9)	38.8 (35.1-42.4)	44.6 (40.0-49.2)	35.4 (24.2-48.4)	35.5 (27.0-43.9)	49.8 (36.6-63.6)
Central America	69.6 (66.6-72.4)	15.7 (13.3-18.7)	81.6 (78.2-84.4)	70.4 (62.4-77.4)	15.1 (10.5-21.2)	82.4 (74.8-87.9)
Central Asia	53.7 (43.2-62.5)	16.4 (12.2-22.5)	76.6 (66.4-83.5)	55.7 (42.5-66.3)	16.0 (11.1-23.1)	77.7 (65.5-85.5)
Eastern Africa	32.2 (30.5-34.0)	28.0 (26.6-29.5)	53.5 (51.2-55.8)	39.5 (37.0-42.0)	24.7 (23.1-26.5)	61.5 (58.7-64.3)
Eastern Asia	62.1 (51.7-70.9)	17.6 (11.8-25.5)	77.9 (67.3-85.5)	63.2 (49.0-75.2)	16.9 (9.9-26.8)	78.9 (65.5-88.1)
Melanesia	26.9 (17.2-38.9)	32.9 (23.9-43.6)	44.9 (31.3-59.3)	28.9 (16.0-45.5)	31.8 (22.0-43.5)	47.4 (29.9-65.5)
Middle Africa	9.1 (7.6-10.8)	36.7 (33.2-40.4)	20.0 (16.6-23.5)	12.1 (9.0-16.3)	36.2 (31.0-41.8)	25.2 (19.3-32.1)
Northern Africa	45.8 (40.6-50.7)	17.5 (14.3-21.2)	72.4 (66.6-77.5)	47.1 (39.4-54.6)	17.4 (13.3-22.1)	72.9 (64.9-79.9)
South America	39.7 (27.0-52.2)	40.8 (30.0-53.4)	49.3 (34.0-63.1)	44.1 (26.7-60.4)	36.7 (23.2-53.5)	54.5 (33.5-71.9)
South-Eastern Asia	55.8 (54.1-57.5)	18.5 (17.1-20.0)	75.1 (73.2-77.0)	56.5 (51.6-61.3)	18.4 (15.4-21.7)	75.4 (70.7-79.7)
Southern Africa	53.4 (47.0-59.6)	22.1 (18.1-26.6)	70.7 (64.3-76.5)	60.9 (50.6-70.4)	17.8 (12.1-24.7)	77.4 (67.6-85.1)
Southern Asia	49.8 (46.5-52.9)	21.1 (18.5-24.2)	70.2 (66.2-73.7)	50.6 (44.4-56.8)	20.3 (17.0-24.4)	71.4 (65.2-76.5)
Western Africa	12.1 (11.2-13.1)	25.7 (24.0-27.6)	31.9 (29.7-34.2)	15.6 (13.8-17.9)	26.8 (24.2-29.8)	36.8 (33.1-40.9)
Western Asia	35.7 (29.7-42.0)	30.7 (25.6-36.7)	53.7 (45.6-61.4)	39.8 (30.4-49.7)	29.2 (22.5-37.1)	57.6 (46.2-68.2)
Africa	23.9 (22.9-25.0)	26.7 (25.7-27.8)	47.2 (45.5-48.9)	28.5 (26.8-30.2)	25.9 (24.5-27.5)	52.4 (49.9-54.7)
Asia	51.0 (48.5-53.4)	20.7 (18.7-23.0)	71.2 (68.2-73.8)	51.8 (47.3-56.5)	20.0 (17.5-23.1)	72.1 (67.8-76.0)
Latin America and the Caribbean	49.7 (45.9-53.3)	29.6 (26.3-33.3)	62.7 (58.1-66.8)	52.4 (45.7-59.1)	27.3 (22.3-32.9)	65.7 (58.7-72.3)
Oceania	26.9 (17.2-38.9)	32.9 (23.9-43.6)	44.9 (31.3-59.3)	28.9 (16.0-45.5)	31.8 (22.0-43.5)	47.4 (29.9-65.5)

Table 1: Columns 2 to 7 show the modern contraceptive prevalence rate (mCPR), unmet need for modern methods and demand satisfied with modern methods in 2012 and 2017 for the FP2020 countries, regions and subregions.

1

8 Supplementary Figures

Figure 7: Data and modeled rates and trends of modern contraceptive prevalence, unmet need for modern contraceptive methods and demand satisfied with modern contraceptive methods for 68 FP2020 countries, regional aggregates and an all-country aggregate. Circles represent survey observations; black lines represent the median fit and 80% uncertainty intervals; blue shaded areas represent 95% uncertainty intervals.

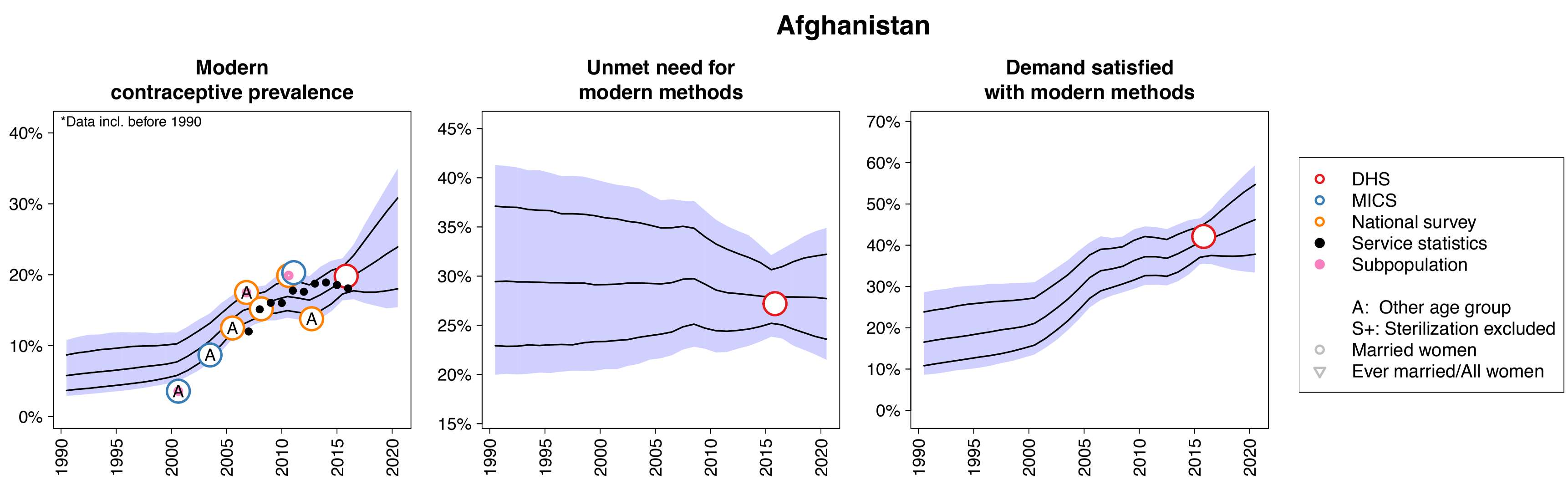
Afghanistan
Modern
contraceptive prevalence
*Data incl. before 1990
40%
30%
20%
10%
0%
1990
1995
2000
2005
2010
2015
2020
Unmet need for
modern methods
45%
40%
35%
30%
25%
20%
15%
Demand satisfied
with modern methods
70%
60%
50%
40%
30%
20%
10%
0%
DHS
MICS
National survey
Service statistics
Subpopulation
A: Other age group
S+: Sterilization excluded
Married women
Ever married/All women

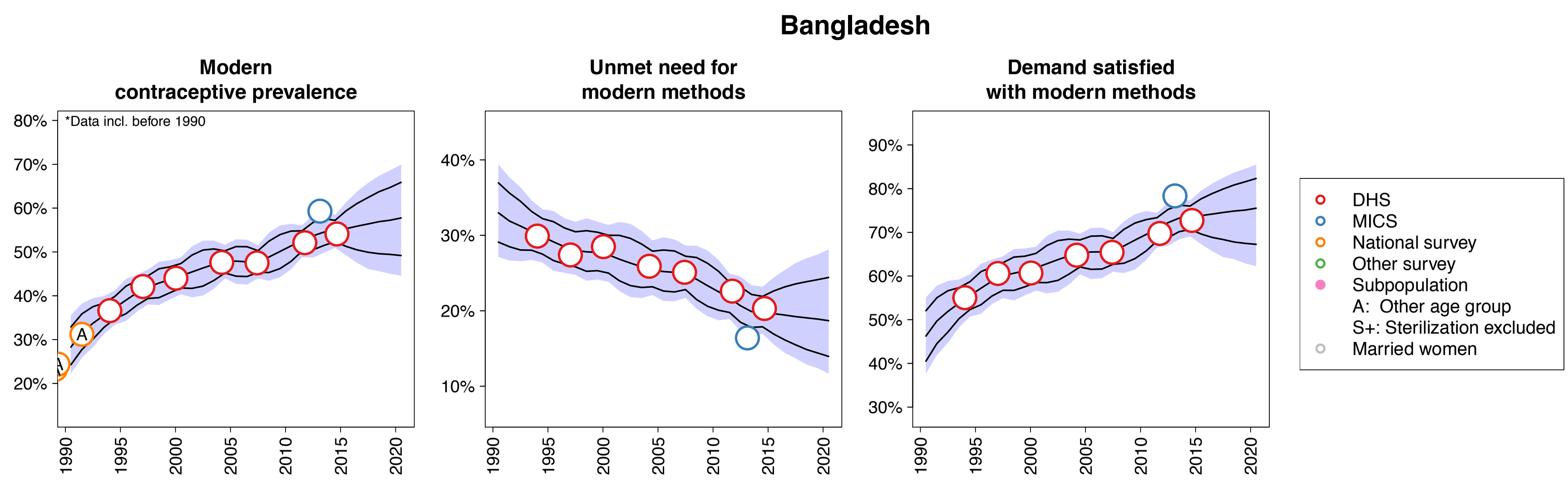
Bangladesh
Modern
contraceptive prevalence
*Data incl. before 1990
80%
70%
60%
50%
40%
30%
20%
1990
1995
2000
2005
2010
2015
2020
A
Unmet need for
modern methods
40%
30%
20%
10%
Demand satisfied
with modern methods
90%
80%
70%
60%
50%
40%
30%
DHS
MICS
National survey
Other survey
Subpopulation
A: Other age group
S+: Sterilization excluded
Married women

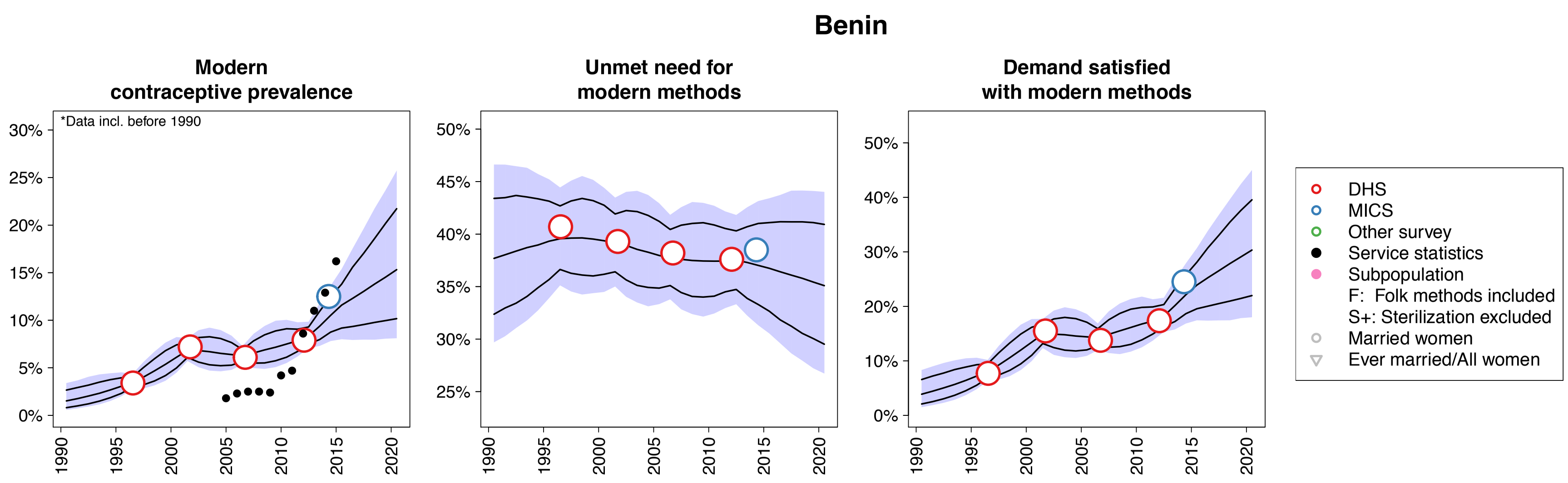
Benin
Modern
contraceptive prevalence
*Data incl. before 1990
30%
25%
20%
15%
10%
5%
0%
1990
1995
2000
2005
2010
2015
2020
Unmet need for
modern methods
50%
45%
40%
35%
30%
25%
Demand satisfied
with modern methods
50%
40%
30%
20%
10%
0%
DHS
MICS
Other survey
Service statistics
Subpopulation
F: Folk methods included
S+: Sterilization excluded
Married women
Ever married/All women

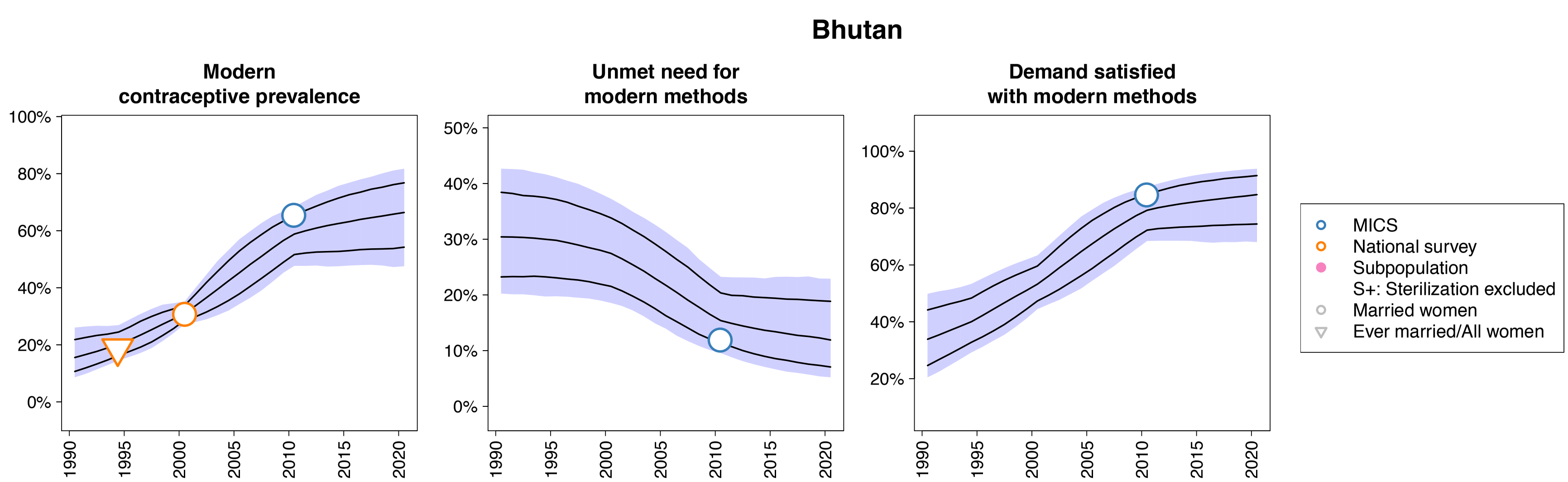

Bhutan
Modern contraceptive prevalence
Unmet need for modern methods
Demand satisfied with modern methods
100%
80%
60%
40%
20%
0%
50%
30%
10%
1990
1995
2000
2005
2010
2015
2020
MICS
National survey
Subpopulation
S+: Sterilization excluded
Married women
Ever married/All women

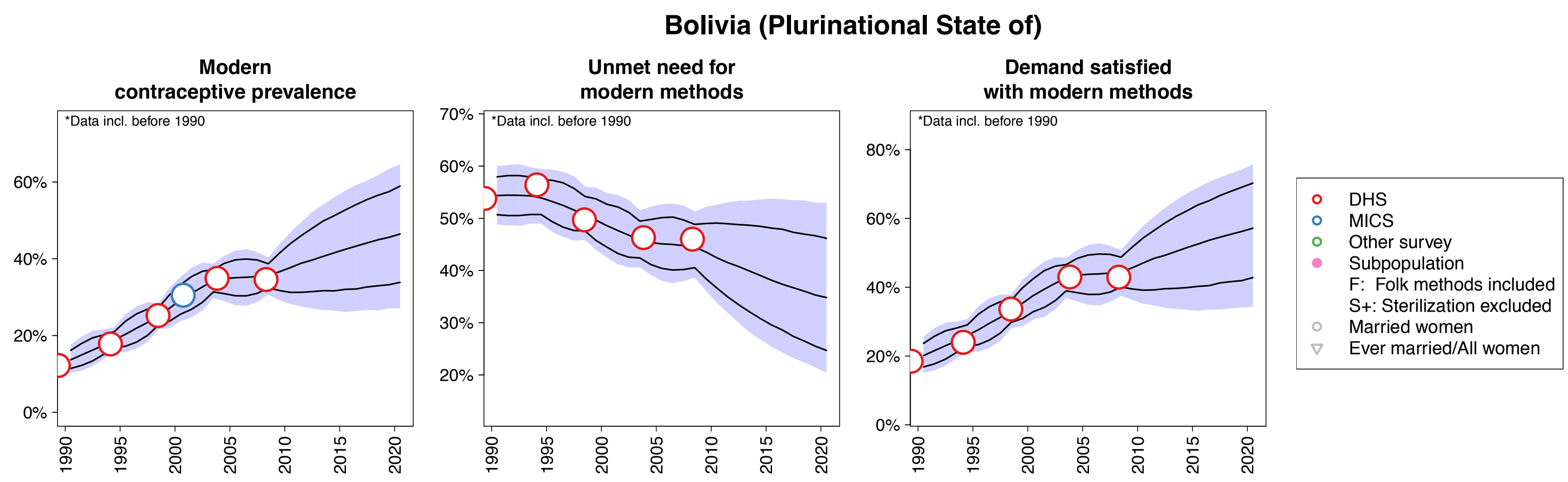
Bolivia (Plurinational State of)
Modern
contraceptive prevalence
Unmet need for
modern methods
Demand satisfied
with modern methods
*Data incl. before 1990
*Data incl. before 1990
*Data incl. before 1990
0%
20%
40%
60%
20%
30%
40%
50%
60%
70%
0%
20%
40%
60%
80%
1990
1995
2000
2005
2010
2015
2020
DHS
MICS
Other survey
Subpopulation
F: Folk methods included
S+: Sterilization excluded
Married women
Ever married/All women

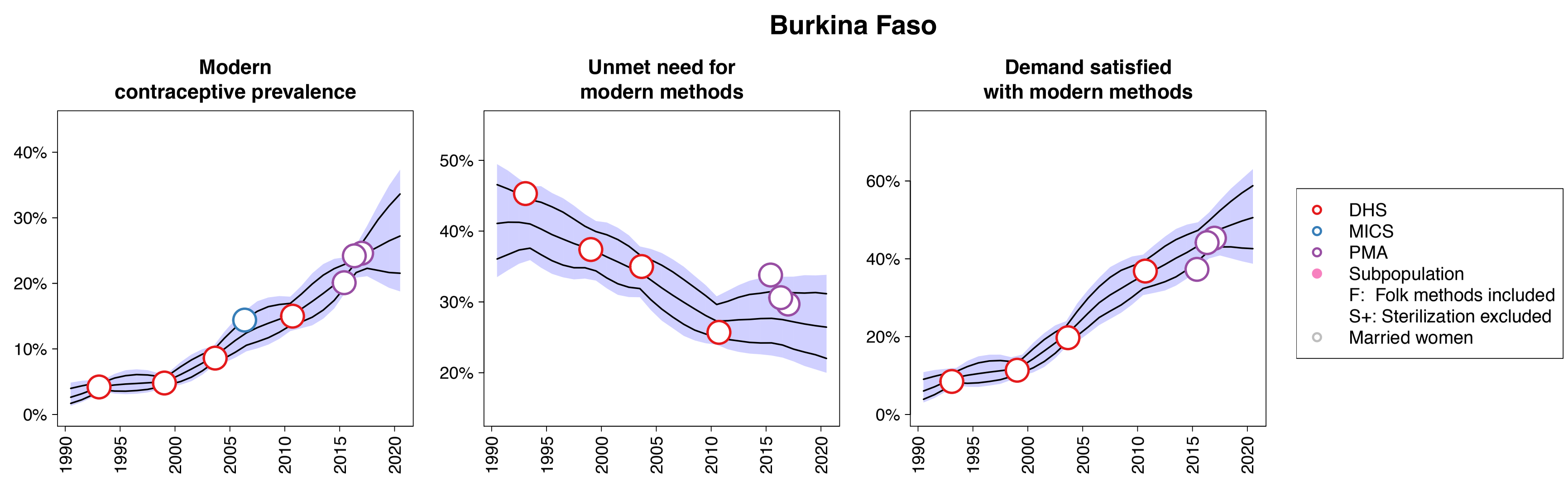
Burkina Faso
Modern
contraceptive prevalence
Unmet need for
modern methods
Demand satisfied
with modern methods
0%
10%
20%
30%
40%
50%
60%
1990
1995
2000
2005
2010
2015
2020
DHS
MICS
PMA
Subpopulation
F: Folk methods included
S+: Sterilization excluded
Married women

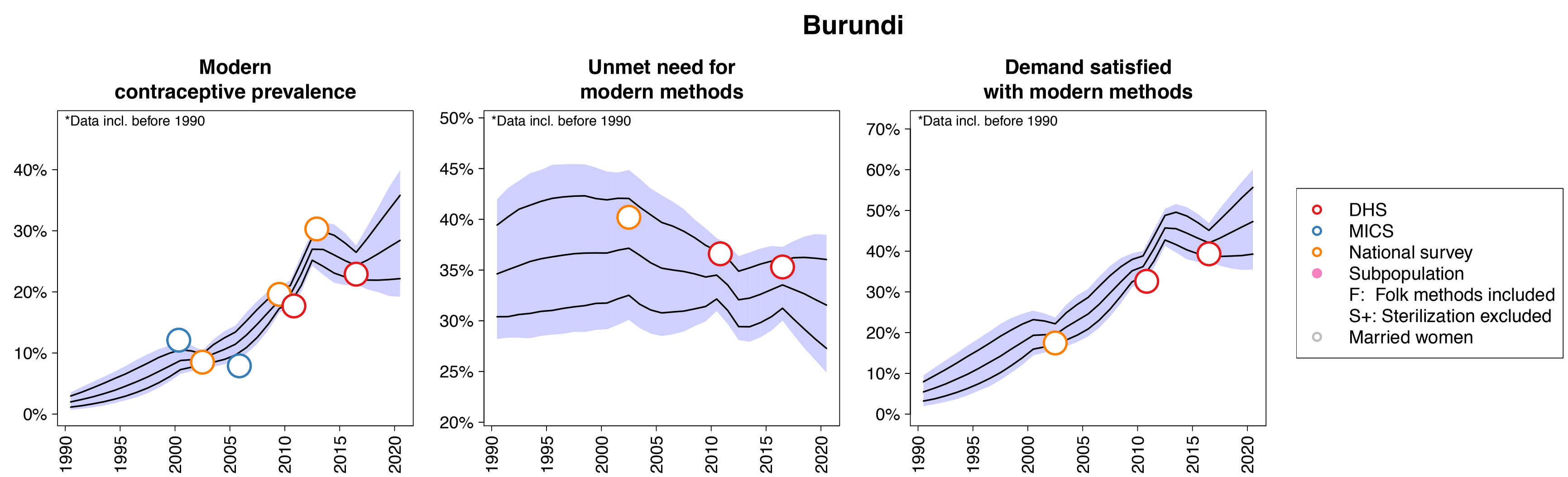
Burundi
Modern
contraceptive prevalence
*Data incl. before 1990
40%
30%
20%
10%
0%
1990
1995
2000
2005
2010
2015
2020
Unmet need for
modern methods
*Data incl. before 1990
50%
45%
40%
35%
30%
25%
20%
Demand satisfied
with modern methods
*Data incl. before 1990
70%
60%
50%
40%
30%
20%
10%
0%
DHS
MICS
National survey
Subpopulation
F: Folk methods included
S+: Sterilization excluded
Married women

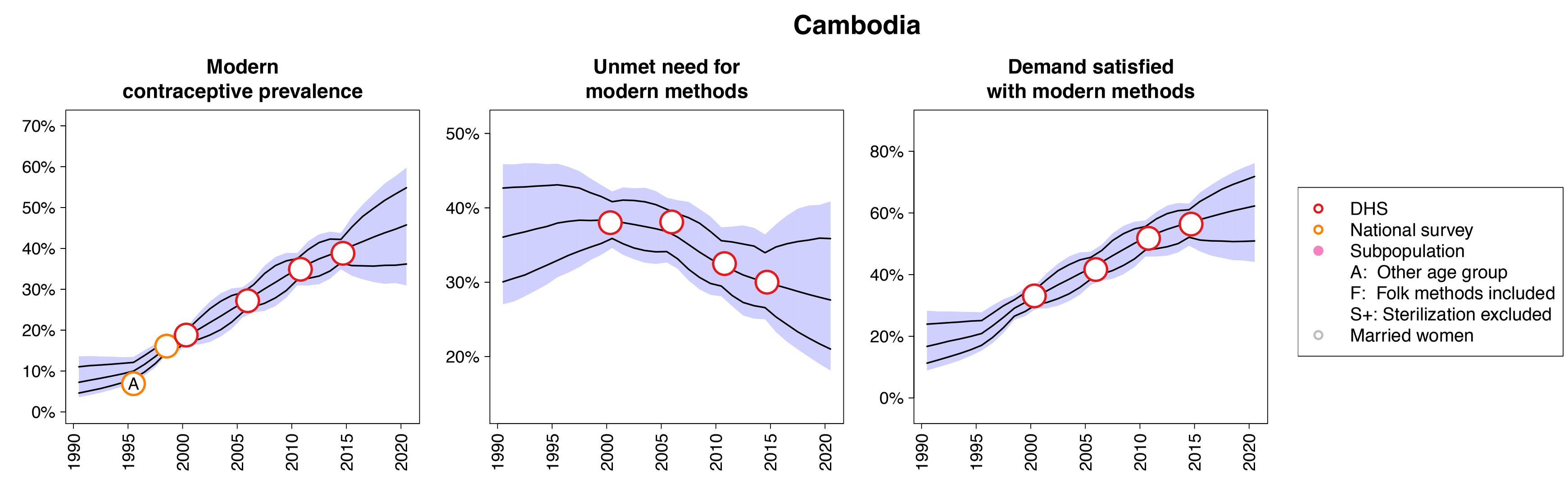

Cambodia
Modern contraceptive prevalence
Unmet need for modern methods
Demand satisfied with modern methods
70%
60%
50%
40%
30%
20%
10%
0%
80%
1990
1995
2000
2005
2010
2015
2020
A
DHS
National survey
Subpopulation
A: Other age group
F: Folk methods included
S+: Sterilization excluded
Married women

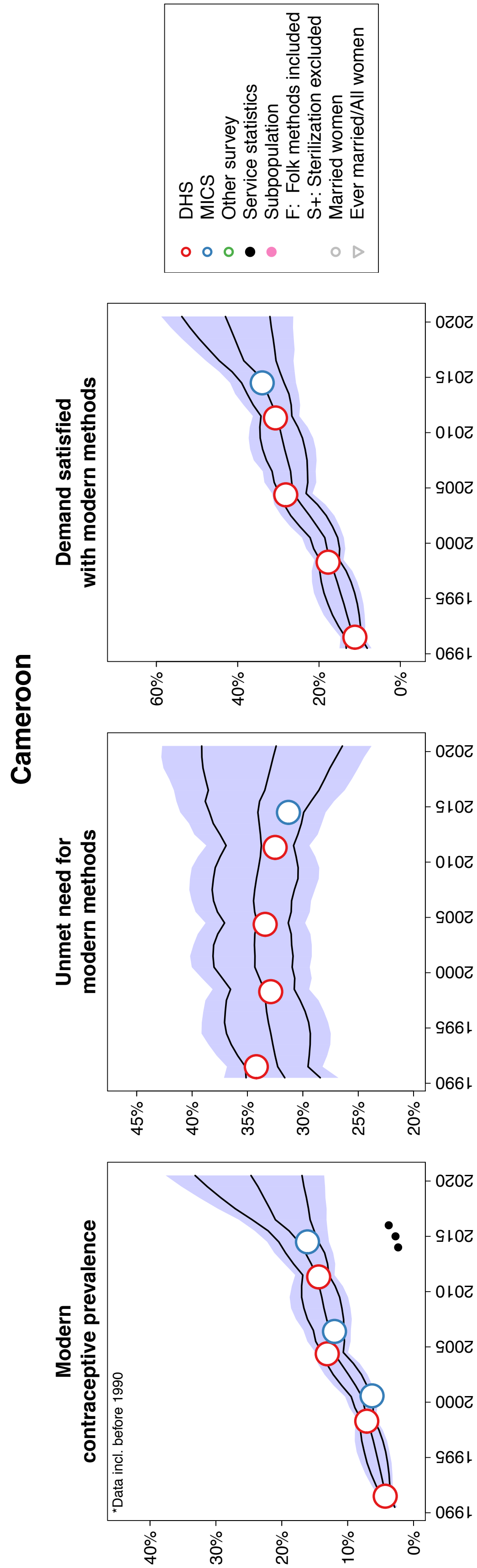

Cameroon
Modern contraceptive prevalence
*Data incl. before 1990
Unmet need for modern methods
Demand satisfied with modern methods
0%
10%
20%
30%
40%
25%
35%
45%
60%
1990
1995
2000
2005
2010
2015
2020
DHS
MICS
Other survey
Service statistics
Subpopulation
F: Folk methods included
S+: Sterilization excluded
Married women
Ever married/All women

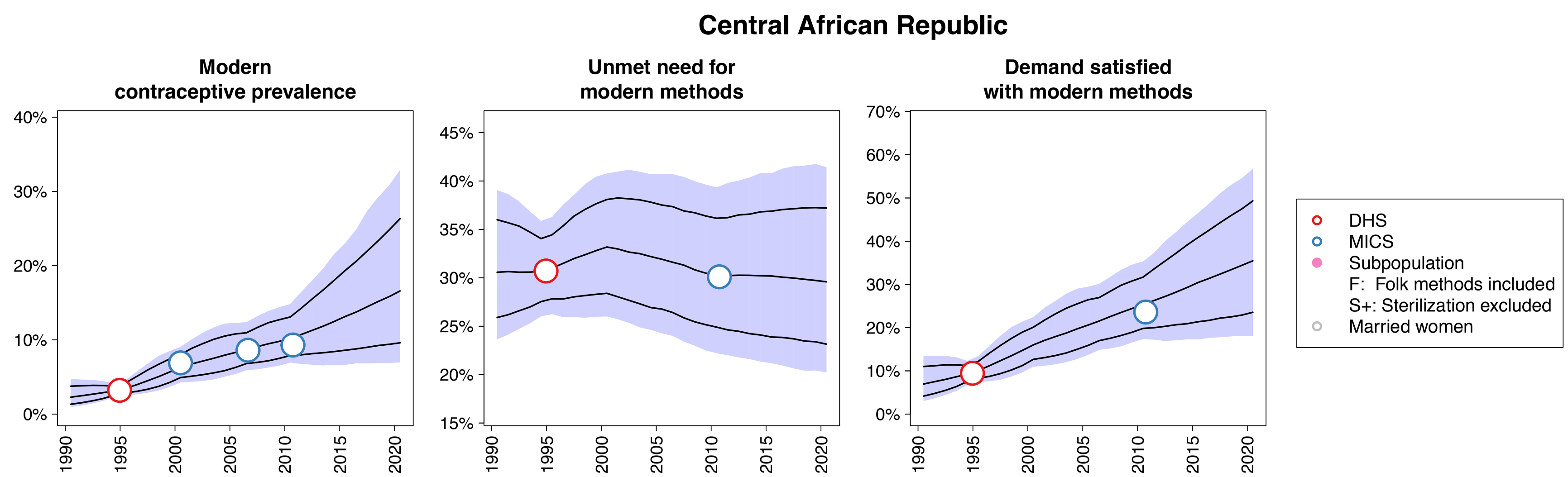
Central African Republic
Modern
contraceptive prevalence
Unmet need for
modern methods
Demand satisfied
with modern methods
40%
30%
20%
10%
0%
45%
35%
25%
15%
70%
60%
50%
1990
1995
2000
2005
2010
2015
2020
DHS
MICS
Subpopulation
F: Folk methods included
S+: Sterilization excluded
Married women

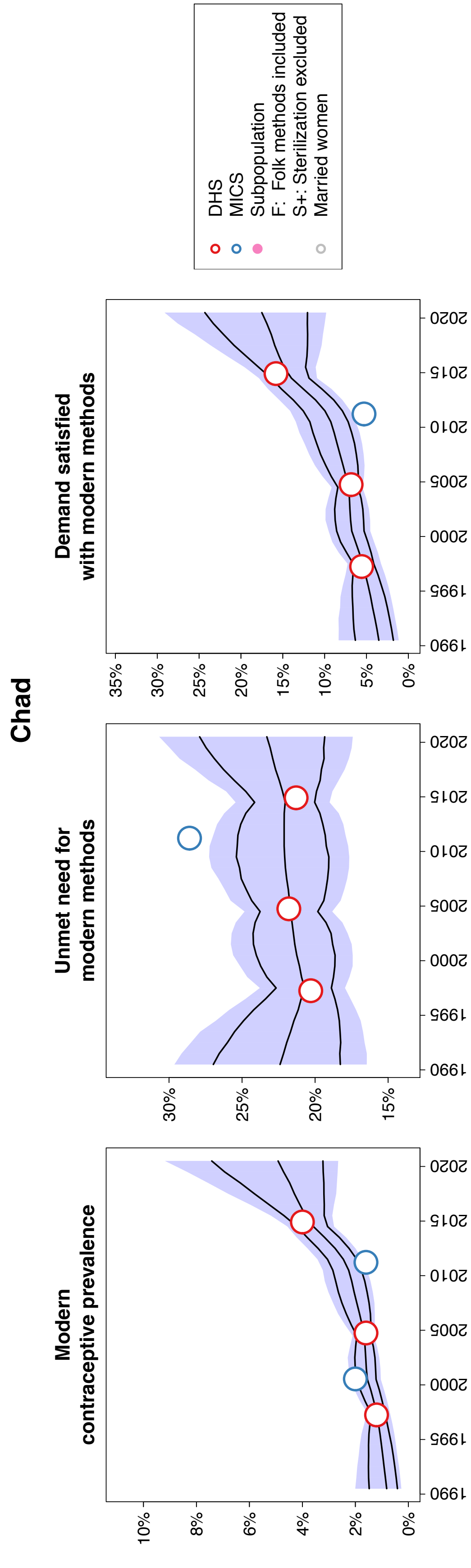

Chad
Modern contraceptive prevalence
Unmet need for modern methods
Demand satisfied with modern methods
DHS
MICS
Subpopulation
F: Folk methods included
S+: Sterilization excluded
Married women

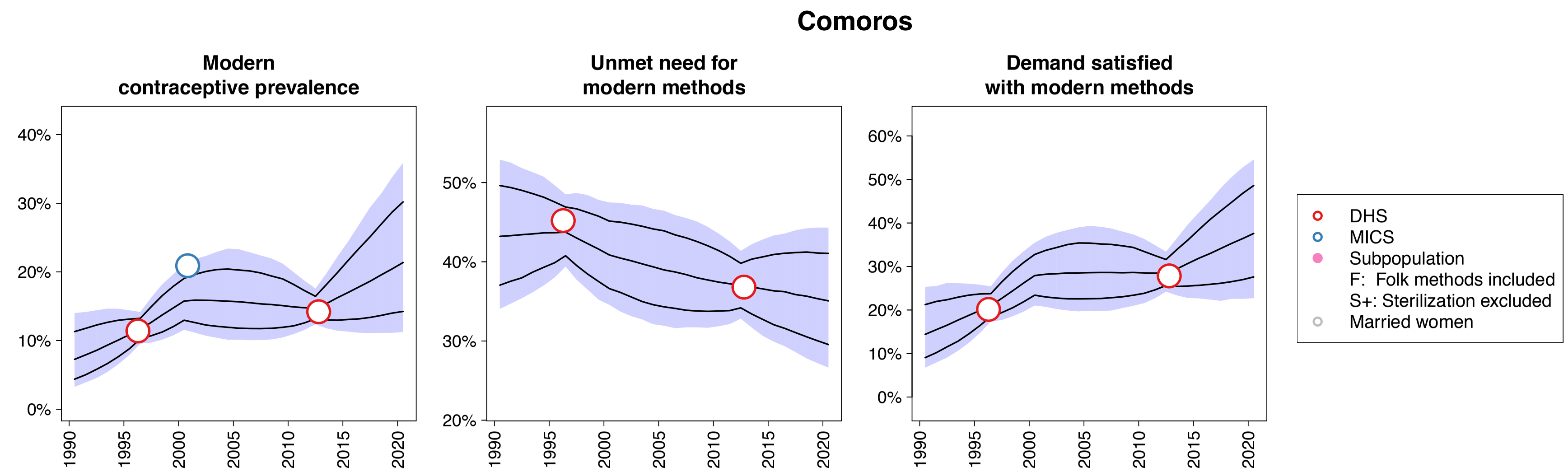
Comoros
Modern
contraceptive prevalence
Unmet need for
modern methods
Demand satisfied
with modern methods
0%
10%
20%
30%
40%
50%
60%
1990
1995
2000
2005
2010
2015
2020
DHS
MICS
Subpopulation
F: Folk methods included
S+: Sterilization excluded
Married women

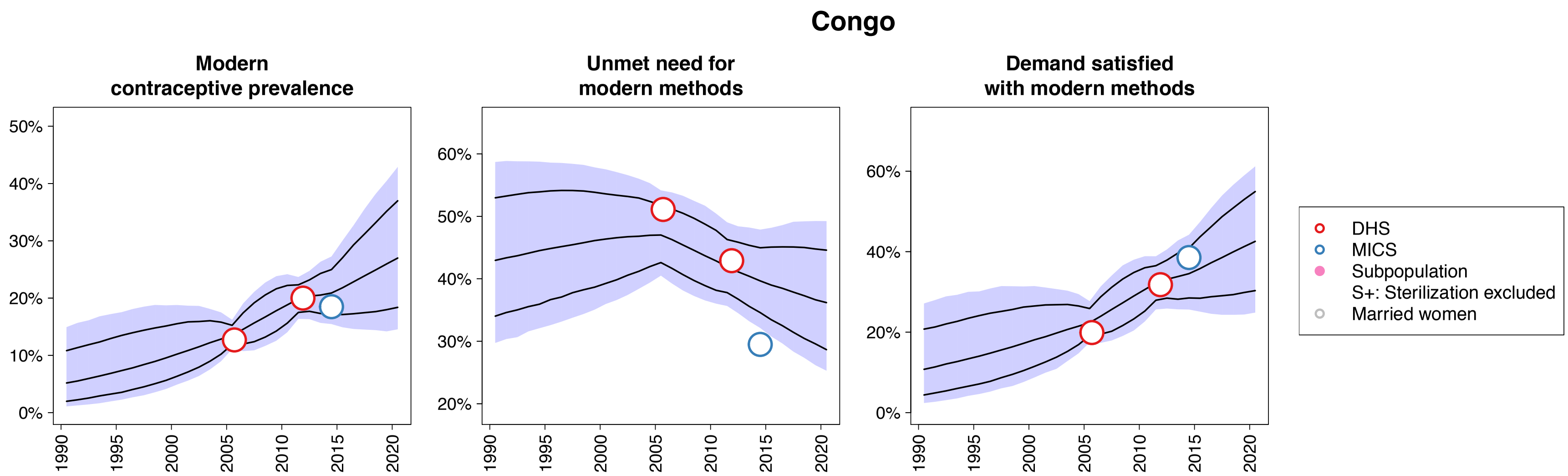
Congo
Modern contraceptive prevalence
Unmet need for modern methods
Demand satisfied with modern methods
0%
10%
20%
30%
40%
50%
60%
1990
1995
2000
2005
2010
2015
2020
DHS
MICS
Subpopulation
S+: Sterilization excluded
Married women

Cote d'Ivoire

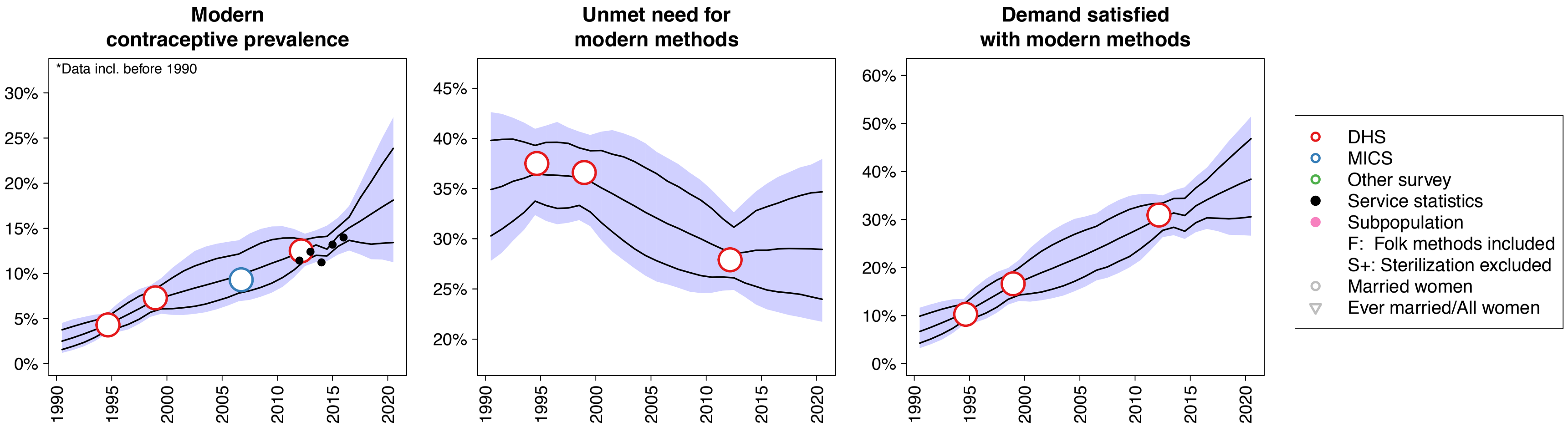

Democratic People's Republic of Korea

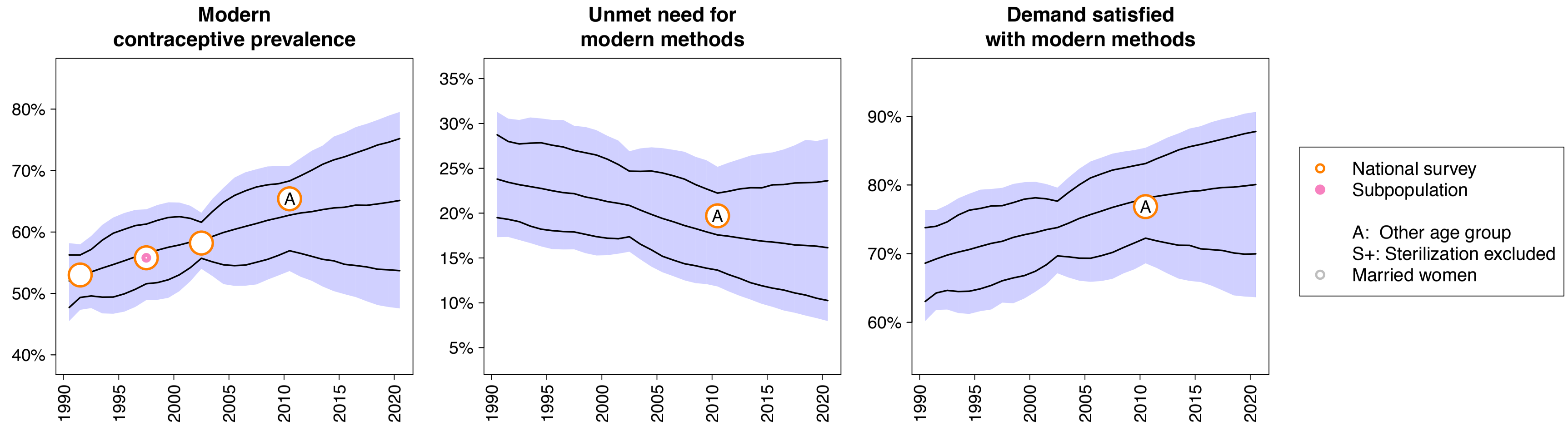

Democratic Republic of the Congo

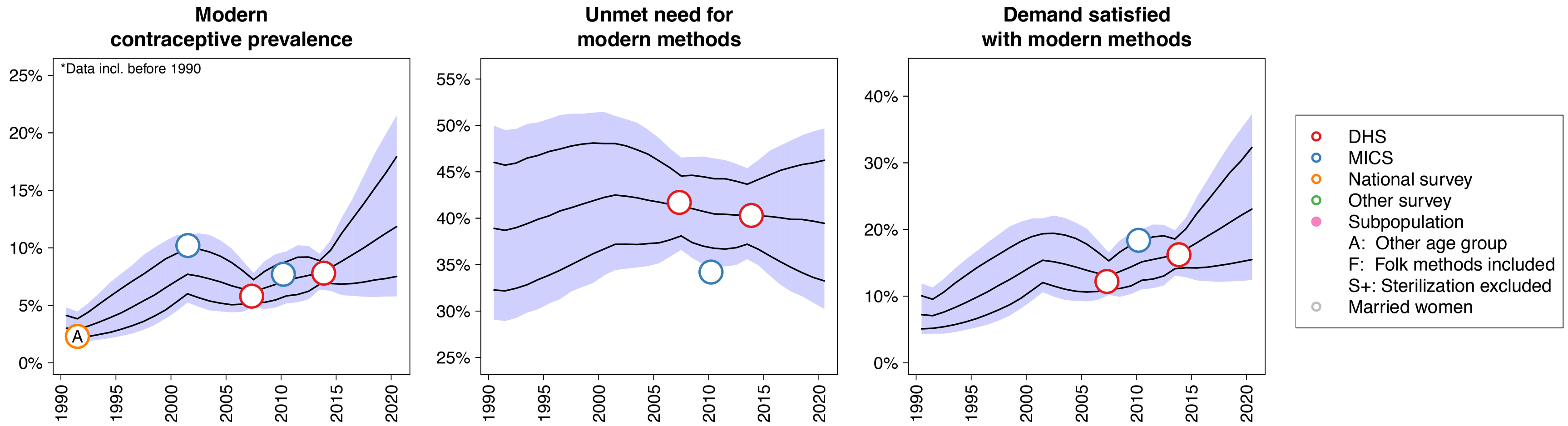

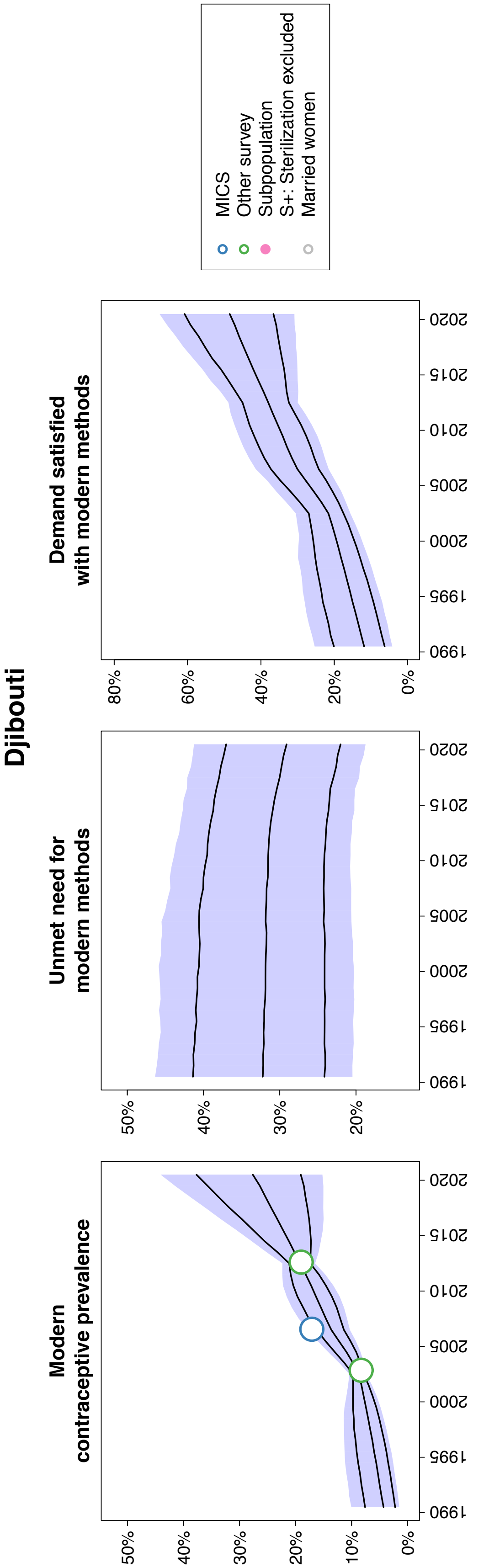
Djibouti
Modern
contraceptive prevalence
Unmet need for
modern methods
Demand satisfied
with modern methods
0%
10%
20%
30%
40%
50%
60%
80%
1990
1995
2000
2005
2010
2015
2020
MICS
Other survey
Subpopulation
S+: Sterilization excluded
Married women

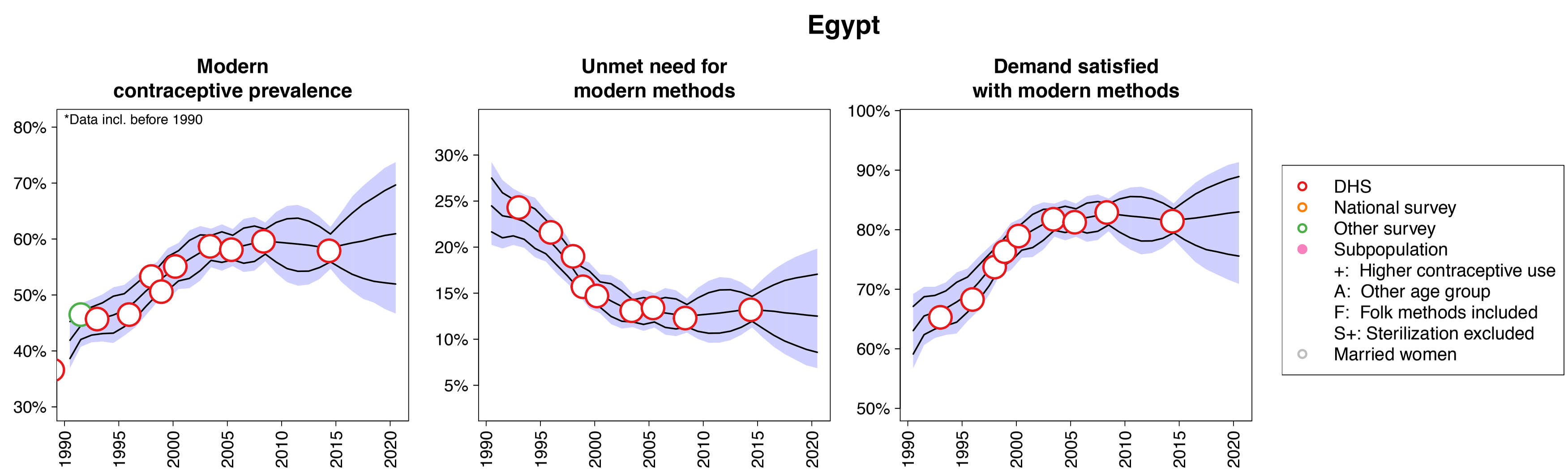
Egypt
Modern
contraceptive prevalence
*Data incl. before 1990
80%
70%
60%
50%
40%
30%
Unmet need for
modern methods
30%
25%
20%
15%
10%
5%
Demand satisfied
with modern methods
100%
90%
80%
70%
60%
50%
1990
1995
2000
2005
2010
2015
2020
DHS
National survey
Other survey
Subpopulation
+: Higher contraceptive use
A: Other age group
F: Folk methods included
S+: Sterilization excluded
Married women

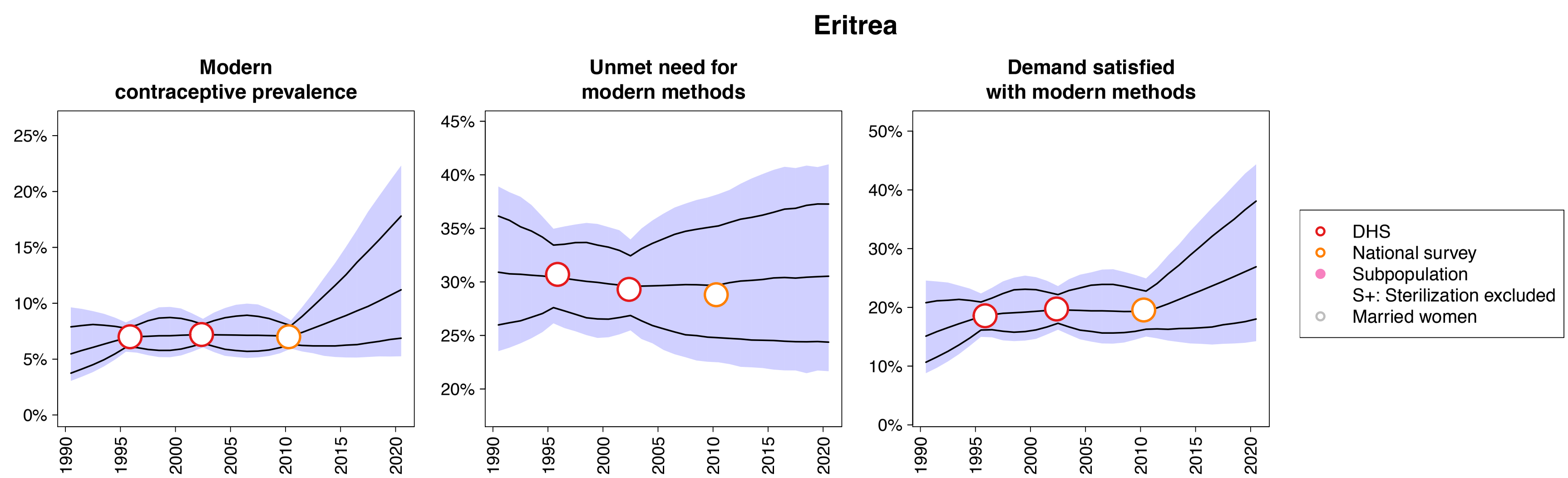
Eritrea
Modern contraceptive prevalence
Unmet need for modern methods
Demand satisfied with modern methods
DHS
National survey
Subpopulation
S+: Sterilization excluded
Married women

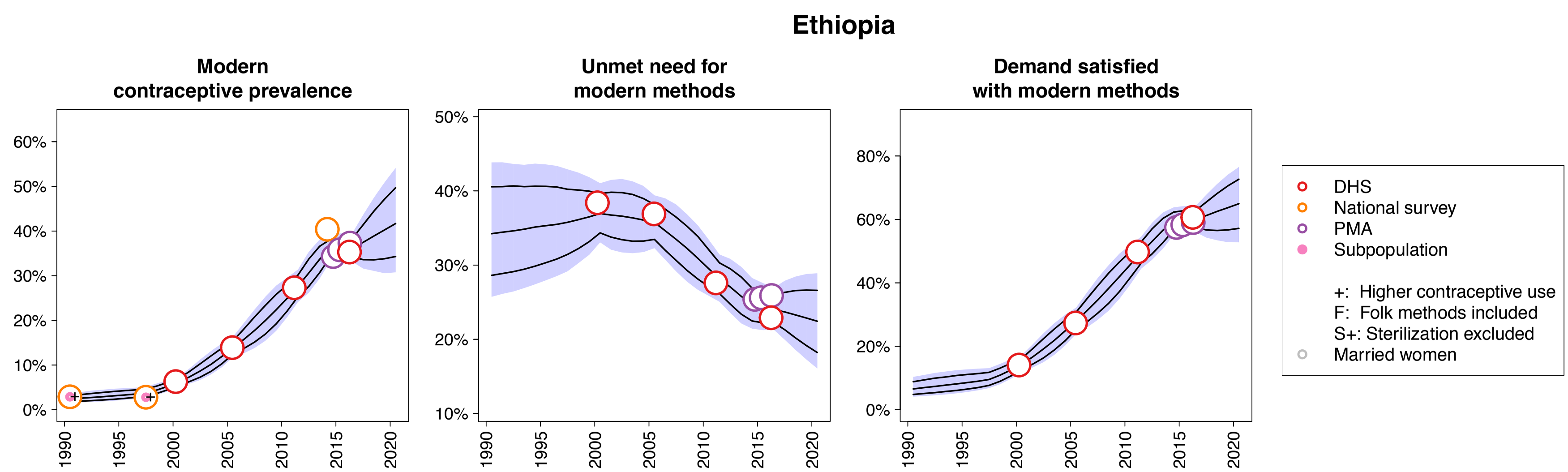
Ethiopia
Modern contraceptive prevalence
Unmet need for modern methods
Demand satisfied with modern methods
60%
50%
40%
30%
20%
10%
0%
80%
1990
1995
2000
2005
2010
2015
2020
DHS
National survey
PMA
Subpopulation
+: Higher contraceptive use
F: Folk methods included
S+: Sterilization excluded
Married women

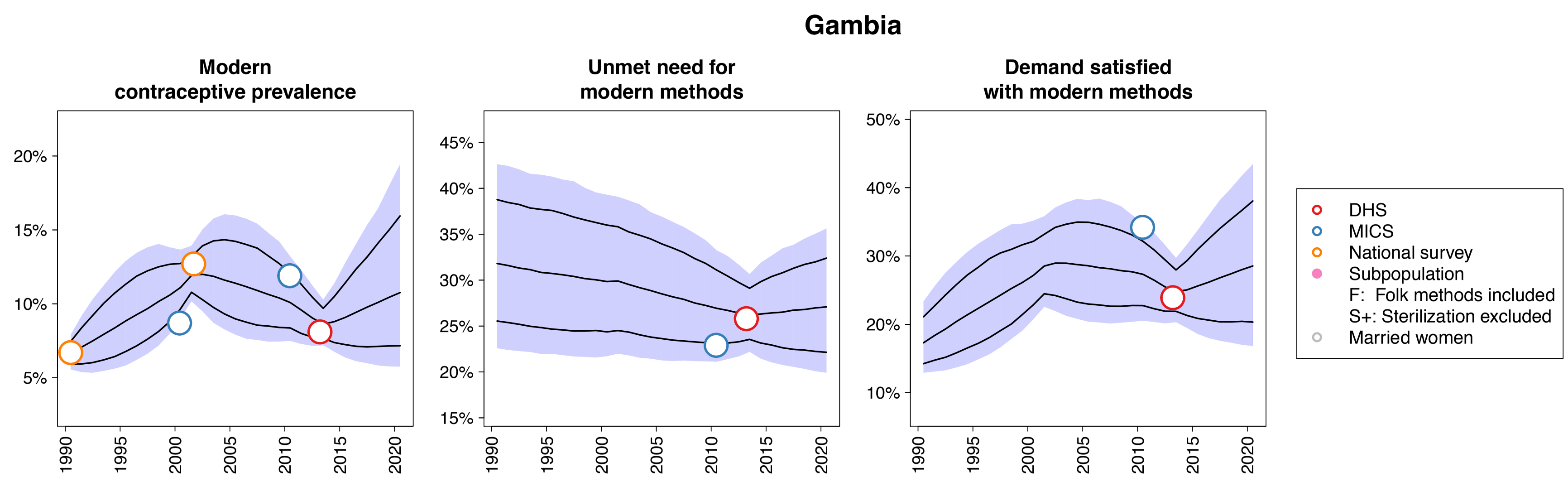
Gambia
Modern
contraceptive prevalence
Unmet need for
modern methods
Demand satisfied
with modern methods
20%
15%
10%
5%
45%
40%
35%
30%
25%
20%
15%
50%
40%
30%
20%
10%
1990
1995
2000
2005
2010
2015
2020
DHS
MICS
National survey
Subpopulation
F: Folk methods included
S+: Sterilization excluded
Married women

Ghana

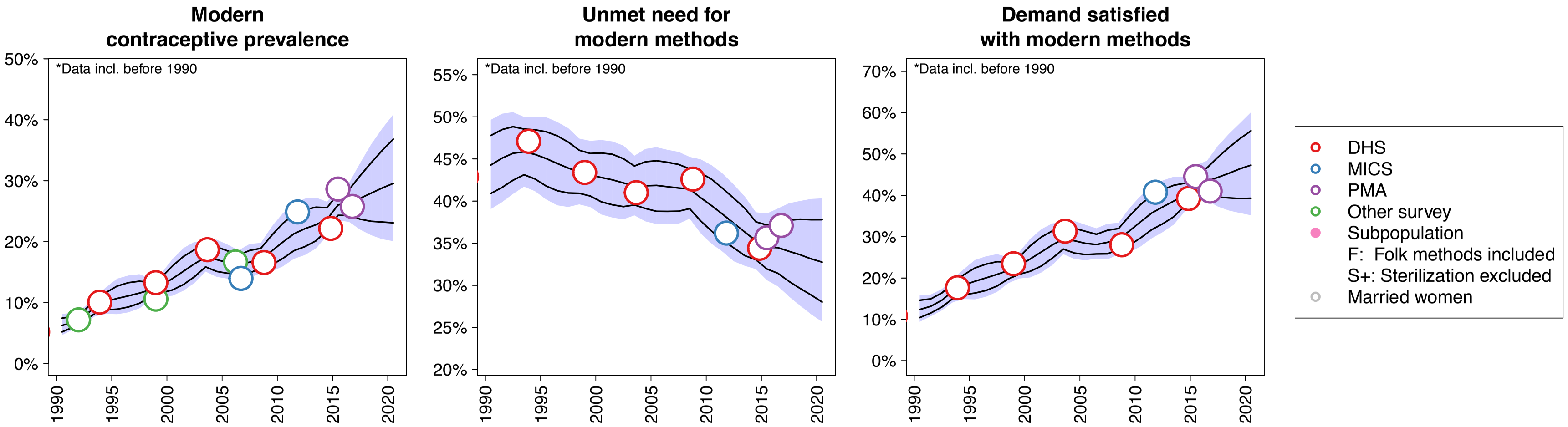
Modern
contraceptive prevalence
Unmet need for
modern methods
Demand satisfied
with modern methods
*Data incl. before 1990
50%
40%
30%
20%
10%
0%
55%
45%
35%
25%
70%
60%
1990
1995
2000
2005
2010
2015
2020
DHS
MICS
PMA
Other survey
Subpopulation
F: Folk methods included
S+: Sterilization excluded
Married women

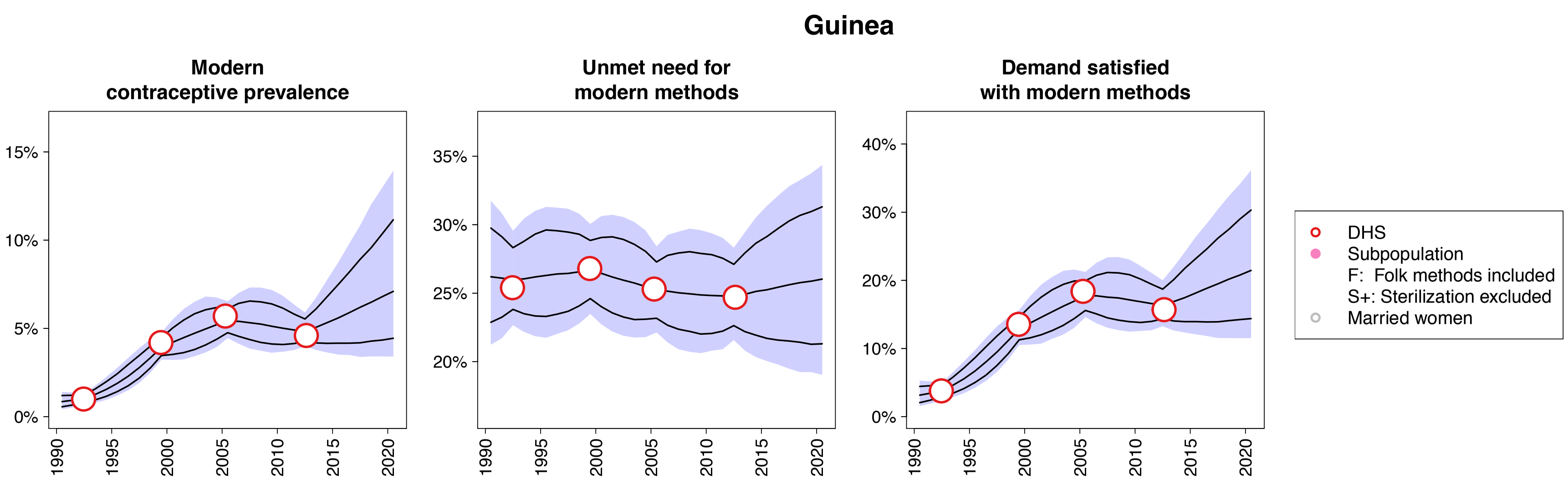
Guinea
Modern
contraceptive prevalence
Unmet need for
modern methods
Demand satisfied
with modern methods
15%
10%
5%
0%
35%
30%
25%
20%
40%
30%
20%
10%
0%
1990
1995
2000
2005
2010
2015
2020
DHS
Subpopulation
F: Folk methods included
S+: Sterilization excluded
Married women

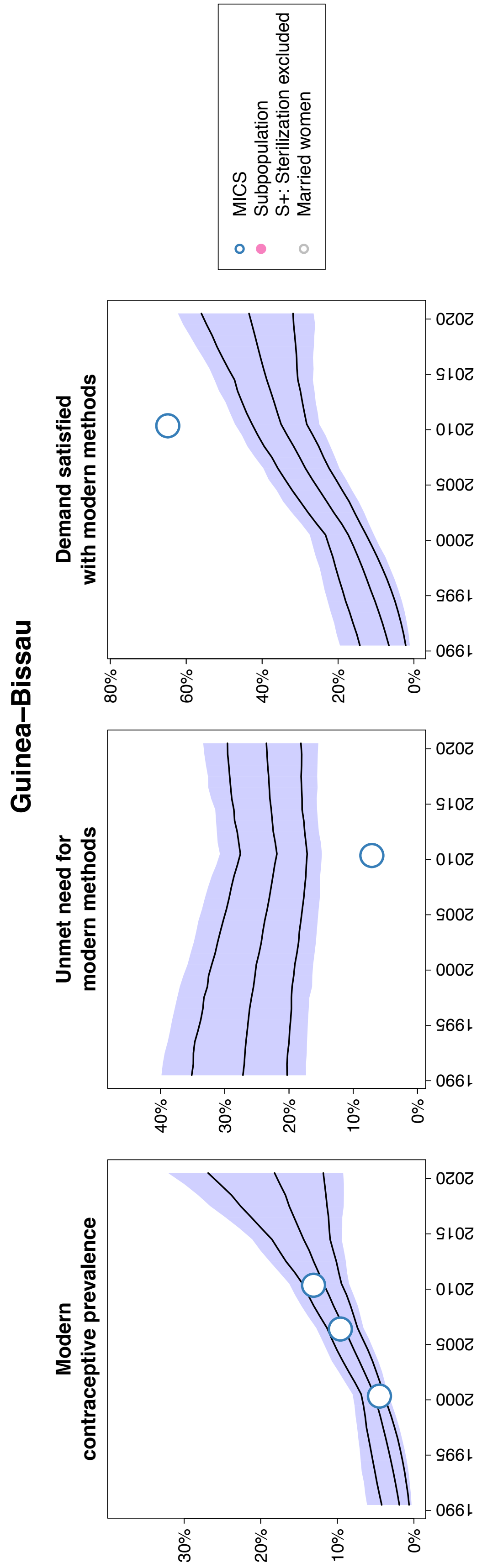

Guinea–Bissau
Modern
contraceptive prevalence
Unmet need for
modern methods
Demand satisfied
with modern methods
0%
10%
20%
30%
40%
60%
80%
1990
1995
2000
2005
2010
2015
2020
MICS
Subpopulation
S+: Sterilization excluded
Married women

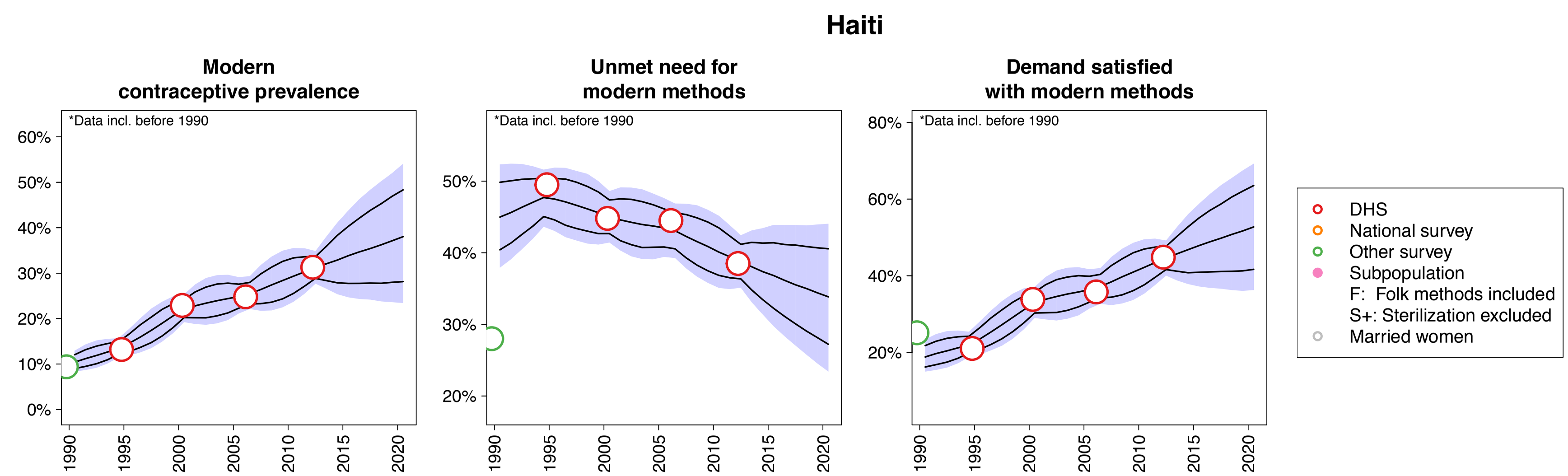
Haiti
Modern
contraceptive prevalence
*Data incl. before 1990
60%
50%
40%
30%
20%
10%
0%
1990
1995
2000
2005
2010
2015
2020
Unmet need for
modern methods
*Data incl. before 1990
50%
40%
30%
20%
Demand satisfied
with modern methods
*Data incl. before 1990
80%
60%
40%
20%
DHS
National survey
Other survey
Subpopulation
F: Folk methods included
S+: Sterilization excluded
Married women

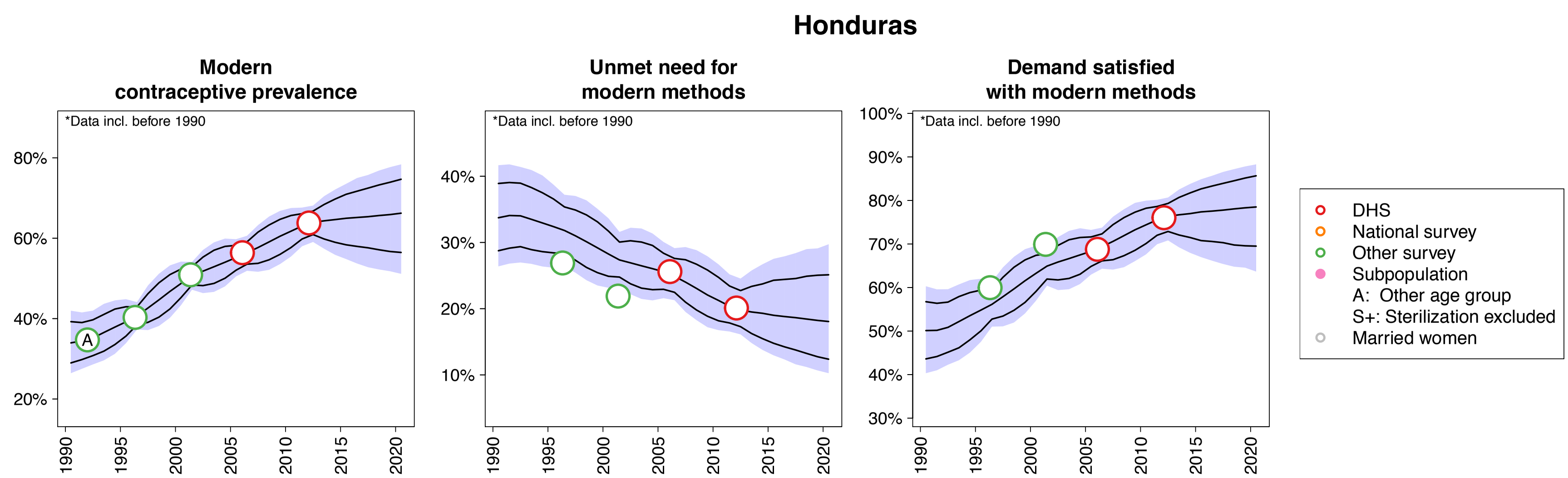
Honduras
Modern
contraceptive prevalence
*Data incl. before 1990
80%
60%
40%
20%
A
Unmet need for
modern methods
40%
30%
20%
10%
Demand satisfied
with modern methods
100%
90%
80%
70%
60%
50%
40%
30%
1990
1995
2000
2005
2010
2015
2020
DHS
National survey
Other survey
Subpopulation
A: Other age group
S+: Sterilization excluded
Married women

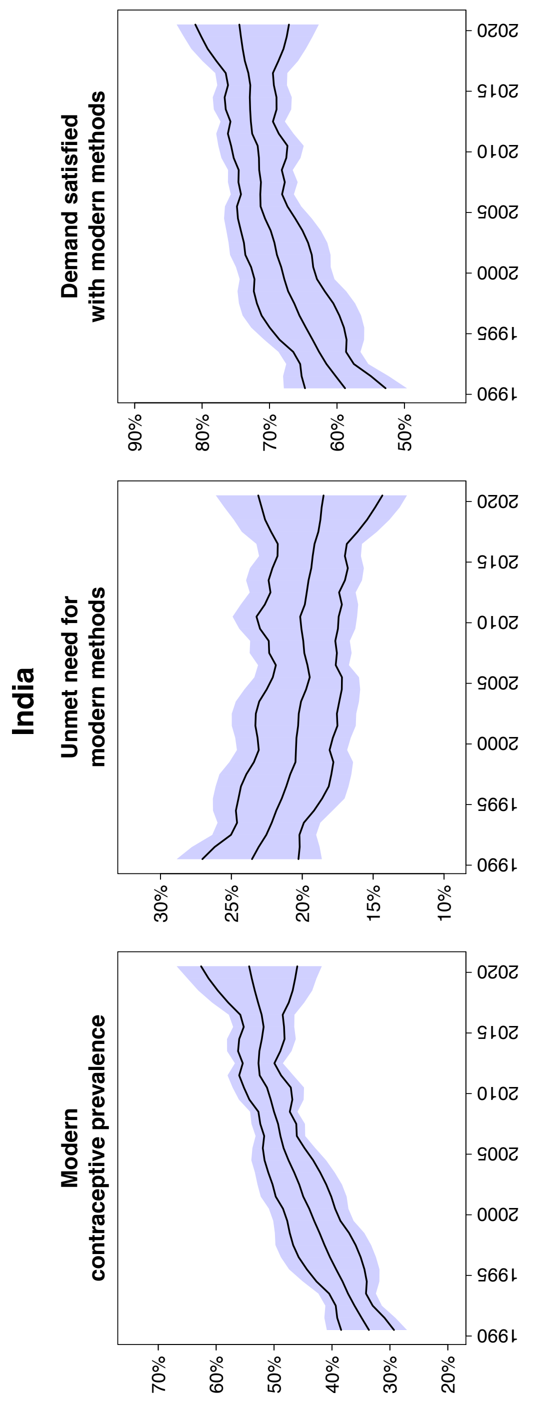
India
Modern
contraceptive prevalence
Unmet need for
modern methods
Demand satisfied
with modern methods
70%
60%
50%
40%
30%
20%
30%
25%
20%
15%
10%
90%
80%
70%
60%
50%
1990
1995
2000
2005
2010
2015
2020

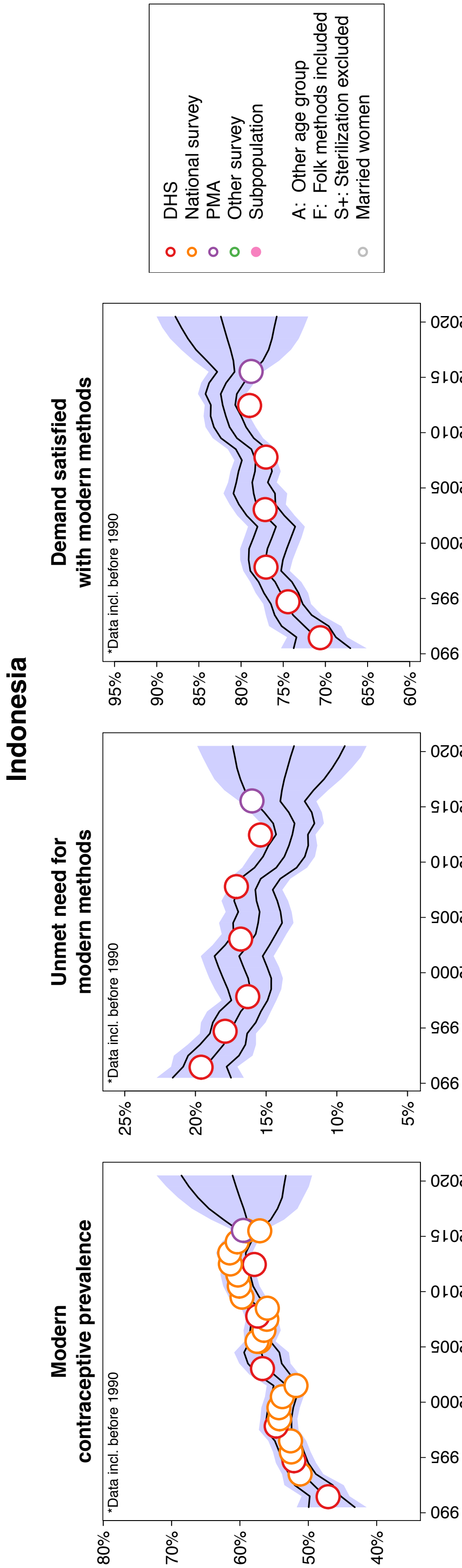

Indonesia
Modern
contraceptive prevalence
*Data incl. before 1990
80%
70%
60%
50%
40%
1990
1995
2000
2005
2010
2015
2020
Unmet need for
modern methods
*Data incl. before 1990
25%
20%
15%
10%
5%
Demand satisfied
with modern methods
*Data incl. before 1990
95%
90%
85%
80%
75%
70%
65%
60%
DHS
National survey
PMA
Other survey
Subpopulation
A: Other age group
F: Folk methods included
S+: Sterilization excluded
Married women

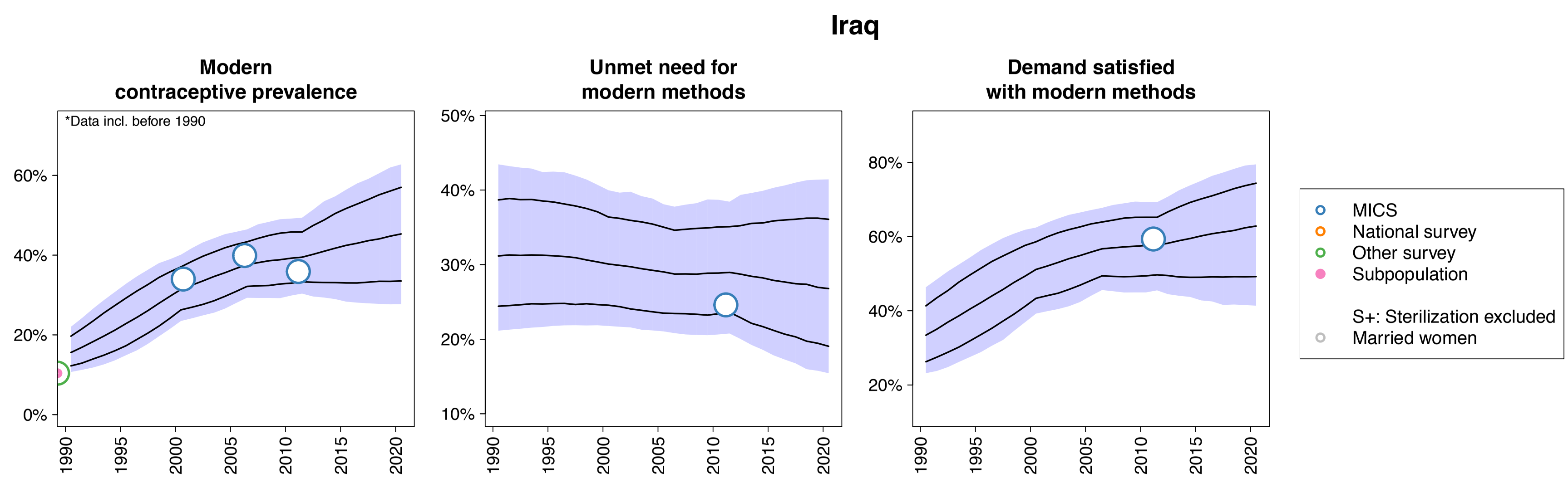
Iraq
Modern
contraceptive prevalence
*Data incl. before 1990
0%
20%
40%
60%
1990
1995
2000
2005
2010
2015
2020
Unmet need for
modern methods
10%
20%
30%
40%
50%
1990
1995
2000
2005
2010
2015
2020
Demand satisfied
with modern methods
20%
40%
60%
80%
1990
1995
2000
2005
2010
2015
2020
MICS
National survey
Other survey
Subpopulation
S+: Sterilization excluded
Married women

Kenya

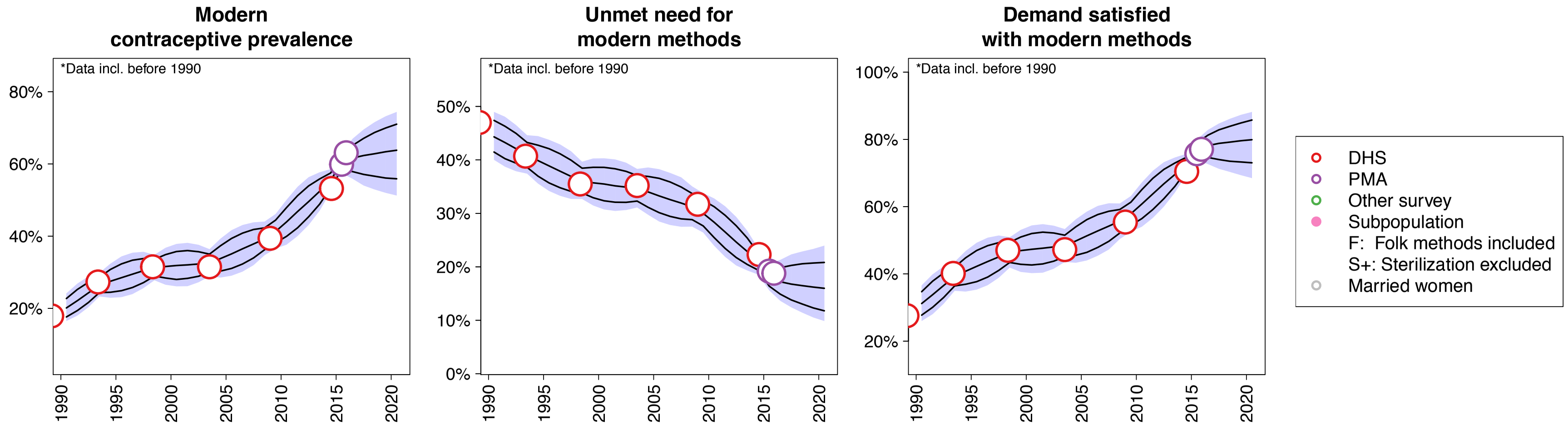
Modern
contraceptive prevalence
*Data incl. before 1990
80%
60%
40%
20%
1990
1995
2000
2005
2010
2015
2020
Unmet need for
modern methods
*Data incl. before 1990
50%
40%
30%
20%
10%
0%
1990
1995
2000
2005
2010
2015
2020
Demand satisfied
with modern methods
*Data incl. before 1990
100%
80%
60%
40%
20%
1990
1995
2000
2005
2010
2015
2020
DHS
PMA
Other survey
Subpopulation
F: Folk methods included
S+: Sterilization excluded
Married women

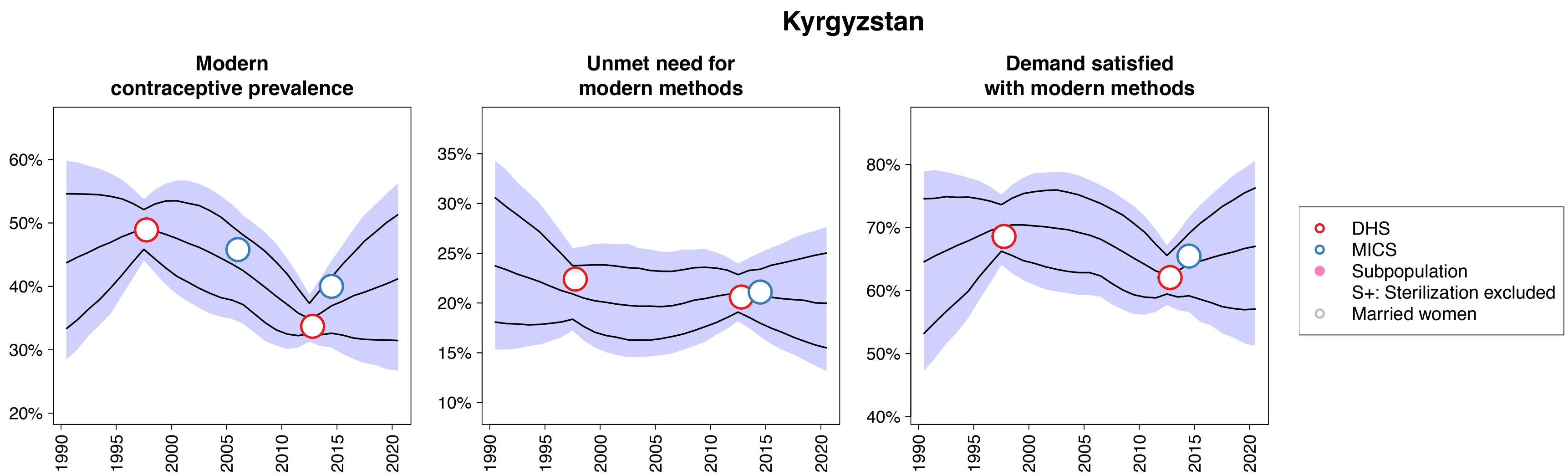
Kyrgyzstan
Modern
contraceptive prevalence
Unmet need for
modern methods
Demand satisfied
with modern methods
60%
50%
40%
30%
20%
35%
30%
25%
20%
15%
10%
80%
70%
60%
50%
40%
1990
1995
2000
2005
2010
2015
2020
DHS
MICS
Subpopulation
S+: Sterilization excluded
Married women

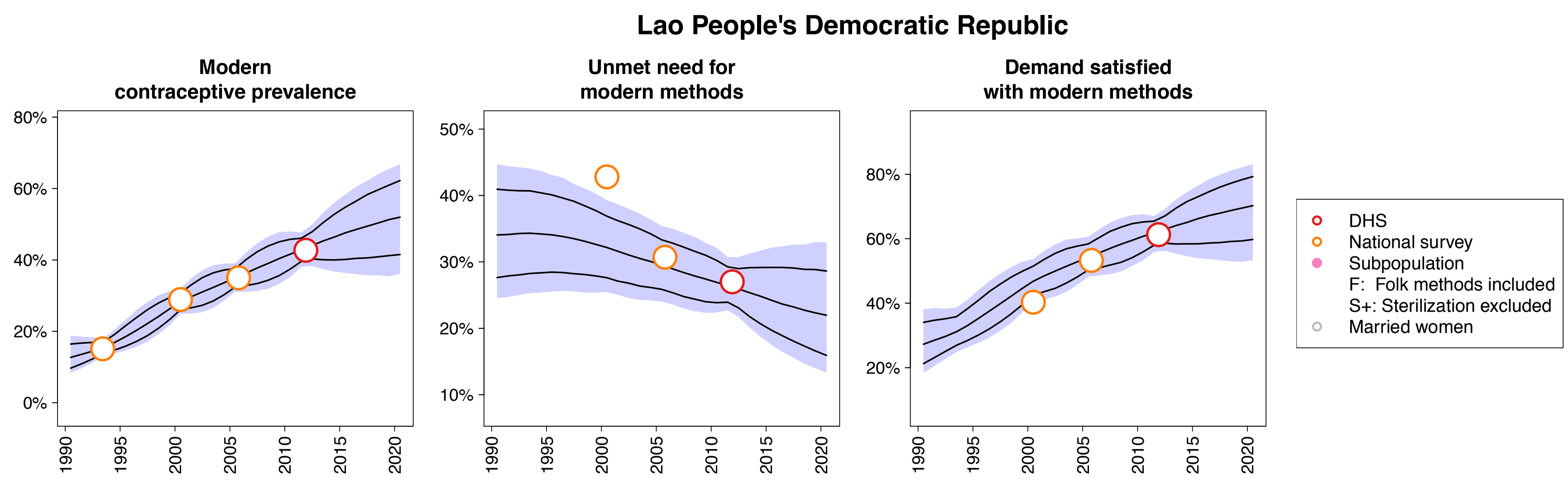
Lao People's Democratic Republic
Modern contraceptive prevalence
Unmet need for modern methods
Demand satisfied with modern methods
80%
60%
40%
20%
0%
50%
30%
10%
1990
1995
2000
2005
2010
2015
2020
DHS
National survey
Subpopulation
F: Folk methods included
S+: Sterilization excluded
Married women

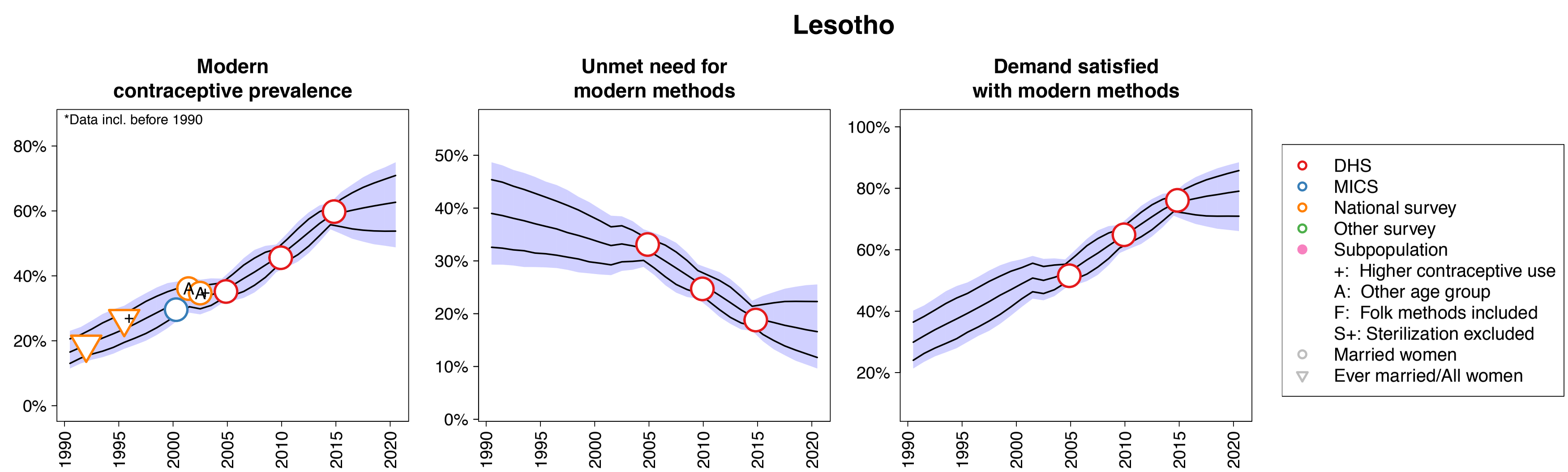
Lesotho
Modern
contraceptive prevalence
*Data incl. before 1990
Unmet need for
modern methods
Demand satisfied
with modern methods
DHS
MICS
National survey
Other survey
Subpopulation
+: Higher contraceptive use
A: Other age group
F: Folk methods included
S+: Sterilization excluded
Married women
Ever married/All women

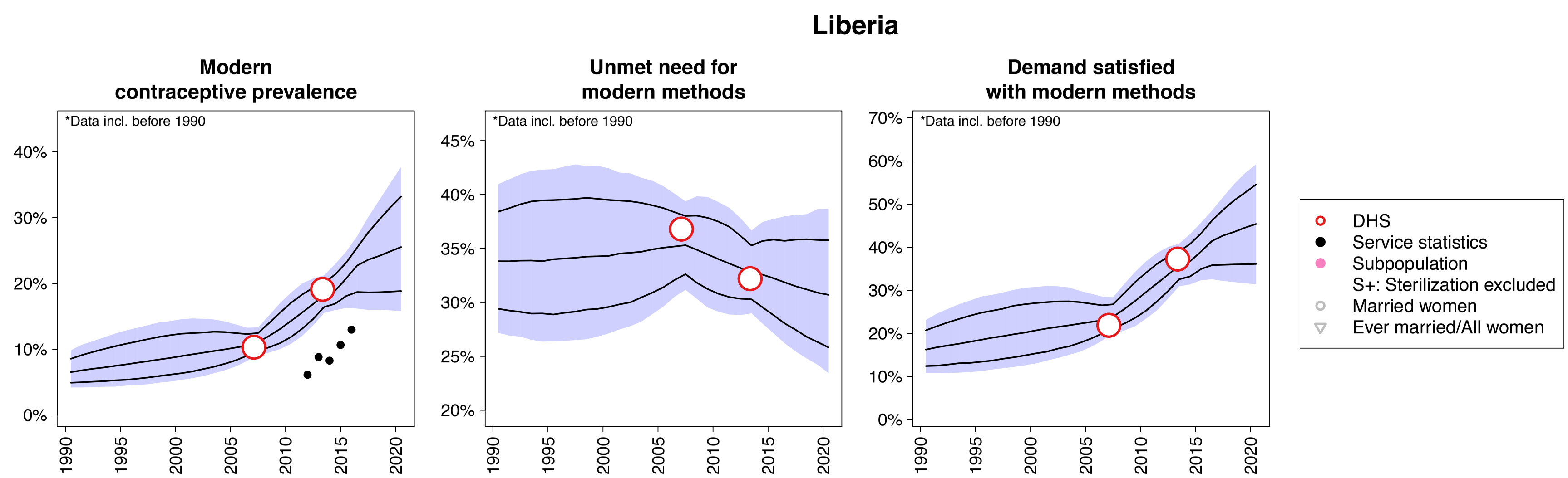
Liberia
Modern contraceptive prevalence
Unmet need for modern methods
Demand satisfied with modern methods
*Data incl. before 1990
0%
10%
20%
30%
40%
45%
35%
25%
50%
60%
70%
1990
1995
2000
2005
2010
2015
2020
DHS
Service statistics
Subpopulation
S+: Sterilization excluded
Married women
Ever married/All women

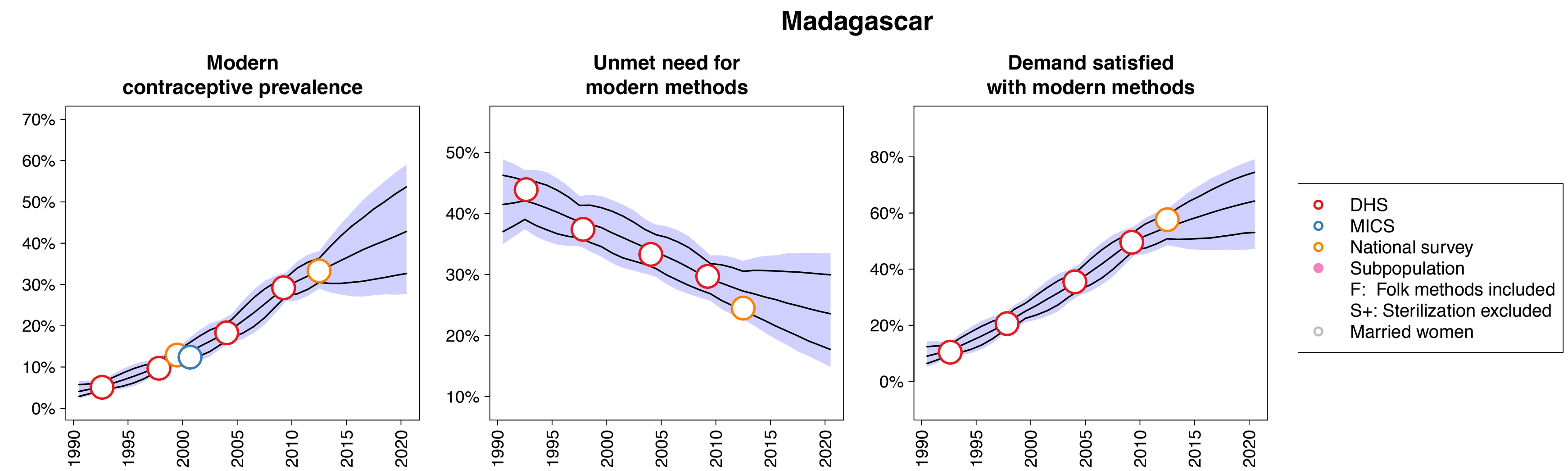
Madagascar
Modern contraceptive prevalence
Unmet need for modern methods
Demand satisfied with modern methods
0%
10%
20%
30%
40%
50%
60%
70%
80%
1990
1995
2000
2005
2010
2015
2020
DHS
MICS
National survey
Subpopulation
F: Folk methods included
S+: Sterilization excluded
Married women

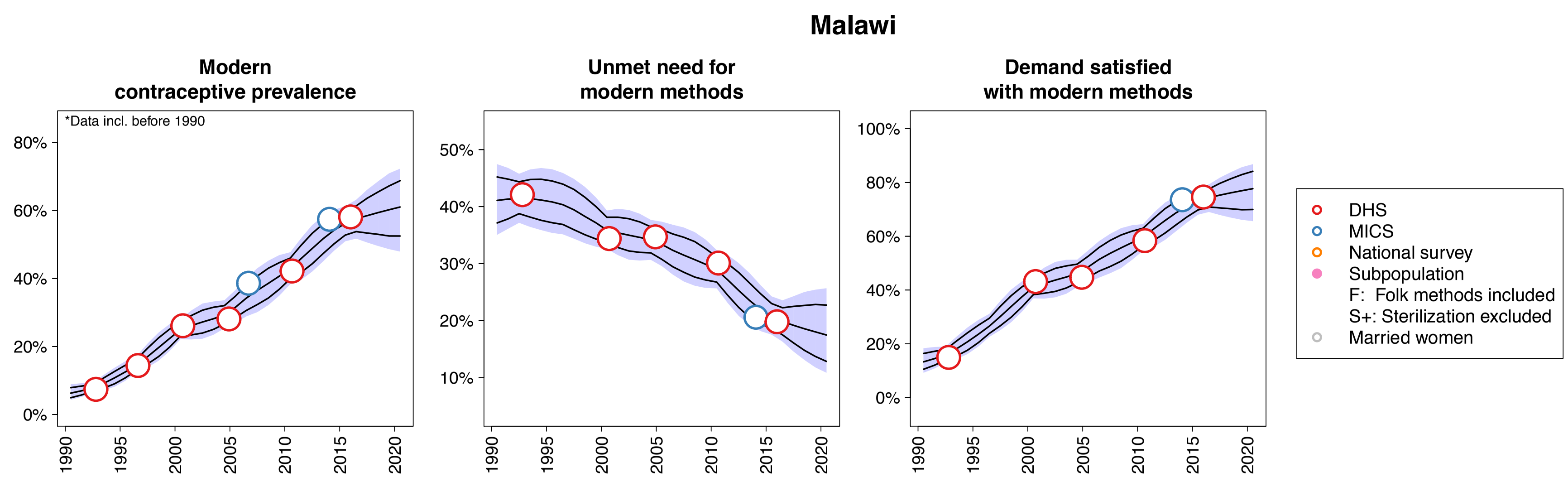
Malawi
Modern contraceptive prevalence
*Data incl. before 1990
Unmet need for modern methods
Demand satisfied with modern methods
DHS
MICS
National survey
Subpopulation
F: Folk methods included
S+: Sterilization excluded
Married women

Mali

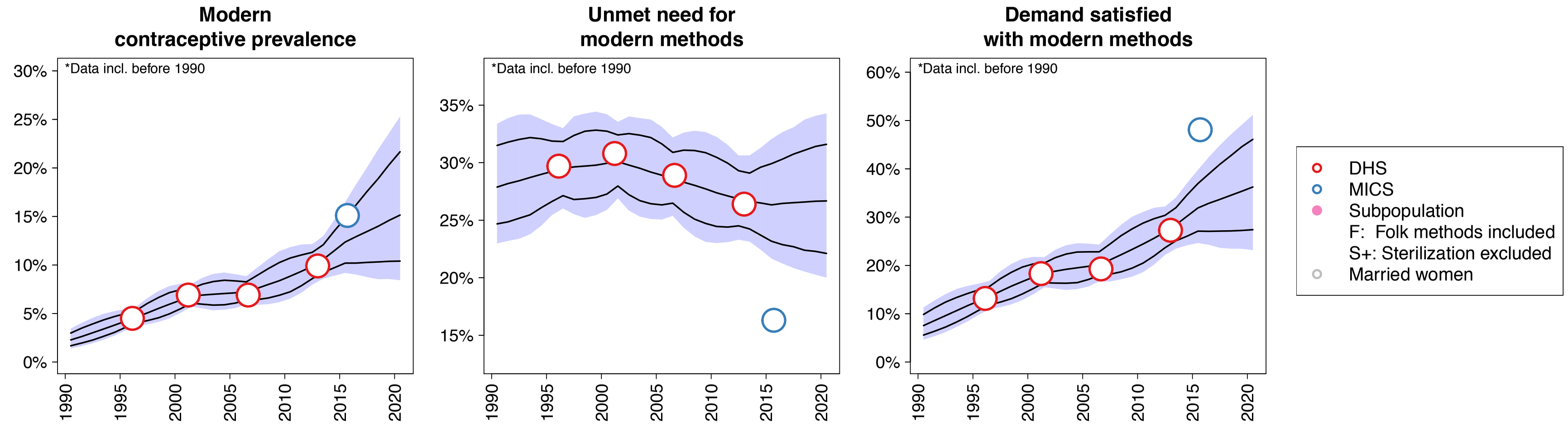
Modern
contraceptive prevalence
*Data incl. before 1990
Unmet need for
modern methods
*Data incl. before 1990
Demand satisfied
with modern methods
*Data incl. before 1990
DHS
MICS
Subpopulation
F: Folk methods included
S+: Sterilization excluded
Married women

Mauritania

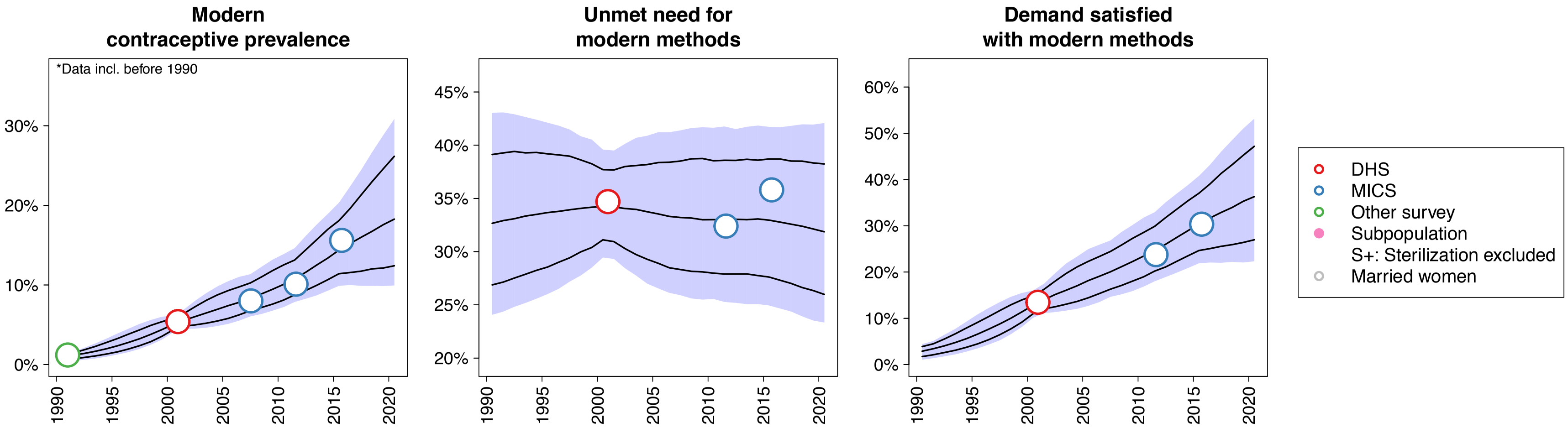
Modern
contraceptive prevalence
*Data incl. before 1990
Unmet need for
modern methods
Demand satisfied
with modern methods
DHS
MICS
Other survey
Subpopulation
S+: Sterilization excluded
Married women

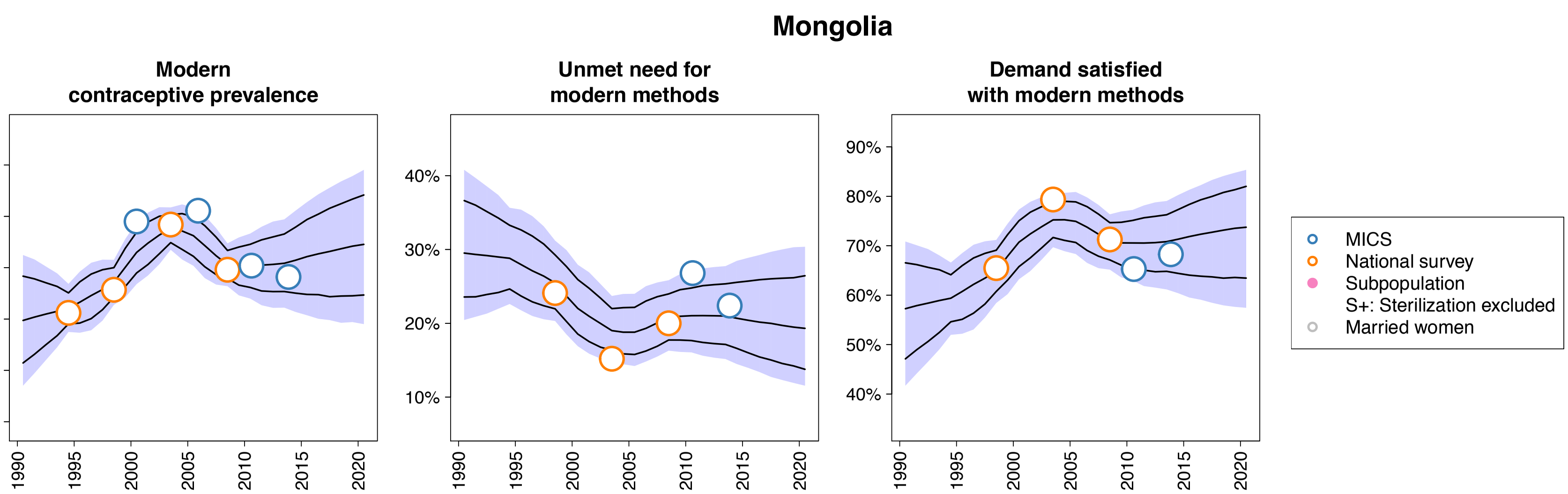

Mongolia
Modern
contraceptive prevalence
Unmet need for
modern methods
Demand satisfied
with modern methods
70%
60%
50%
40%
30%
20%
10%
90%
80%
1990
1995
2000
2005
2010
2015
2020
MICS
National survey
Subpopulation
S+: Sterilization excluded
Married women

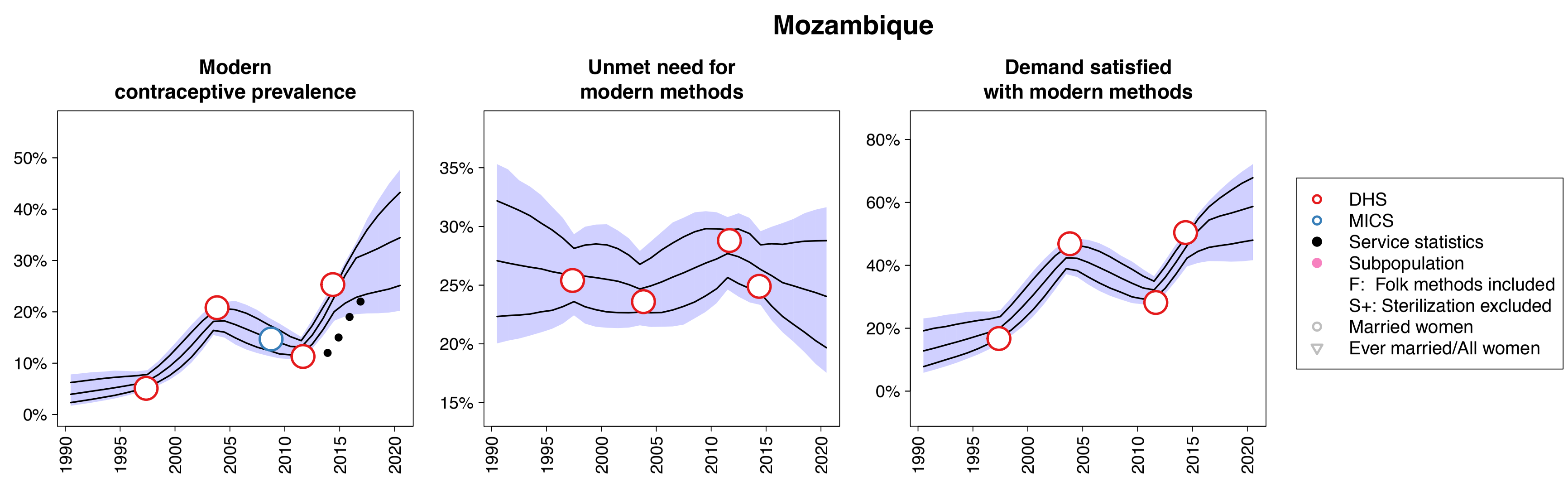
Mozambique
Modern contraceptive prevalence
Unmet need for modern methods
Demand satisfied with modern methods
DHS
MICS
Service statistics
Subpopulation
F: Folk methods included
S+: Sterilization excluded
Married women
Ever married/All women

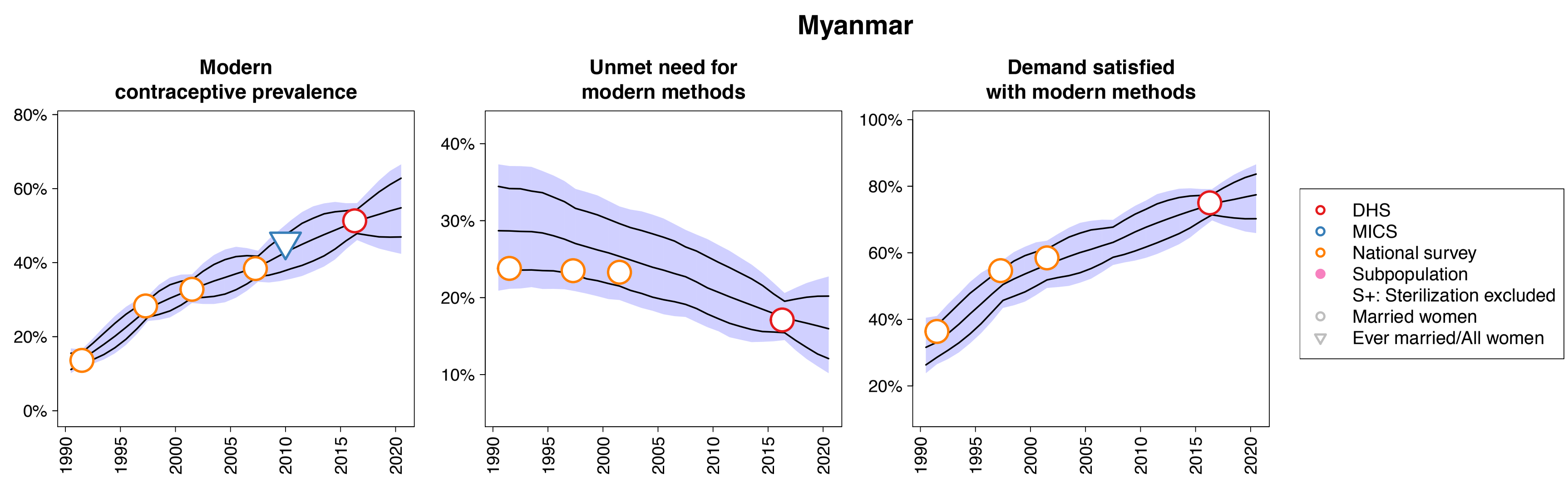
Myanmar
Modern contraceptive prevalence
Unmet need for modern methods
Demand satisfied with modern methods
0%
20%
40%
60%
80%
10%
30%
100%
1990
1995
2000
2005
2010
2015
2020
DHS
MICS
National survey
Subpopulation
S+: Sterilization excluded
Married women
Ever married/All women

Nepal

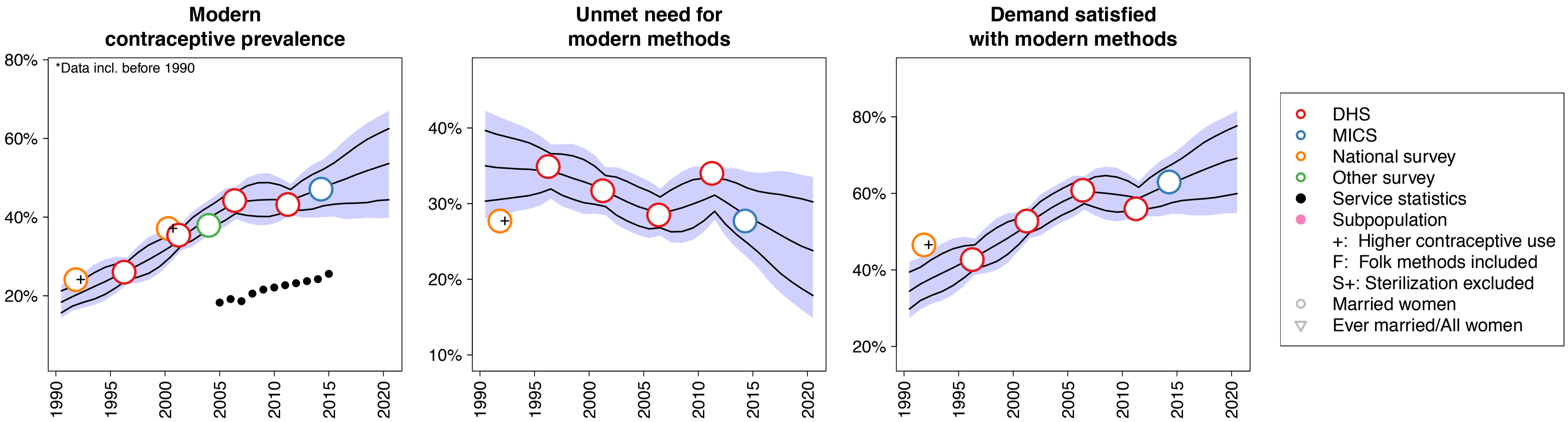
Modern contraceptive prevalence
*Data incl. before 1990
Unmet need for modern methods
Demand satisfied with modern methods
DHS
MICS
National survey
Other survey
Service statistics
Subpopulation
+: Higher contraceptive use
F: Folk methods included
S+: Sterilization excluded
Married women
Ever married/All women

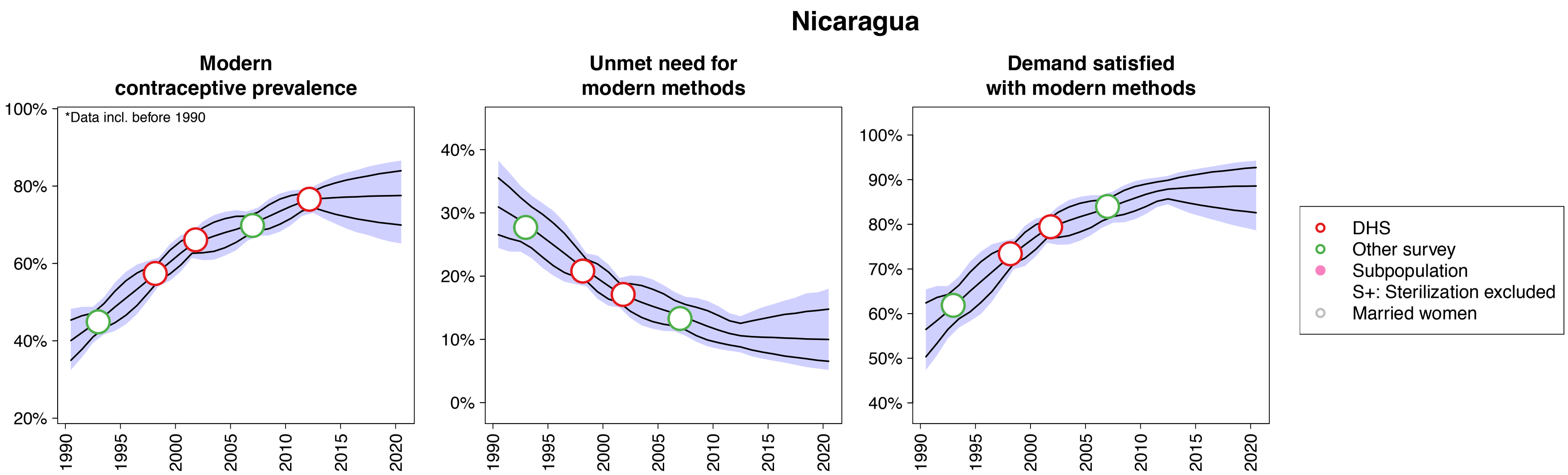
Nicaragua
Modern
contraceptive prevalence
*Data incl. before 1990
100%
80%
60%
40%
20%
1990
1995
2000
2005
2010
2015
2020
Unmet need for
modern methods
40%
30%
20%
10%
0%
Demand satisfied
with modern methods
100%
90%
80%
70%
60%
50%
40%
DHS
Other survey
Subpopulation
S+: Sterilization excluded
Married women

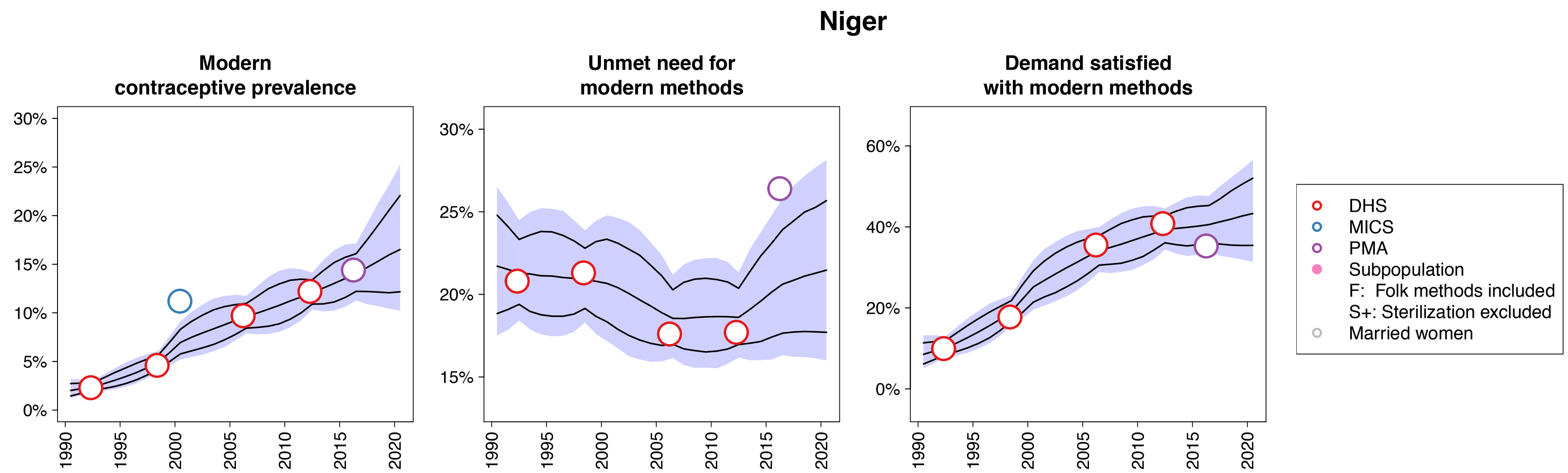
Niger
Modern
contraceptive prevalence
Unmet need for
modern methods
Demand satisfied
with modern methods
0%
5%
10%
15%
20%
25%
30%
40%
60%
1990
1995
2000
2005
2010
2015
2020
DHS
MICS
PMA
Subpopulation
F: Folk methods included
S+: Sterilization excluded
Married women

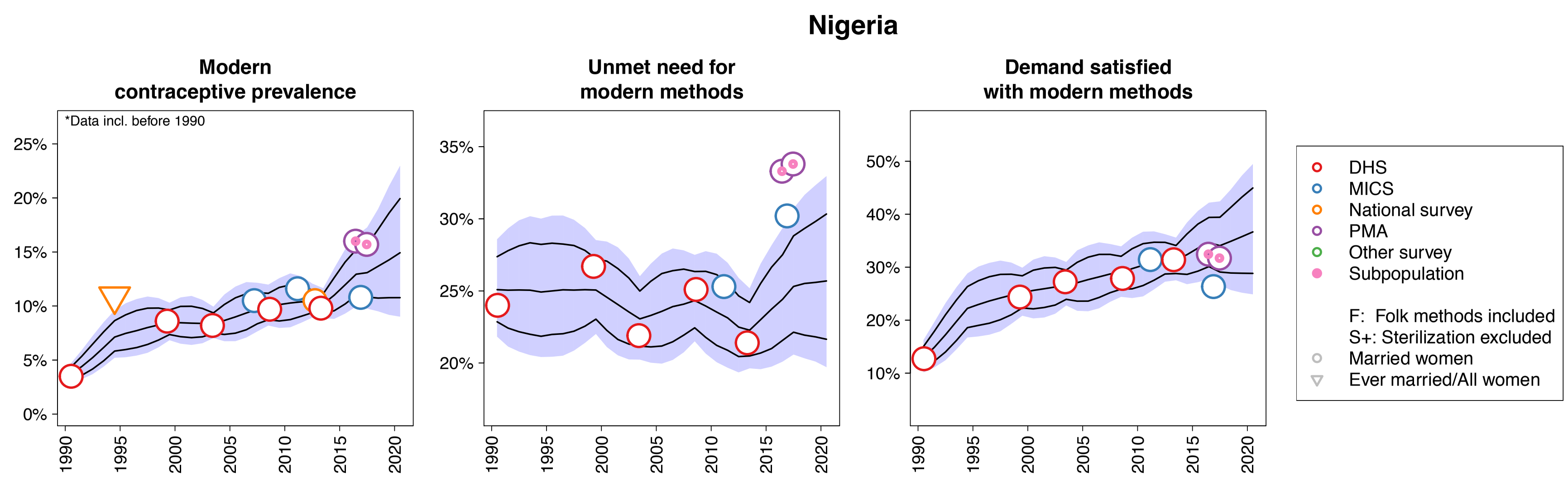
Nigeria
Modern contraceptive prevalence
*Data incl. before 1990
0%
5%
10%
15%
20%
25%
Unmet need for modern methods
20%
25%
30%
35%
Demand satisfied with modern methods
10%
20%
30%
40%
50%
1990
1995
2000
2005
2010
2015
2020
DHS
MICS
National survey
PMA
Other survey
Subpopulation
F: Folk methods included
S+: Sterilization excluded
Married women
Ever married/All women

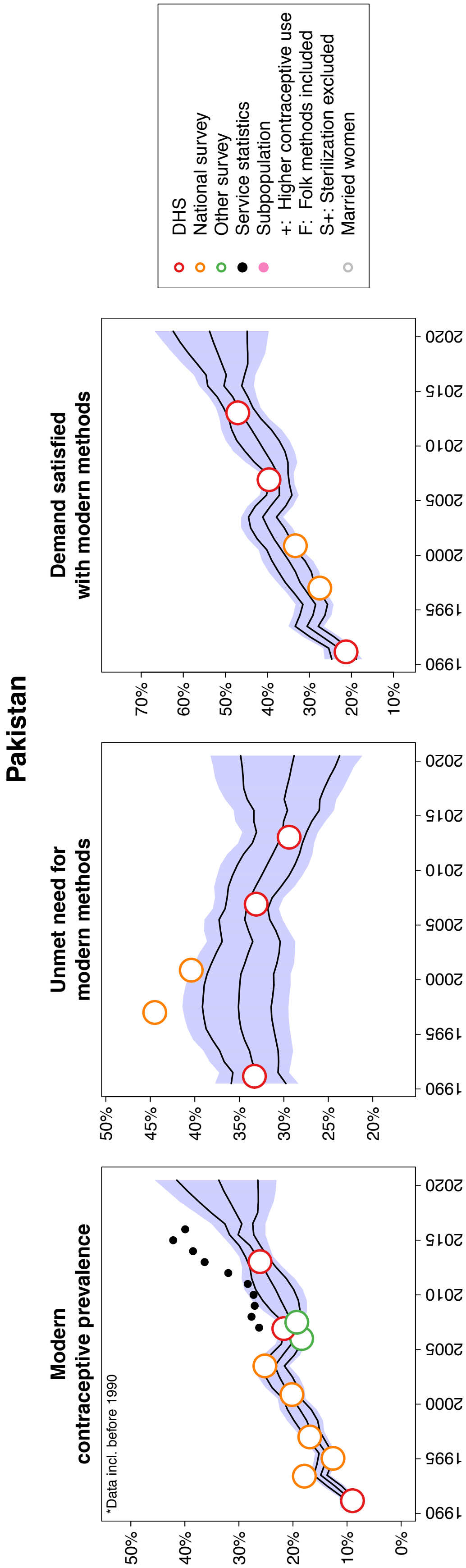

Pakistan
Modern contraceptive prevalence
*Data incl. before 1990
Unmet need for modern methods
Demand satisfied with modern methods
DHS
National survey
Other survey
Service statistics
Subpopulation
+: Higher contraceptive use
F: Folk methods included
S+: Sterilization excluded
Married women

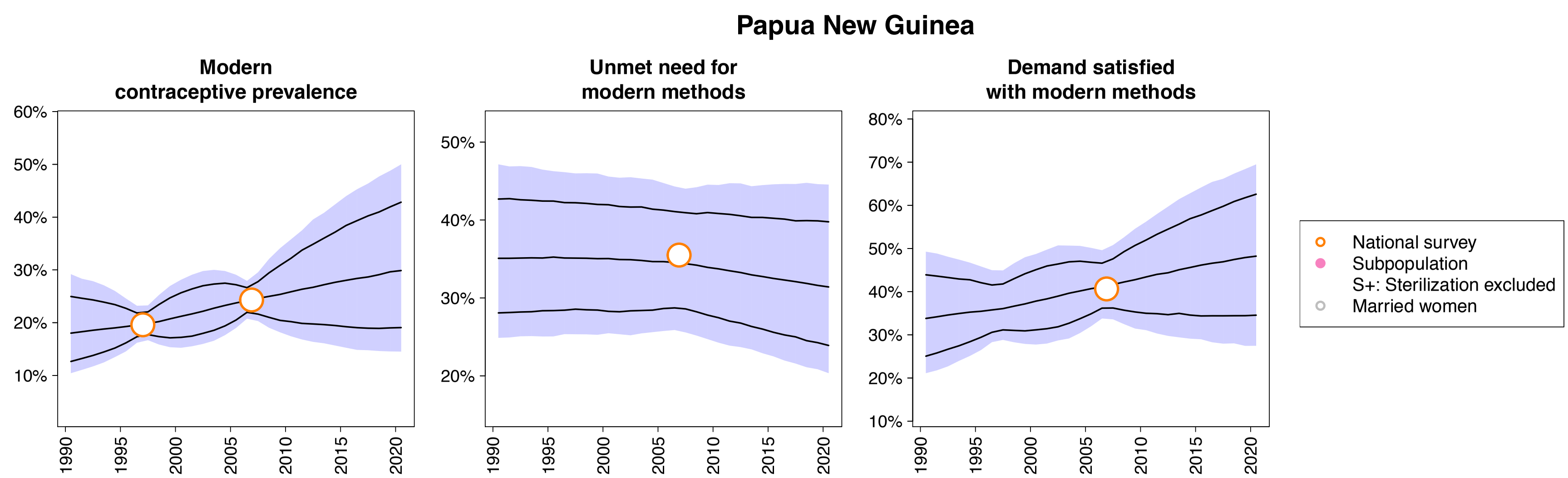
Papua New Guinea
Modern
contraceptive prevalence
60%
50%
40%
30%
20%
10%
1990
1995
2000
2005
2010
2015
2020
Unmet need for
modern methods
50%
40%
30%
20%
Demand satisfied
with modern methods
80%
70%
60%
50%
40%
30%
20%
10%
National survey
Subpopulation
S+: Sterilization excluded
Married women

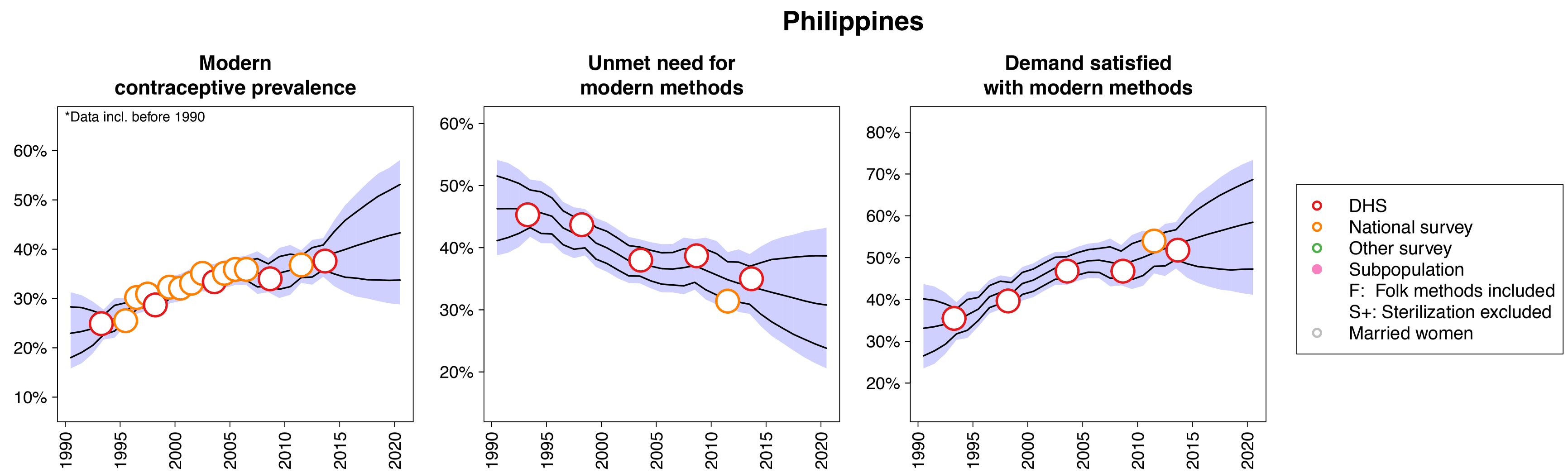
Philippines
Modern
contraceptive prevalence
*Data incl. before 1990
60%
50%
40%
30%
20%
10%
1990
1995
2000
2005
2010
2015
2020
Unmet need for
modern methods
60%
50%
40%
30%
20%
1990
1995
2000
2005
2010
2015
2020
Demand satisfied
with modern methods
80%
70%
60%
50%
40%
30%
20%
1990
1995
2000
2005
2010
2015
2020
DHS
National survey
Other survey
Subpopulation
F: Folk methods included
S+: Sterilization excluded
Married women

Rwanda

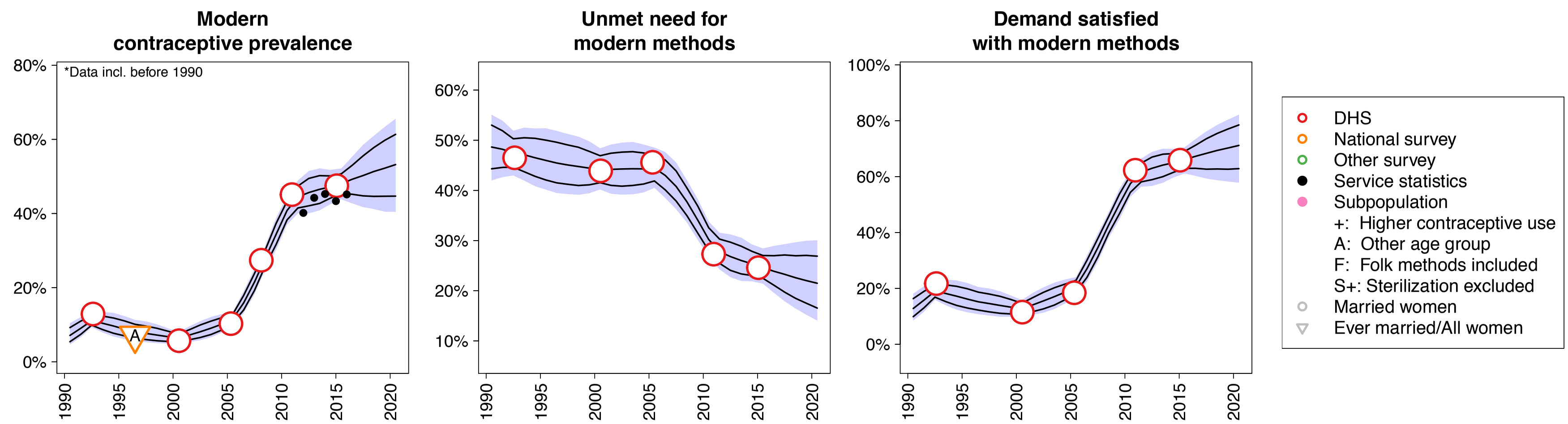

Modern contraceptive prevalence
*Data incl. before 1990
80%
60%
40%
20%
0%
A
1990
1995
2000
2005
2010
2015
2020
Unmet need for modern methods
60%
50%
40%
30%
20%
10%
1990
1995
2000
2005
2010
2015
2020
Demand satisfied with modern methods
100%
80%
60%
40%
20%
0%
1990
1995
2000
2005
2010
2015
2020
DHS
National survey
Other survey
Service statistics
Subpopulation
+: Higher contraceptive use
A: Other age group
F: Folk methods included
S+: Sterilization excluded
Married women
Ever married/All women

Sao Tome and Principe

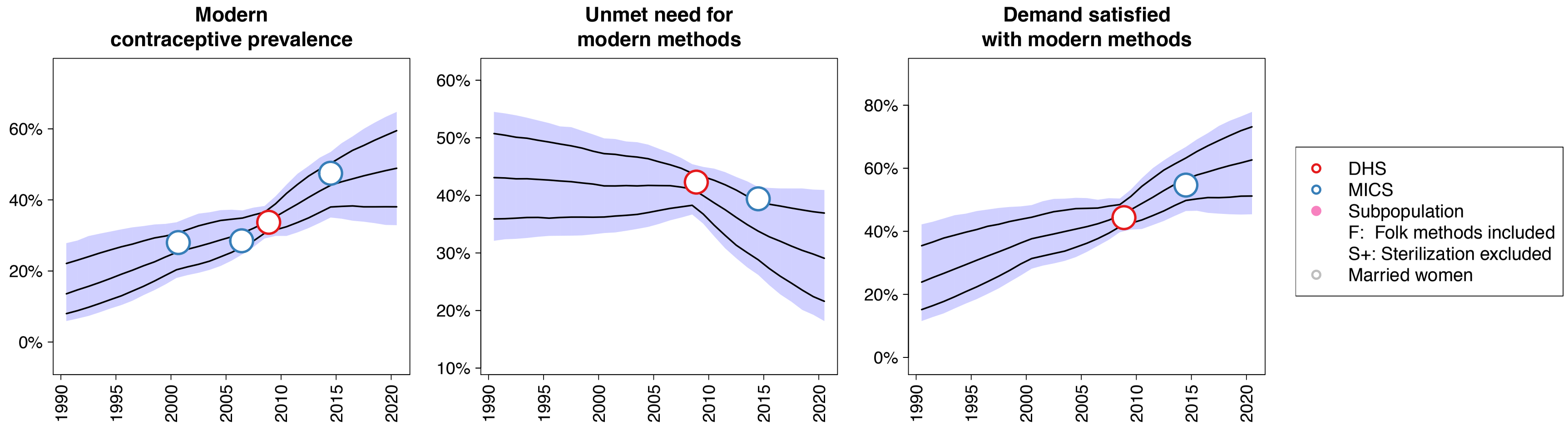

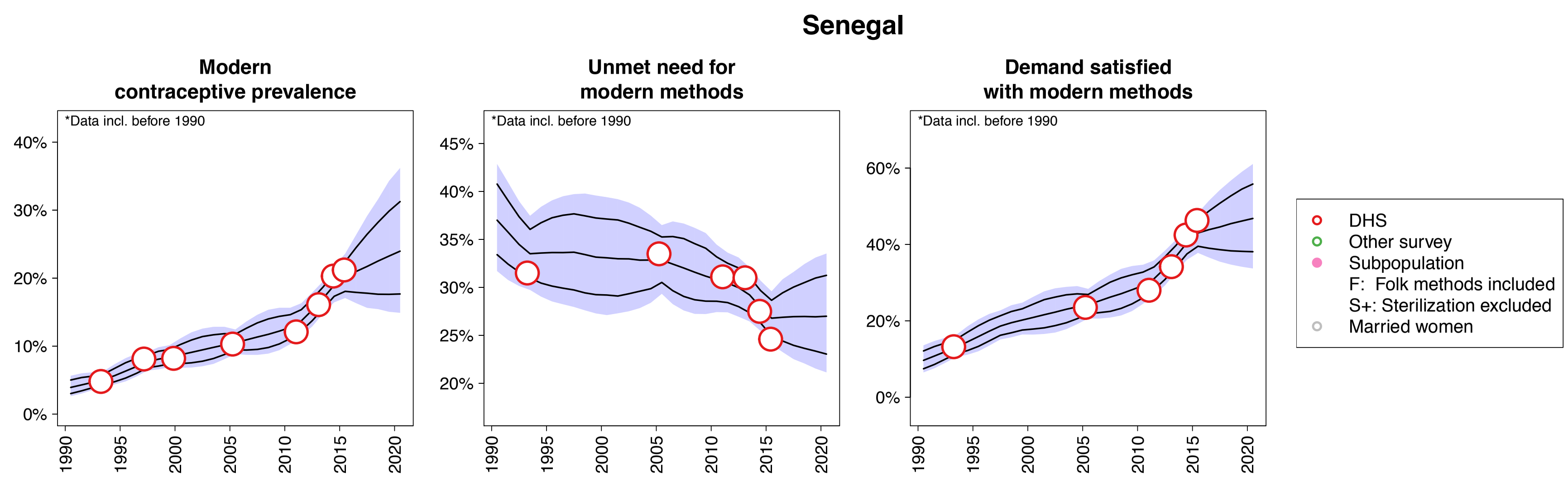
Senegal
Modern contraceptive prevalence
*Data incl. before 1990
0%
10%
20%
30%
40%
1990
1995
2000
2005
2010
2015
2020
Unmet need for modern methods
*Data incl. before 1990
20%
25%
30%
35%
40%
45%
1990
1995
2000
2005
2010
2015
2020
Demand satisfied with modern methods
*Data incl. before 1990
0%
20%
40%
60%
1990
1995
2000
2005
2010
2015
2020
DHS
Other survey
Subpopulation
F: Folk methods included
S+: Sterilization excluded
Married women

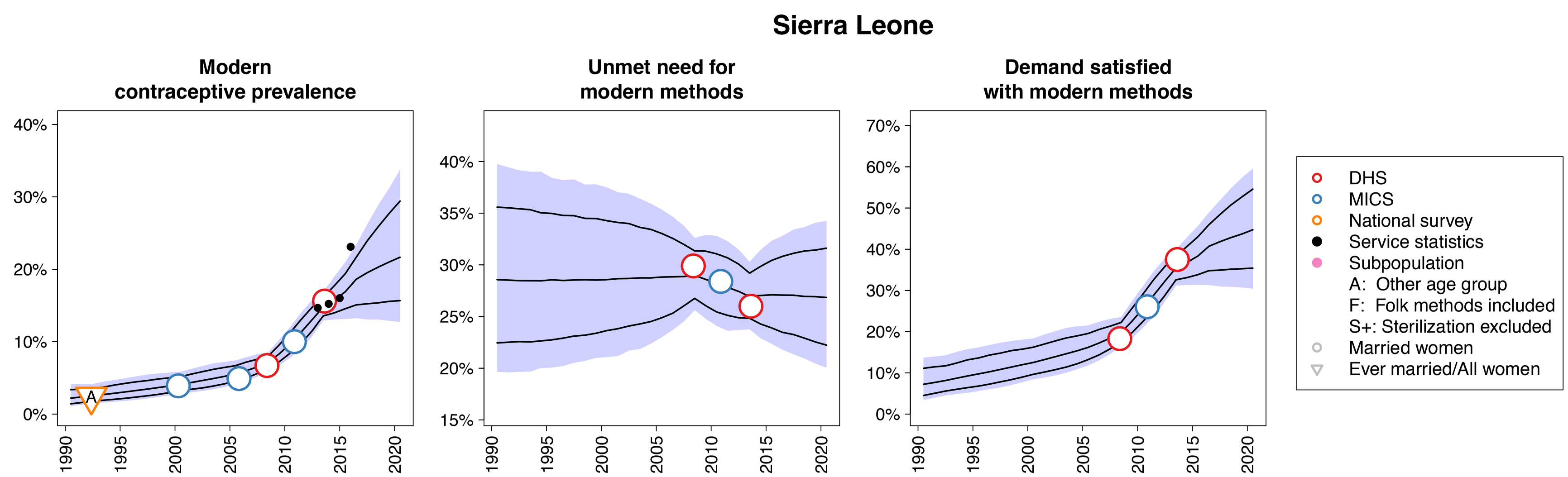
Sierra Leone
Modern contraceptive prevalence
Unmet need for modern methods
Demand satisfied with modern methods
0%
10%
20%
30%
40%
15%
25%
35%
50%
60%
70%
1990
1995
2000
2005
2010
2015
2020
A
DHS
MICS
National survey
Service statistics
Subpopulation
A: Other age group
F: Folk methods included
S+: Sterilization excluded
Married women
Ever married/All women

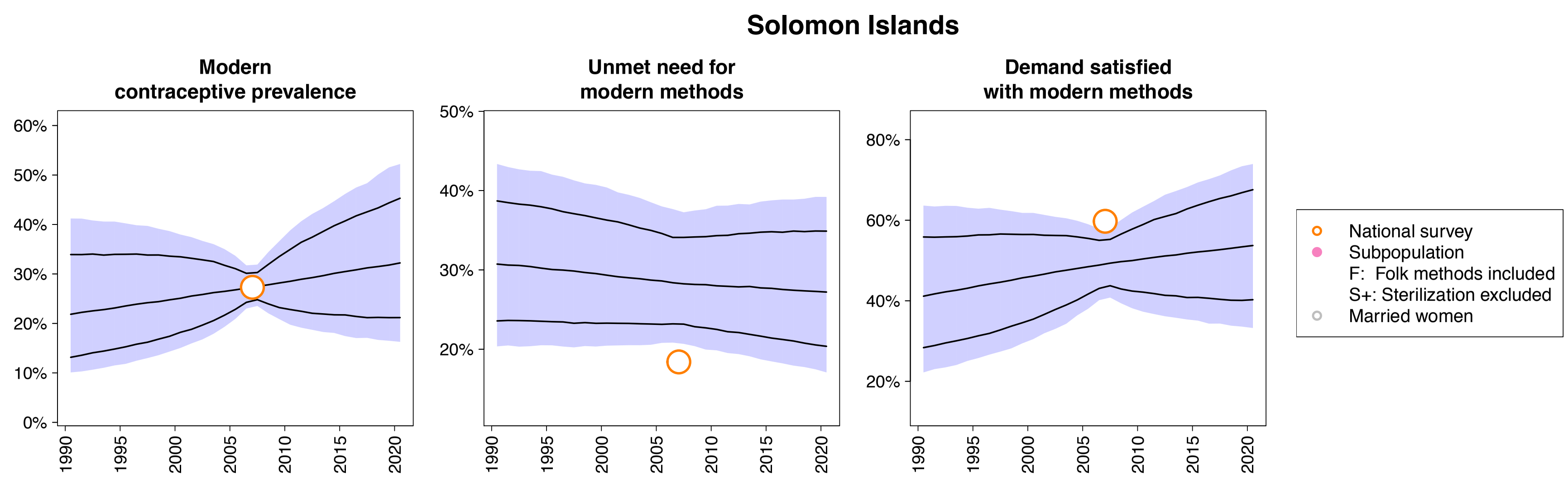

Solomon Islands
Modern
contraceptive prevalence
Unmet need for
modern methods
Demand satisfied
with modern methods
60%
50%
40%
30%
20%
10%
0%
1990
1995
2000
2005
2010
2015
2020
80%
National survey
Subpopulation
F: Folk methods included
S+: Sterilization excluded
Married women

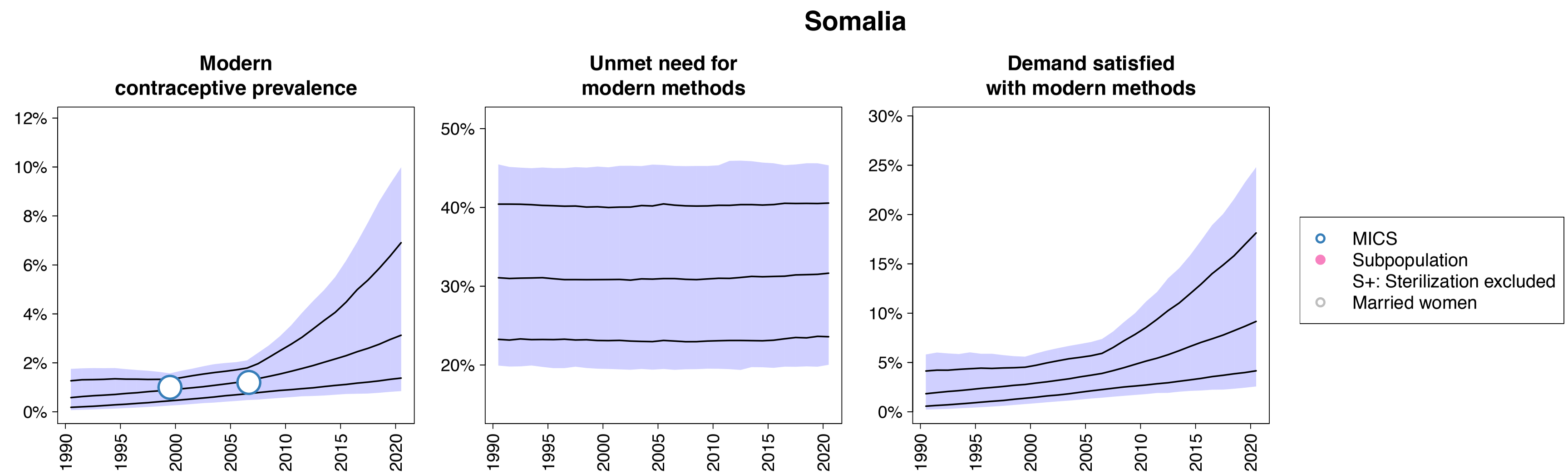
Somalia
Modern contraceptive prevalence
Unmet need for modern methods
Demand satisfied with modern methods
MICS
Subpopulation
S+: Sterilization excluded
Married women

South Sudan

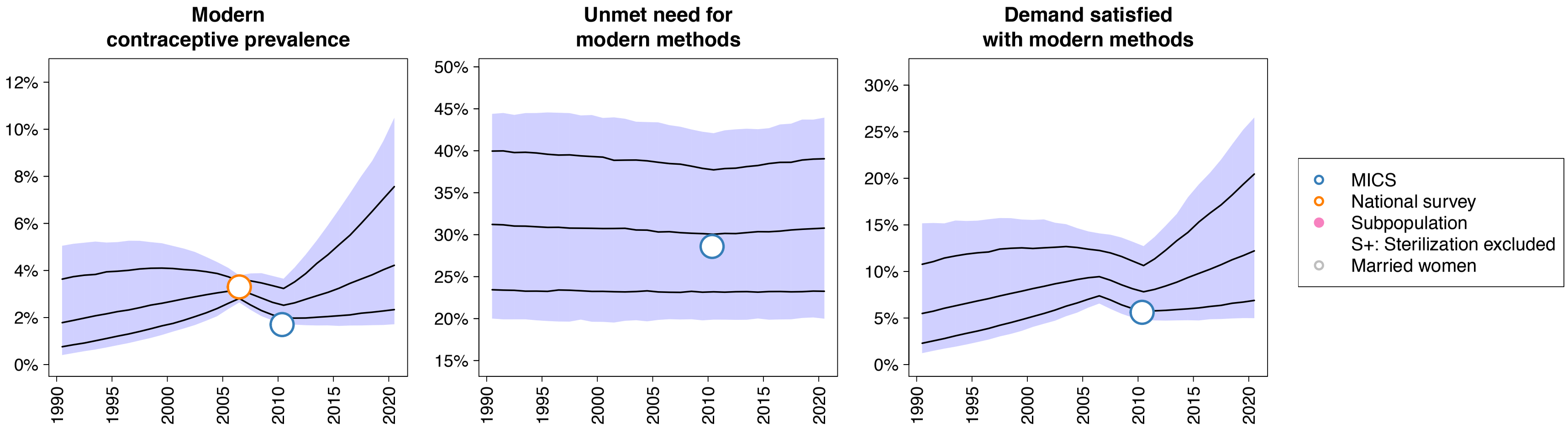
Modern
contraceptive prevalence
Unmet need for
modern methods
Demand satisfied
with modern methods
0%
2%
4%
6%
8%
10%
12%
15%
20%
25%
30%
35%
40%
45%
50%
5%
1990
1995
2000
2005
2010
2015
2020
MICS
National survey
Subpopulation
S+: Sterilization excluded
Married women

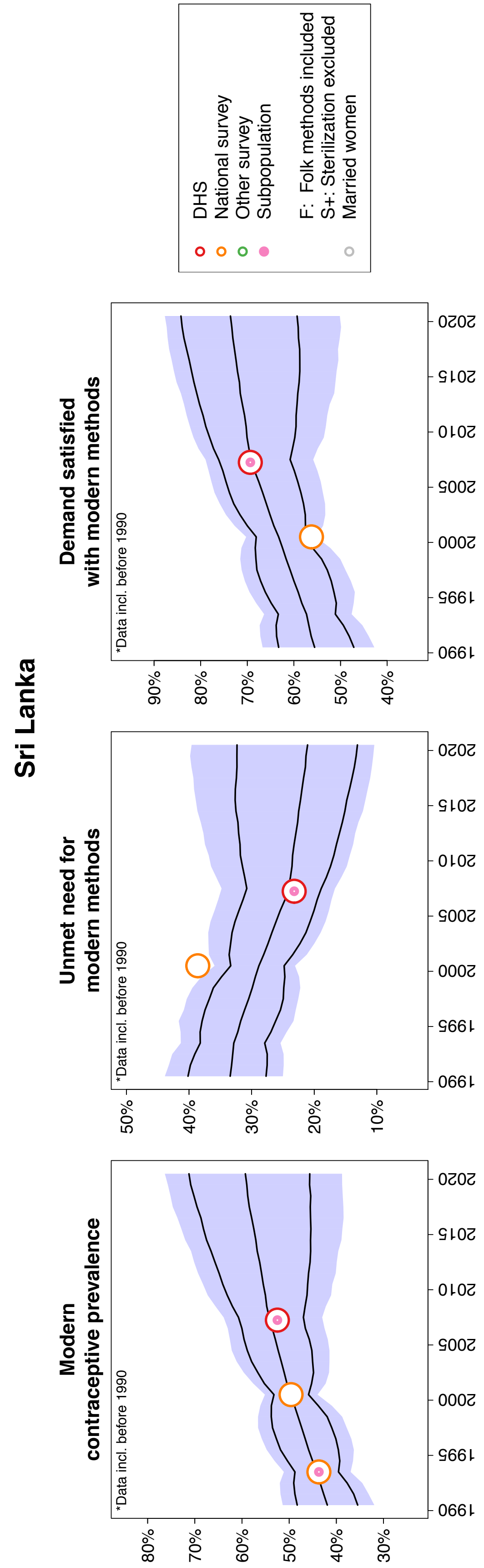
Sri Lanka
Modern contraceptive prevalence
Unmet need for modern methods
Demand satisfied with modern methods
*Data incl. before 1990
80%
70%
60%
50%
40%
30%
20%
10%
90%
1990
1995
2000
2005
2010
2015
2020
DHS
National survey
Other survey
Subpopulation
F: Folk methods included
S+: Sterilization excluded
Married women

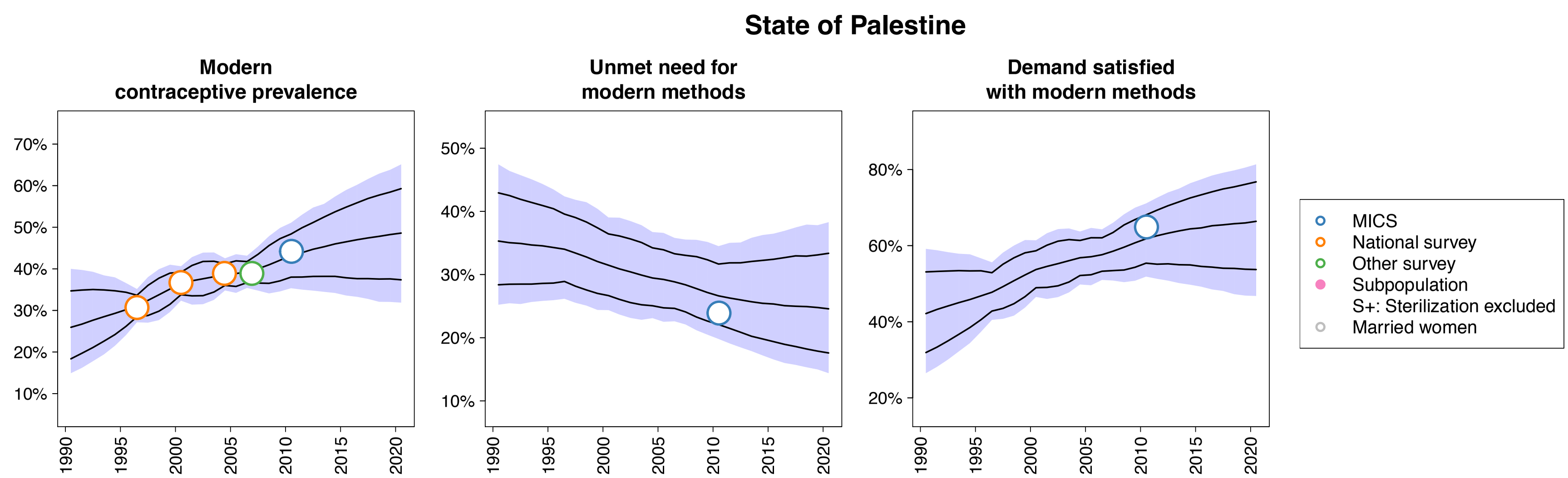
State of Palestine
Modern contraceptive prevalence
Unmet need for modern methods
Demand satisfied with modern methods
70%
60%
50%
40%
30%
20%
10%
80%
1990
1995
2000
2005
2010
2015
2020
MICS
National survey
Other survey
Subpopulation
S+: Sterilization excluded
Married women

Sudan

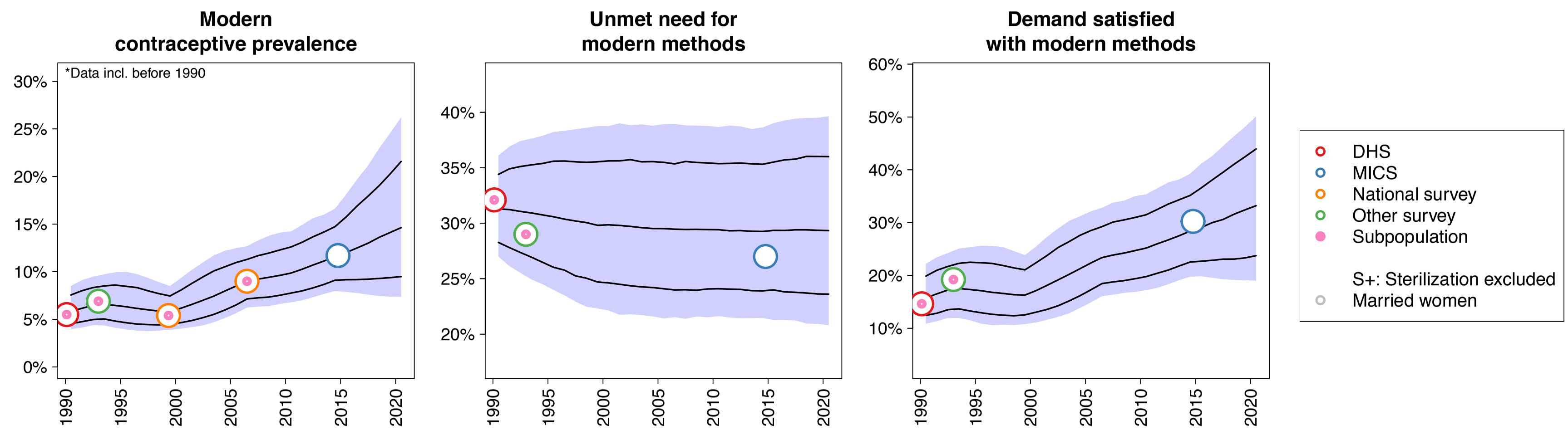
Modern
contraceptive prevalence
*Data incl. before 1990
30%
25%
20%
15%
10%
5%
0%
1990
1995
2000
2005
2010
2015
2020
Unmet need for
modern methods
40%
35%
30%
25%
20%
Demand satisfied
with modern methods
60%
50%
40%
30%
20%
10%
DHS
MICS
National survey
Other survey
Subpopulation
S+: Sterilization excluded
Married women

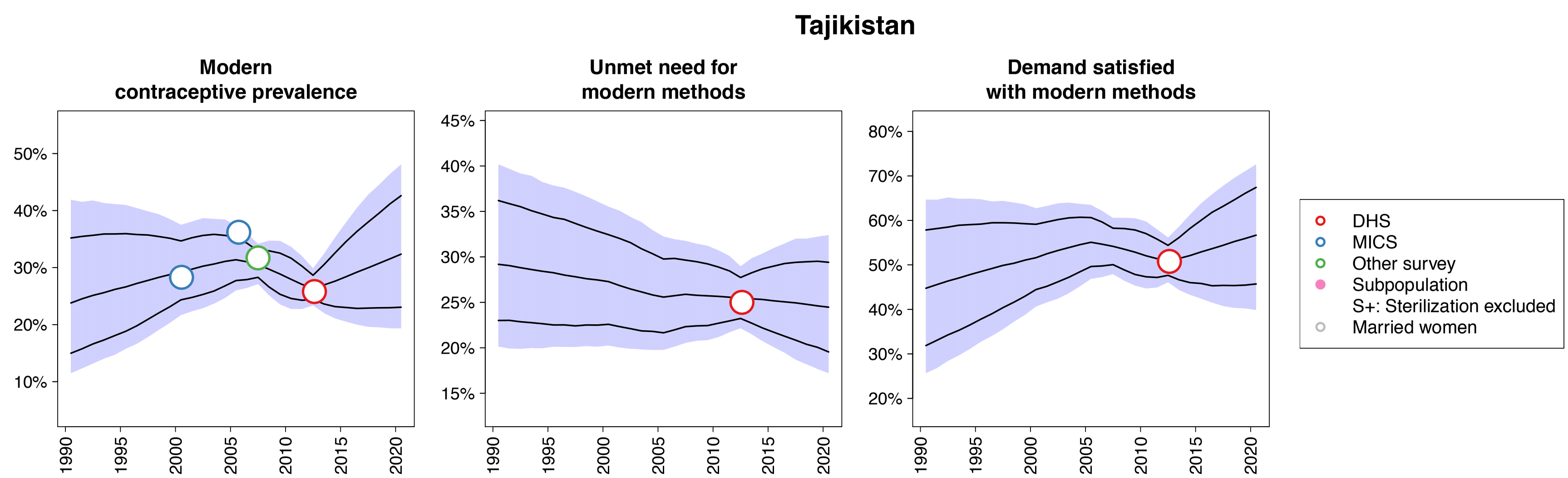
Tajikistan
Modern contraceptive prevalence
Unmet need for modern methods
Demand satisfied with modern methods
50%
40%
30%
20%
10%
45%
35%
25%
15%
80%
70%
60%
1990
1995
2000
2005
2010
2015
2020
DHS
MICS
Other survey
Subpopulation
S+: Sterilization excluded
Married women

Timor–Leste

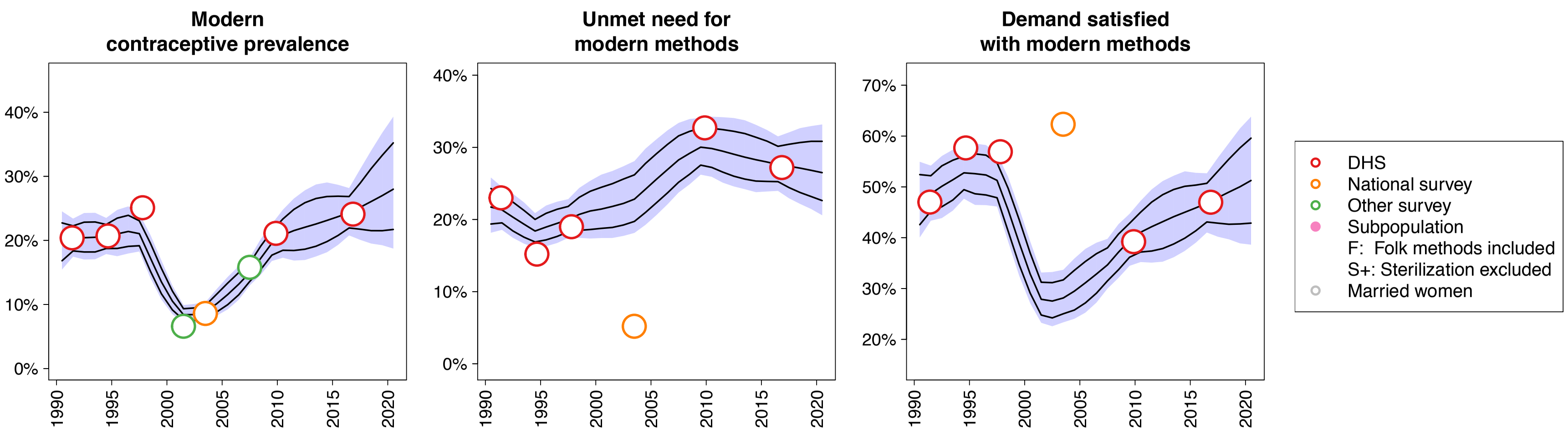
Modern
contraceptive prevalence
Unmet need for
modern methods
Demand satisfied
with modern methods
0%
10%
20%
30%
40%
50%
60%
70%
1990
1995
2000
2005
2010
2015
2020
DHS
National survey
Other survey
Subpopulation
F: Folk methods included
S+: Sterilization excluded
Married women

Togo

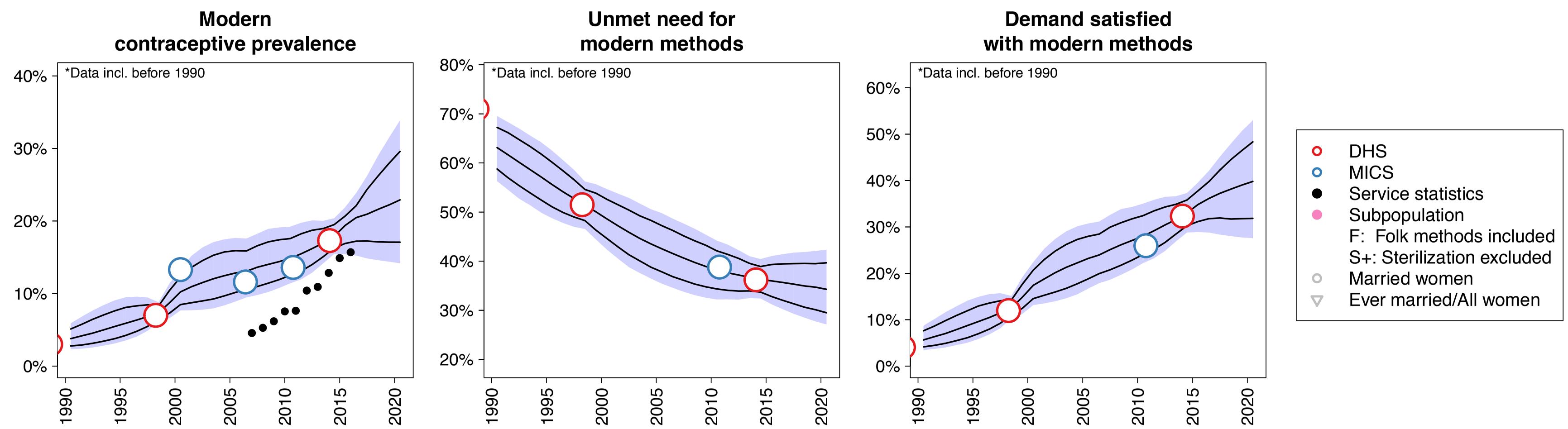
Modern
contraceptive prevalence
*Data incl. before 1990
40%
30%
20%
10%
0%
1990
1995
2000
2005
2010
2015
2020
Unmet need for
modern methods
*Data incl. before 1990
80%
70%
60%
50%
40%
30%
20%
Demand satisfied
with modern methods
*Data incl. before 1990
60%
50%
40%
30%
20%
10%
DHS
MICS
Service statistics
Subpopulation
F: Folk methods included
S+: Sterilization excluded
Married women
Ever married/All women

Uganda

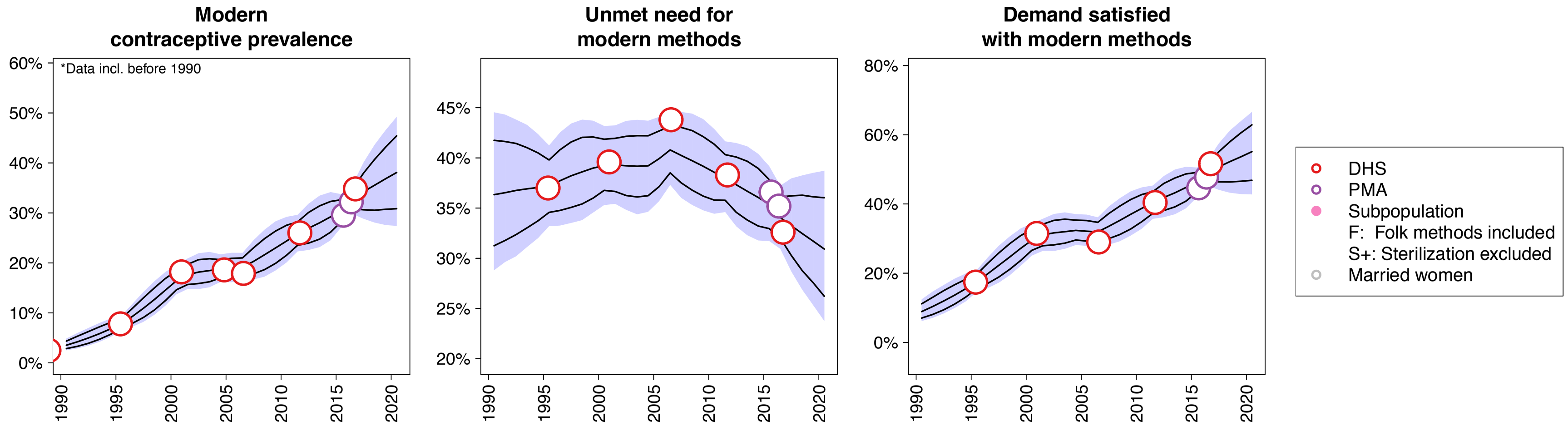
Modern
contraceptive prevalence
*Data incl. before 1990
60%
50%
40%
30%
20%
10%
0%
1990
1995
2000
2005
2010
2015
2020
Unmet need for
modern methods
45%
40%
35%
30%
25%
20%
Demand satisfied
with modern methods
80%
60%
40%
20%
0%
DHS
PMA
Subpopulation
F: Folk methods included
S+: Sterilization excluded
Married women

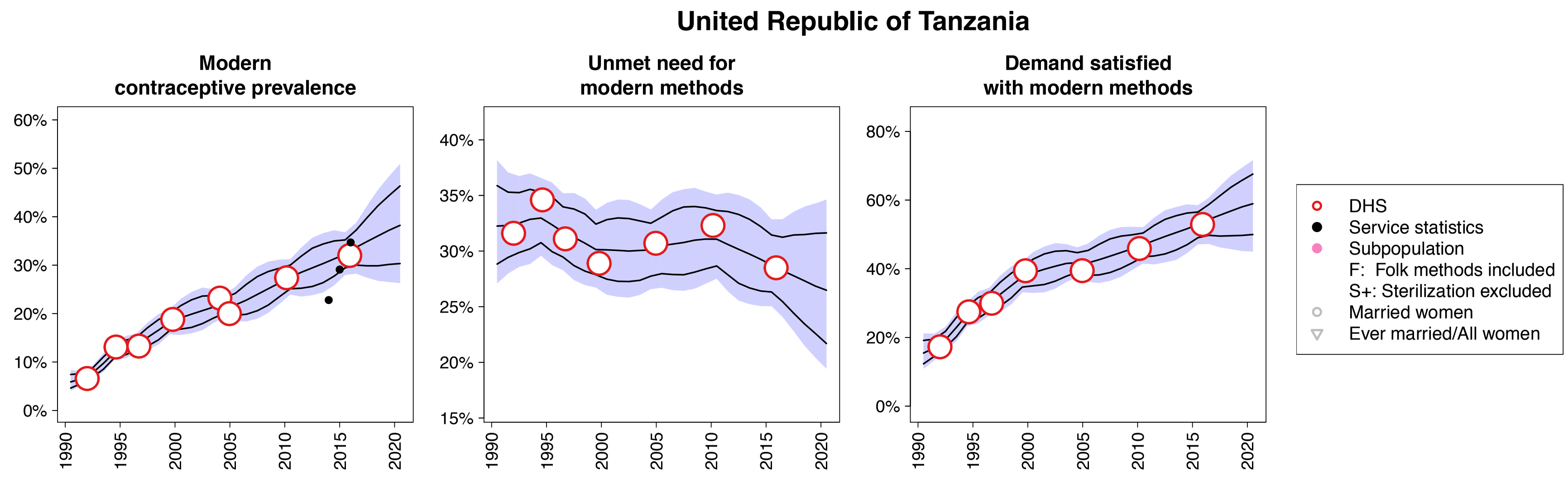
United Republic of Tanzania
Modern
contraceptive prevalence
Unmet need for
modern methods
Demand satisfied
with modern methods
DHS
Service statistics
Subpopulation
F: Folk methods included
S+: Sterilization excluded
Married women
Ever married/All women

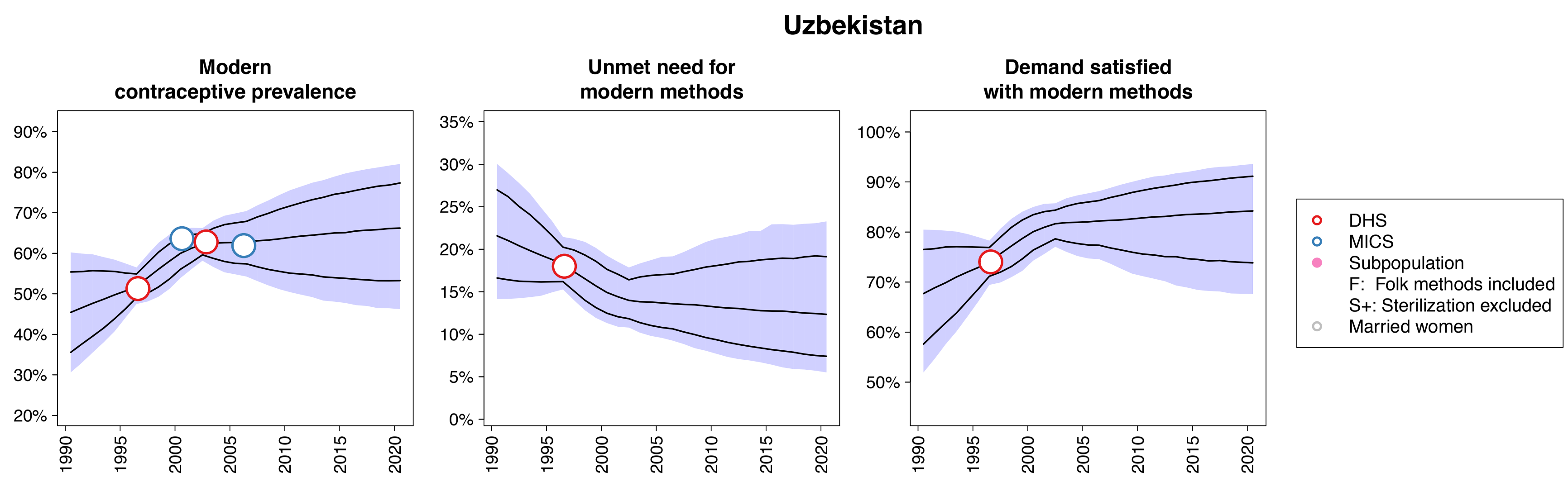

Uzbekistan
Modern contraceptive prevalence
Unmet need for modern methods
Demand satisfied with modern methods
90%
80%
70%
60%
50%
40%
30%
20%
35%
25%
15%
10%
5%
0%
100%
1990
1995
2000
2005
2010
2015
2020
DHS
MICS
Subpopulation
F: Folk methods included
S+: Sterilization excluded
Married women

Viet Nam

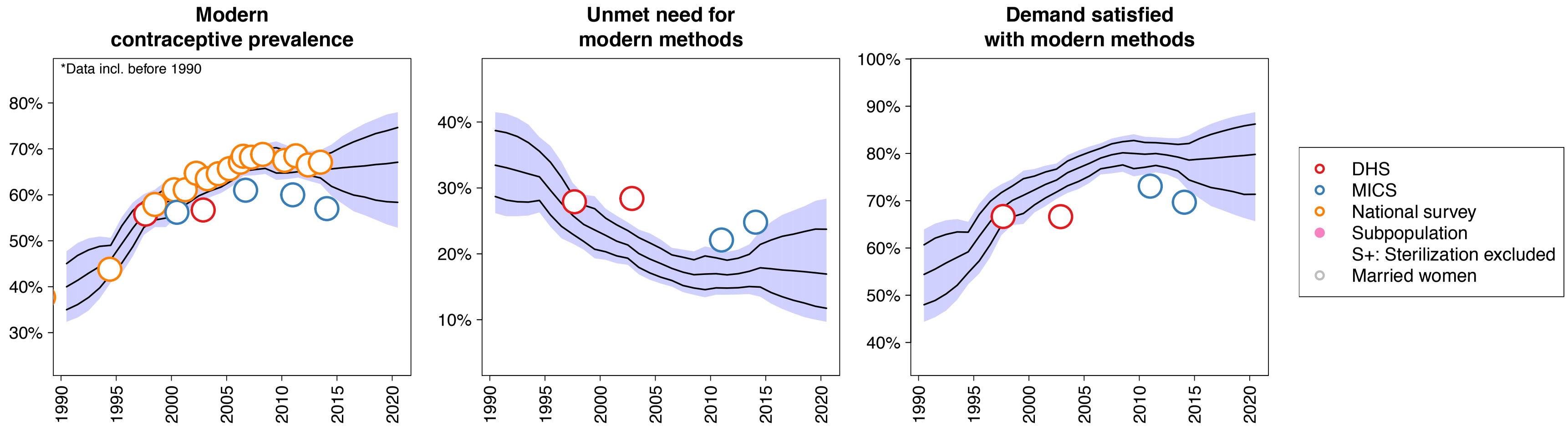

Modern contraceptive prevalence
*Data incl. before 1990
80%
70%
60%
50%
40%
30%
Unmet need for modern methods
40%
30%
20%
10%
Demand satisfied with modern methods
100%
90%
80%
70%
60%
50%
40%
1990
1995
2000
2005
2010
2015
2020
DHS
MICS
National survey
Subpopulation
S+: Sterilization excluded
Married women

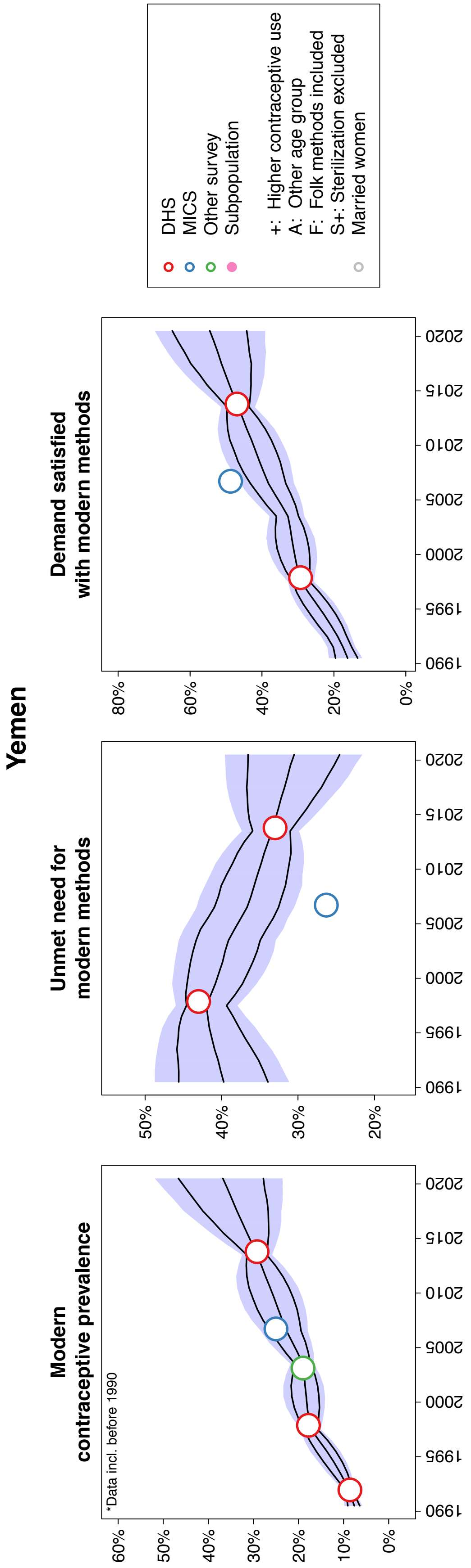
Yemen
Modern contraceptive prevalence
*Data incl. before 1990
Unmet need for modern methods
Demand satisfied with modern methods
DHS
MICS
Other survey
Subpopulation
+: Higher contraceptive use
A: Other age group
F: Folk methods included
S+: Sterilization excluded
Married women

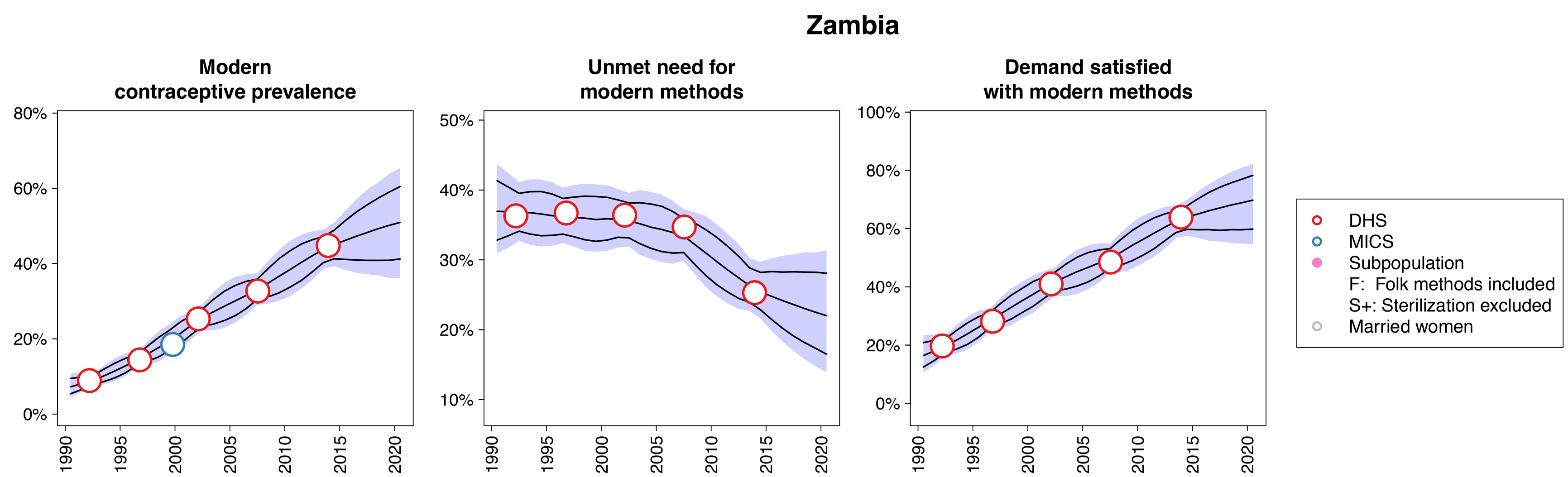
Zambia
Modern
contraceptive prevalence
80%
60%
40%
20%
0%
1990
1995
2000
2005
2010
2015
2020
Unmet need for
modern methods
50%
40%
30%
20%
10%
Demand satisfied
with modern methods
100%
DHS
MICS
Subpopulation
F: Folk methods included
S+: Sterilization excluded
Married women

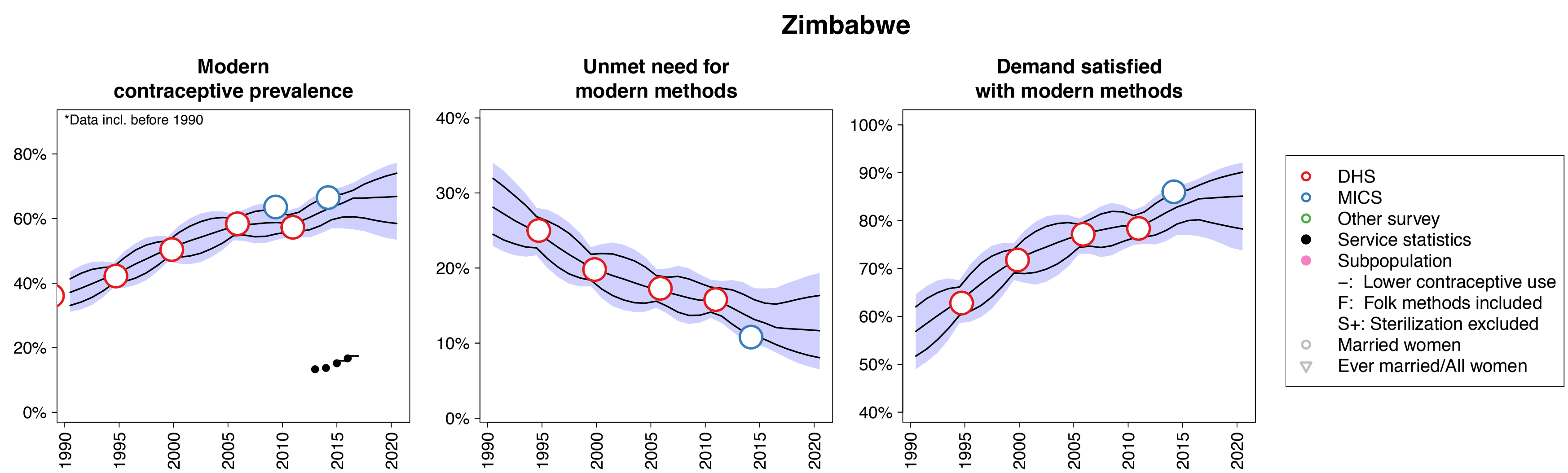
Zimbabwe
Modern
contraceptive prevalence
*Data incl. before 1990
0%
20%
40%
60%
80%
1990
1995
2000
2005
2010
2015
2020
Unmet need for
modern methods
0%
10%
20%
30%
40%
Demand satisfied
with modern methods
40%
50%
60%
70%
80%
90%
100%
DHS
MICS
Other survey
Service statistics
Subpopulation
−: Lower contraceptive use
F: Folk methods included
S+: Sterilization excluded
Married women
Ever married/All women

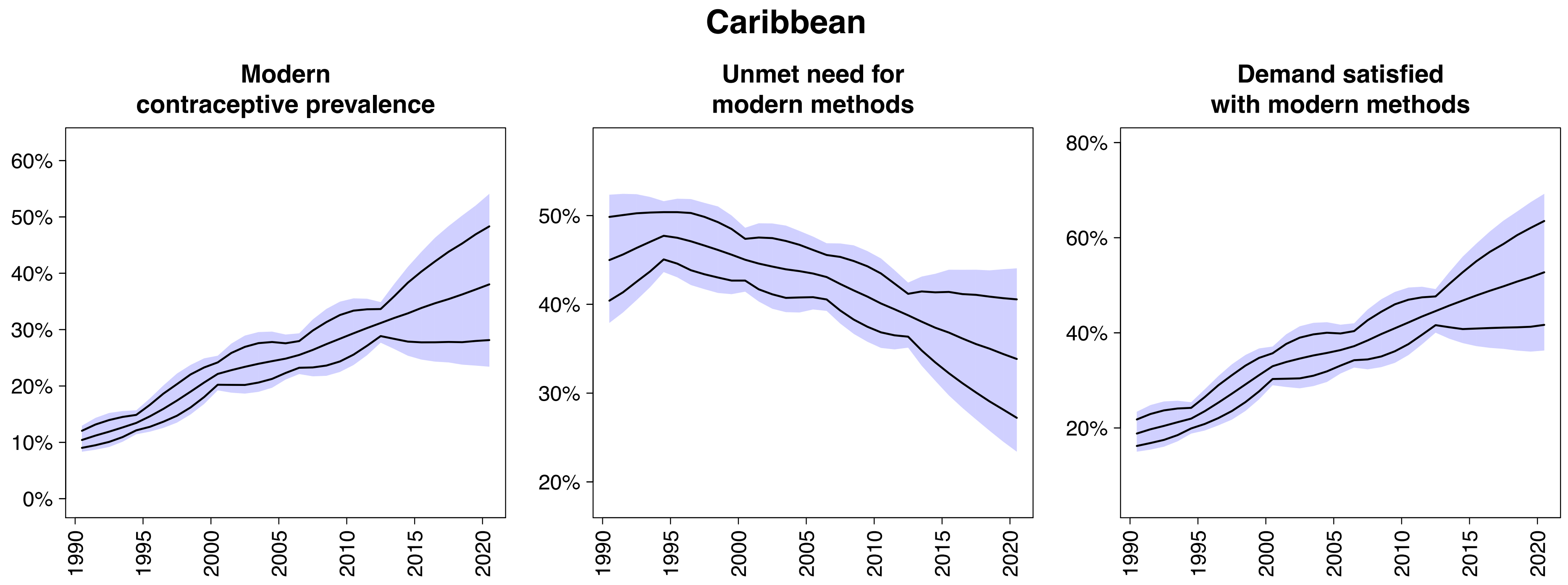
Caribbean
Modern contraceptive prevalence
Unmet need for modern methods
Demand satisfied with modern methods
60%
50%
40%
30%
20%
10%
0%
80%
1990
1995
2000
2005
2010
2015
2020

Central America

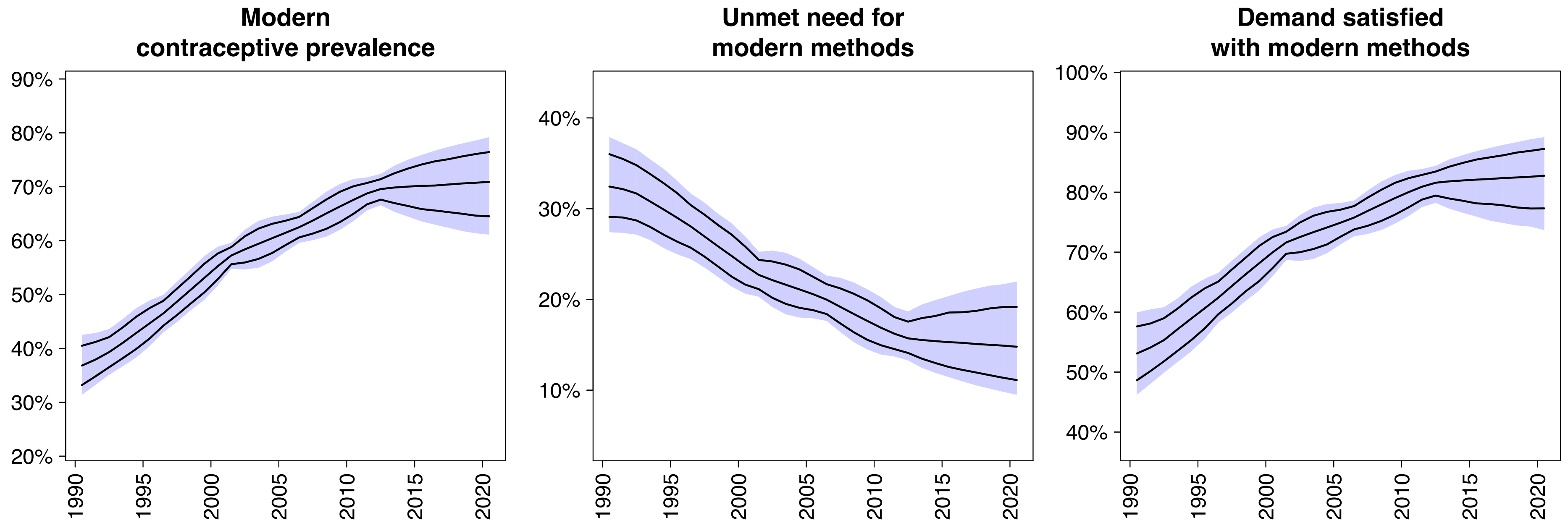

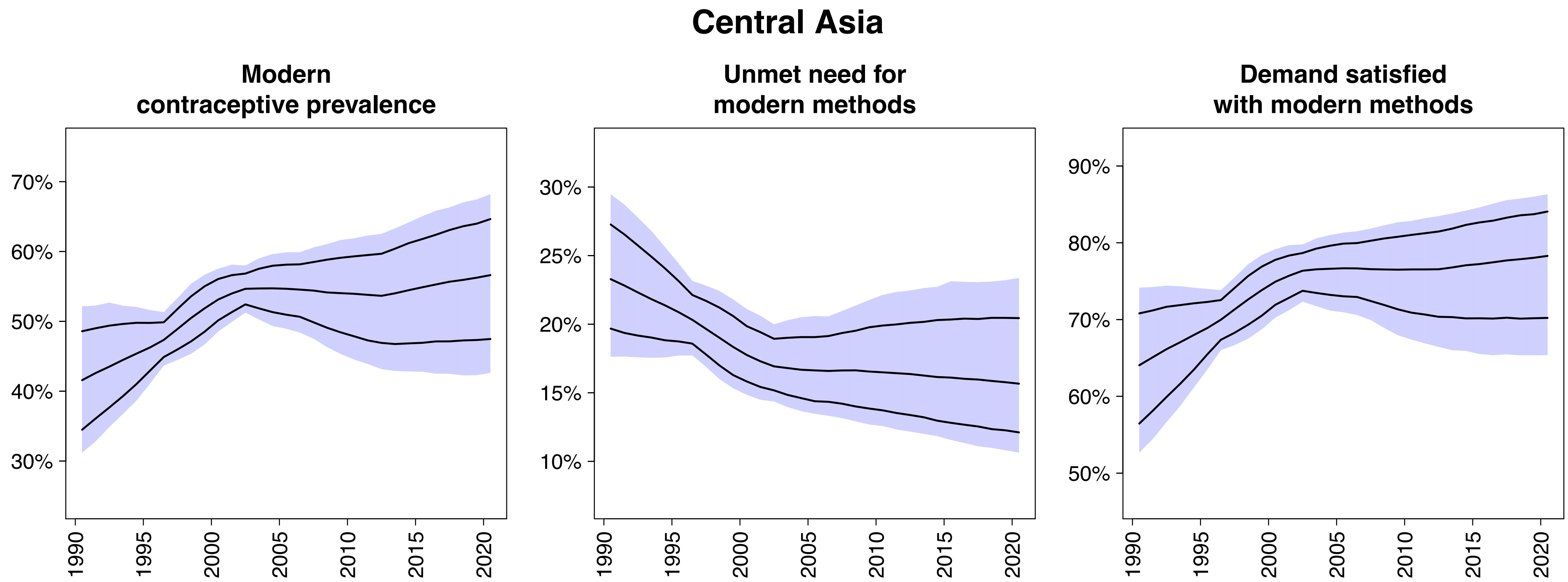
Central Asia
Modern
contraceptive prevalence
Unmet need for
modern methods
Demand satisfied
with modern methods
70%
60%
50%
40%
30%
25%
20%
15%
10%
90%
80%
1990
1995
2000
2005
2010
2015
2020

Eastern Africa

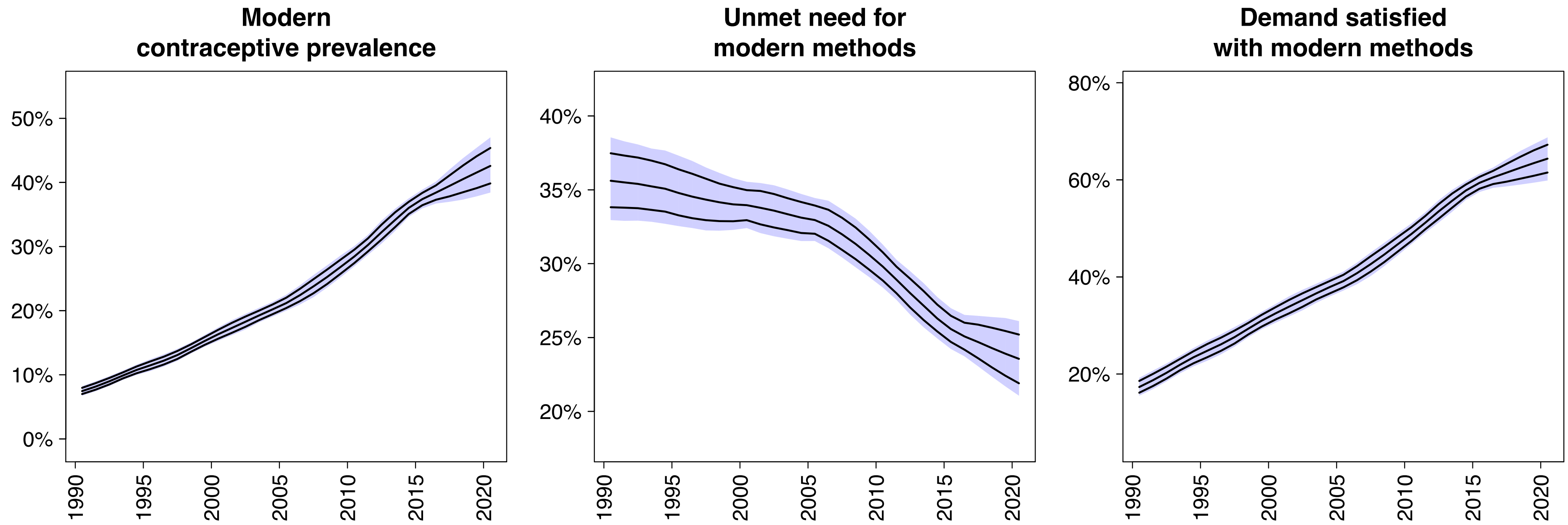

Eastern Asia

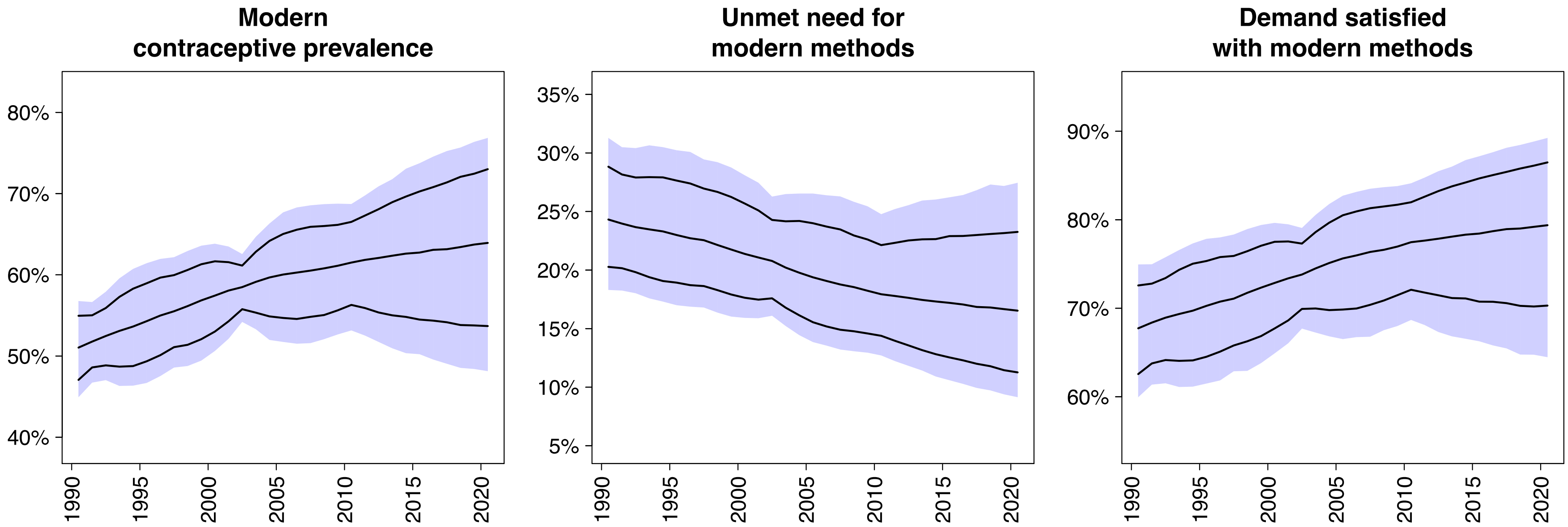

Melanesia

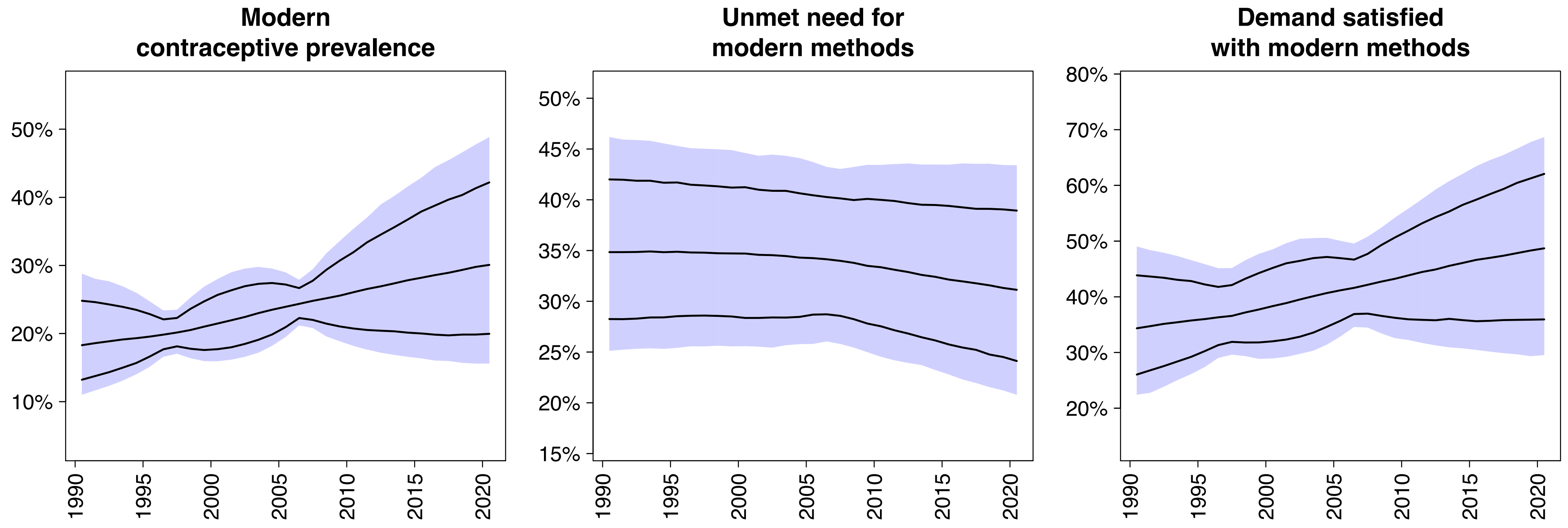
Modern contraceptive prevalence
50%
40%
30%
20%
10%
1990
1995
2000
2005
2010
2015
2020
Unmet need for modern methods
50%
45%
40%
35%
30%
25%
20%
15%
Demand satisfied with modern methods
80%
70%
60%
50%
40%
30%
20%

Middle Africa

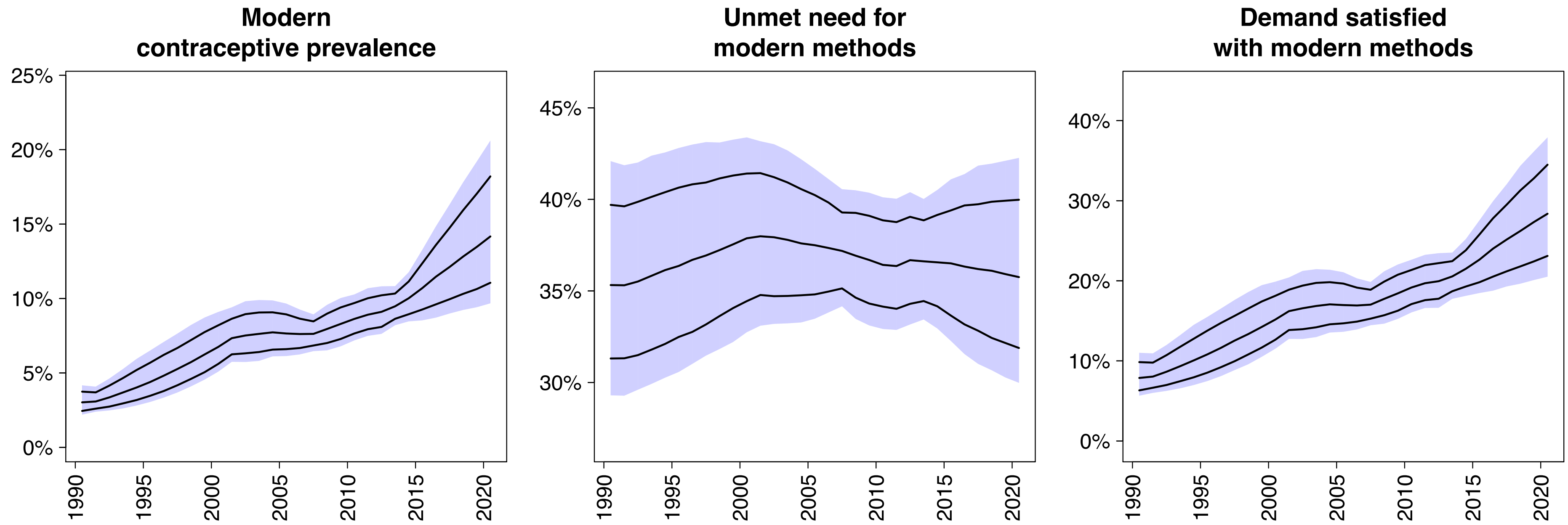

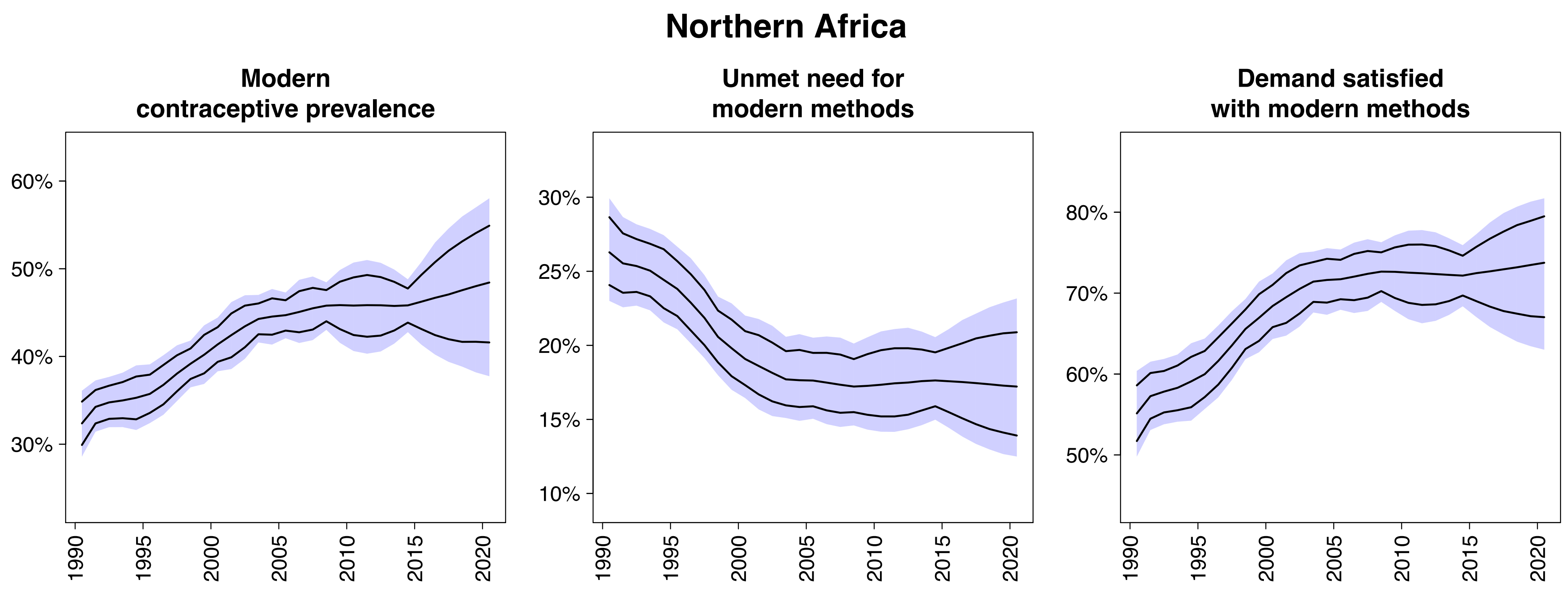
Northern Africa
Modern
contraceptive prevalence
60%
50%
40%
30%
Unmet need for
modern methods
30%
25%
20%
15%
10%
Demand satisfied
with modern methods
80%
70%
60%
50%
1990
1995
2000
2005
2010
2015
2020

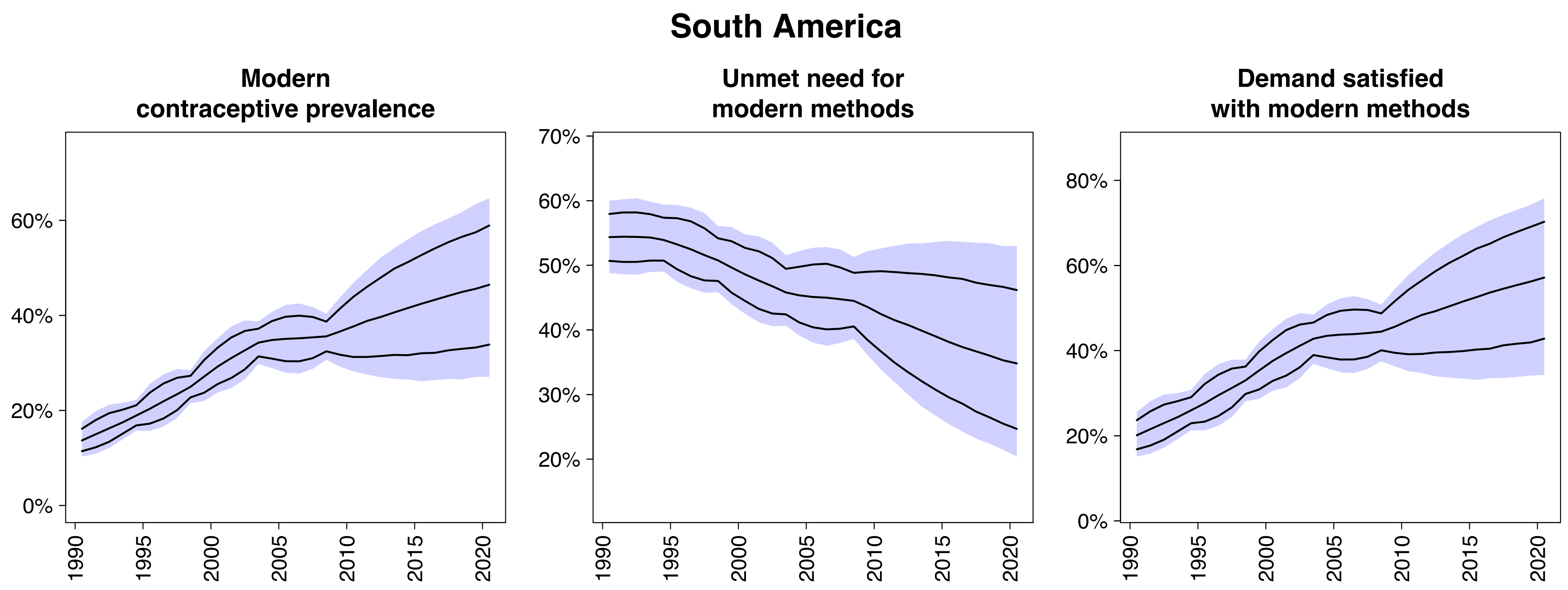
South America
Modern
contraceptive prevalence
Unmet need for
modern methods
Demand satisfied
with modern methods
0%
20%
30%
40%
50%
60%
70%
80%
1990
1995
2000
2005
2010
2015
2020

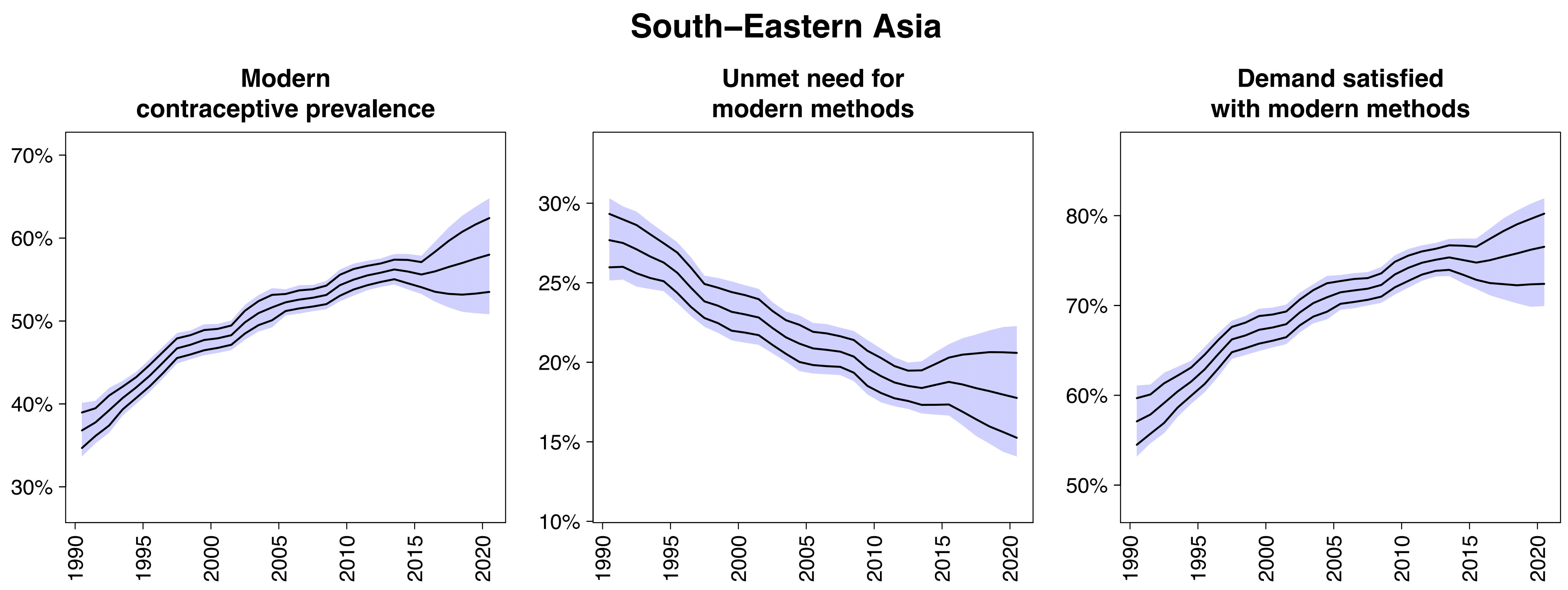
South–Eastern Asia
Modern
contraceptive prevalence
70%
60%
50%
40%
30%
1990
1995
2000
2005
2010
2015
2020
Unmet need for
modern methods
30%
25%
20%
15%
10%
Demand satisfied
with modern methods
80%
70%
60%
50%

Southern Africa

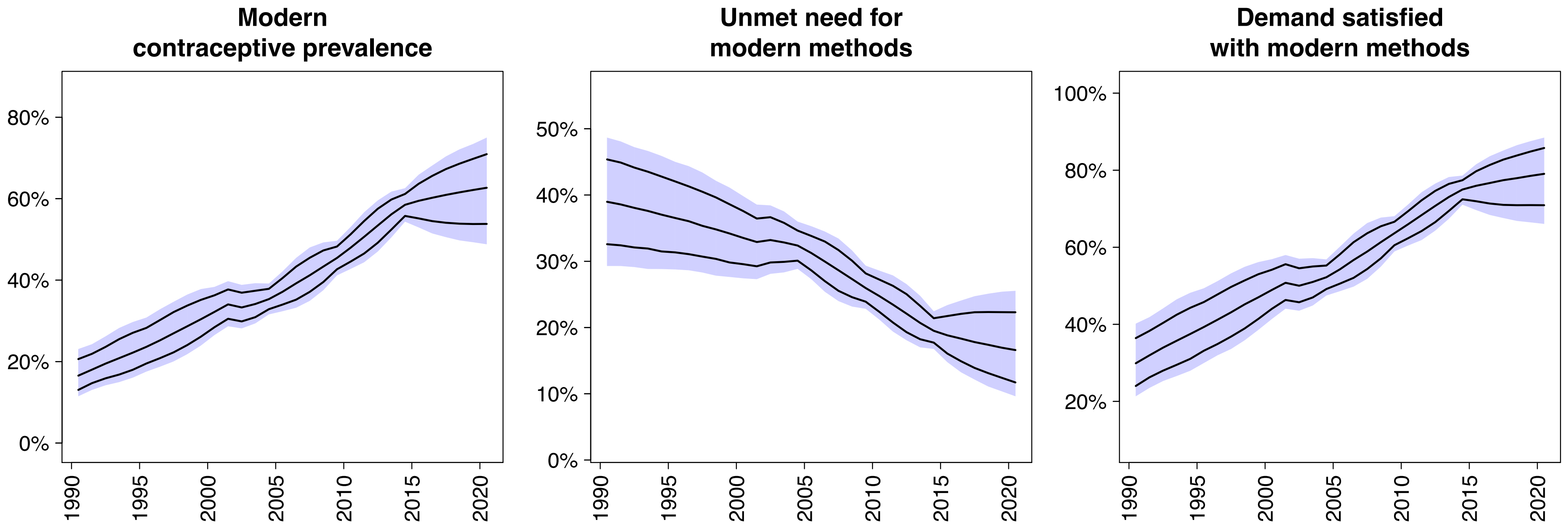

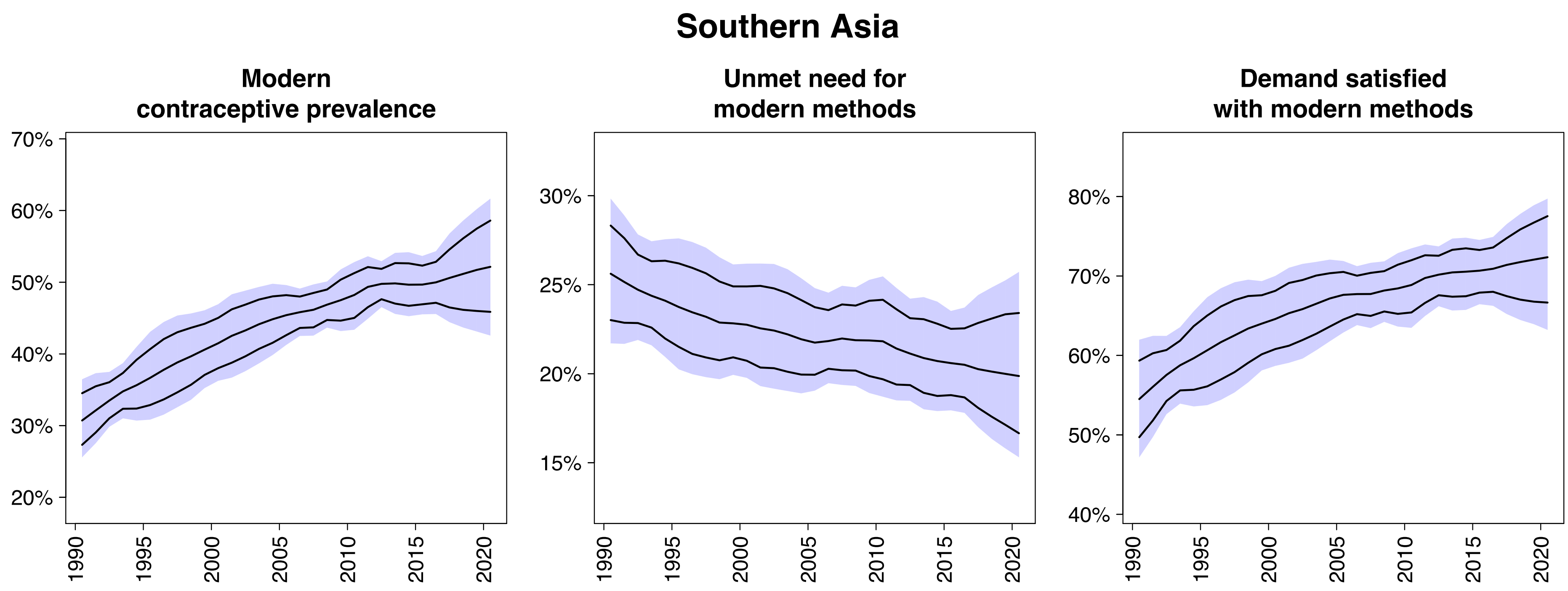
Southern Asia
Modern
contraceptive prevalence
70%
60%
50%
40%
30%
20%
Unmet need for
modern methods
30%
25%
20%
15%
Demand satisfied
with modern methods
80%
70%
60%
50%
40%
1990
1995
2000
2005
2010
2015
2020

Western Africa

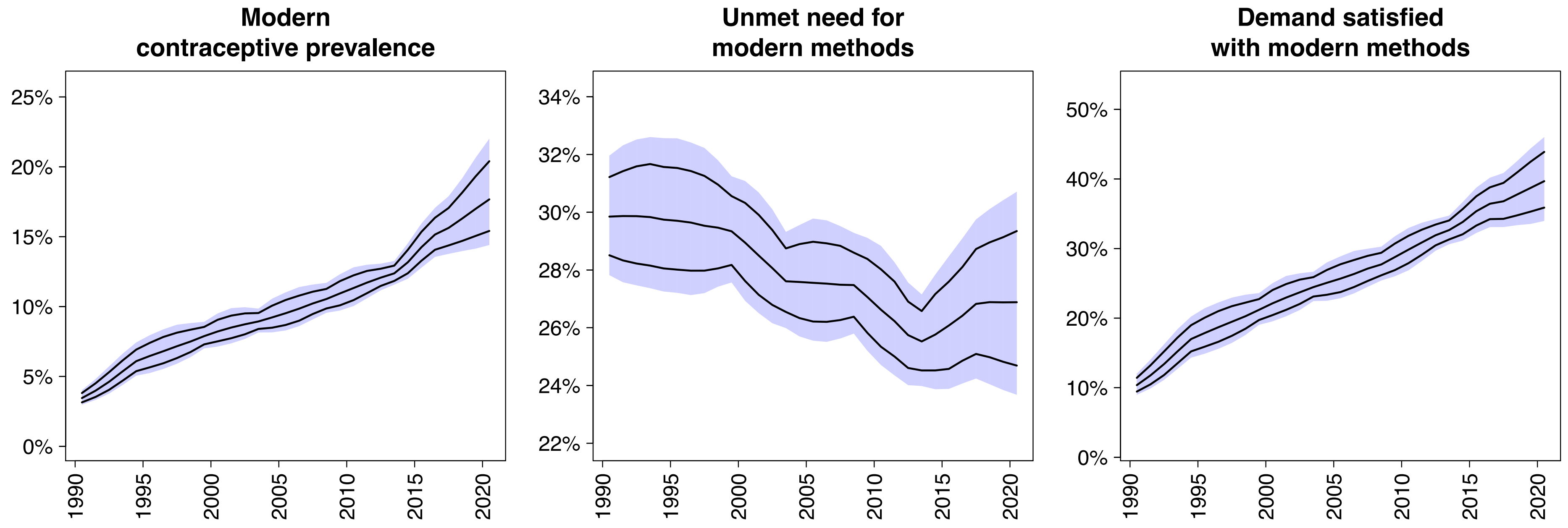

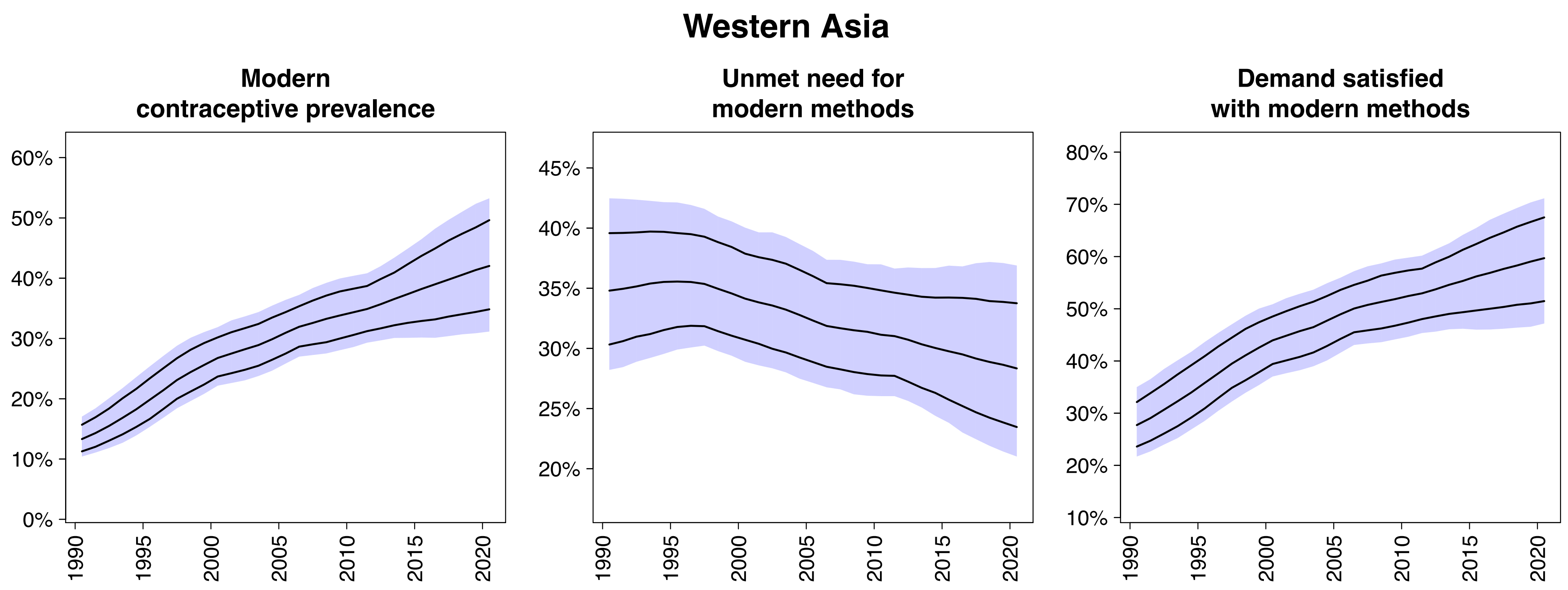

Western Asia
Modern
contraceptive prevalence
60%
50%
40%
30%
20%
10%
0%
1990
1995
2000
2005
2010
2015
2020
Unmet need for
modern methods
45%
40%
35%
30%
25%
20%
Demand satisfied
with modern methods
80%
70%
60%
50%
40%
30%
20%
10%

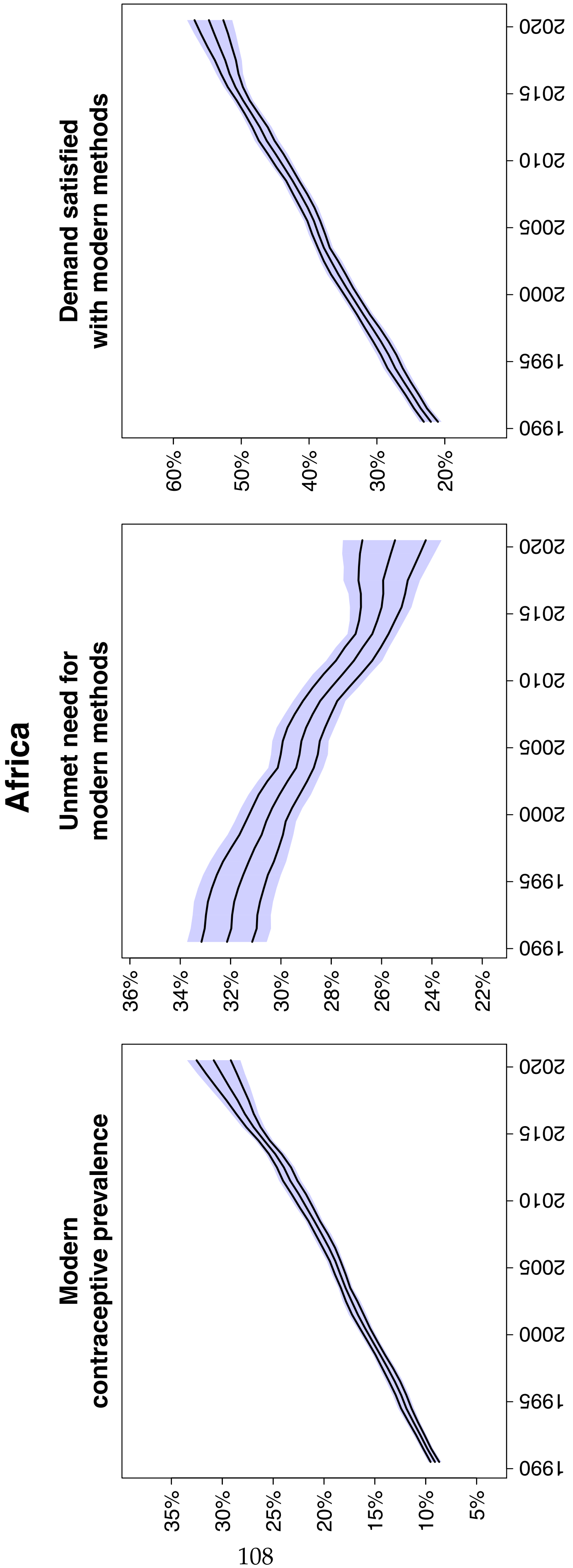
Africa
Modern contraceptive prevalence
35%
30%
25%
20%
15%
10%
5%
Unmet need for modern methods
36%
34%
32%
30%
28%
26%
24%
22%
Demand satisfied with modern methods
60%
50%
40%
30%
20%
1990
1995
2000
2005
2010
2015
2020

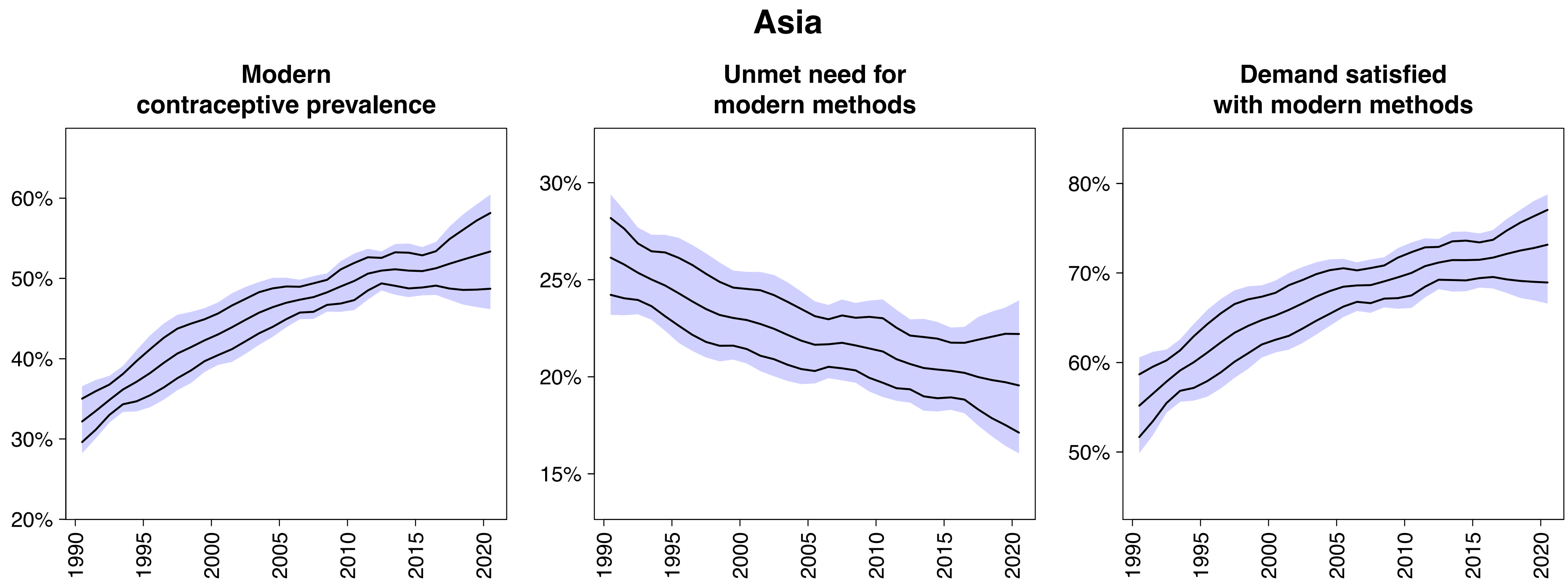
Asia
Modern
contraceptive prevalence
Unmet need for
modern methods
Demand satisfied
with modern methods
60%
50%
40%
30%
20%
30%
25%
20%
15%
80%
70%
60%
50%
1990
1995
2000
2005
2010
2015
2020

Latin America and the Caribbean

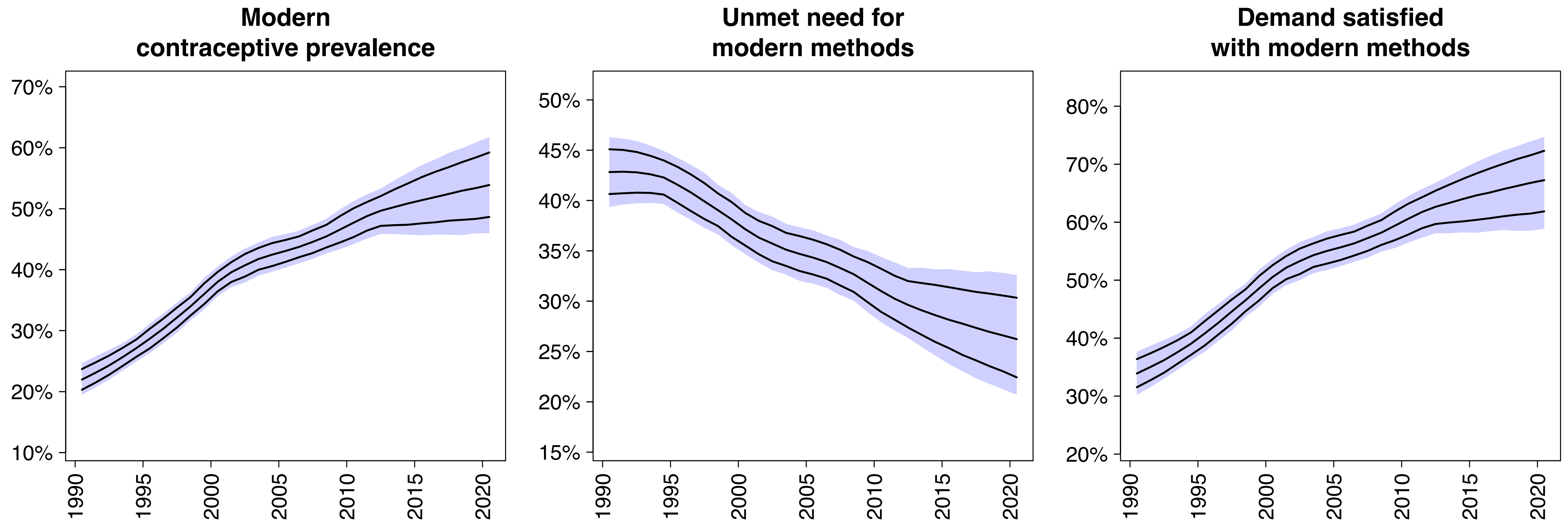

Oceania

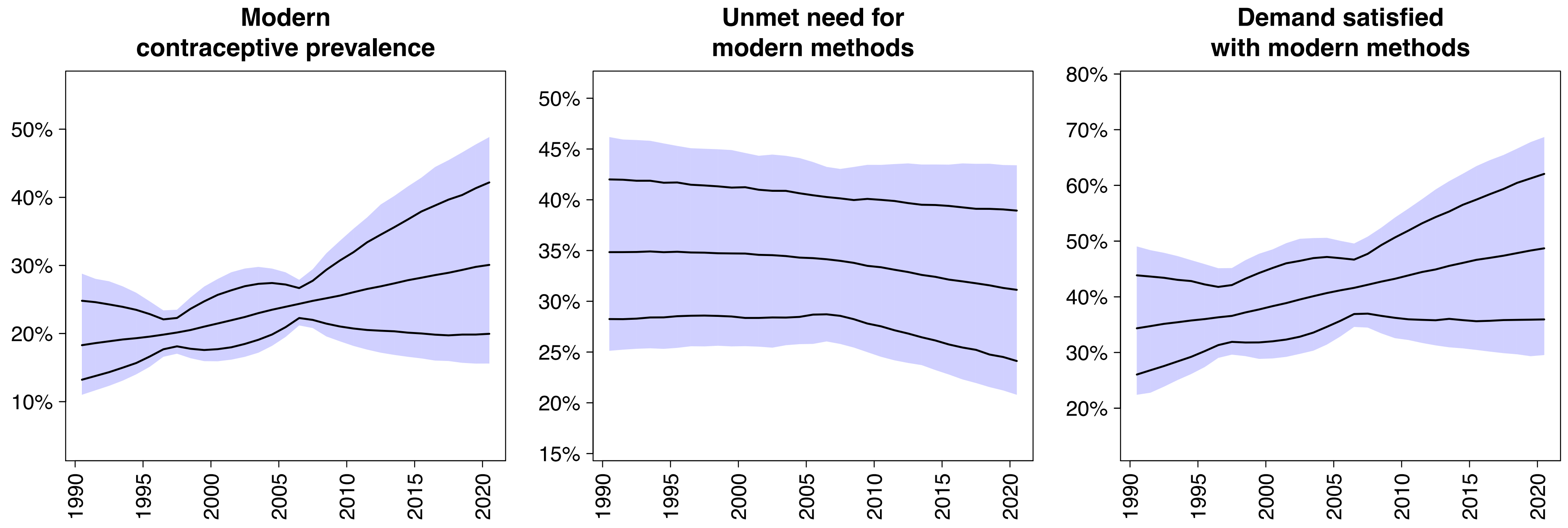
Modern
contraceptive prevalence
Unmet need for
modern methods
Demand satisfied
with modern methods
50%
40%
30%
20%
10%
45%
35%
25%
15%
80%
70%
60%
1990
1995
2000
2005
2010
2015
2020

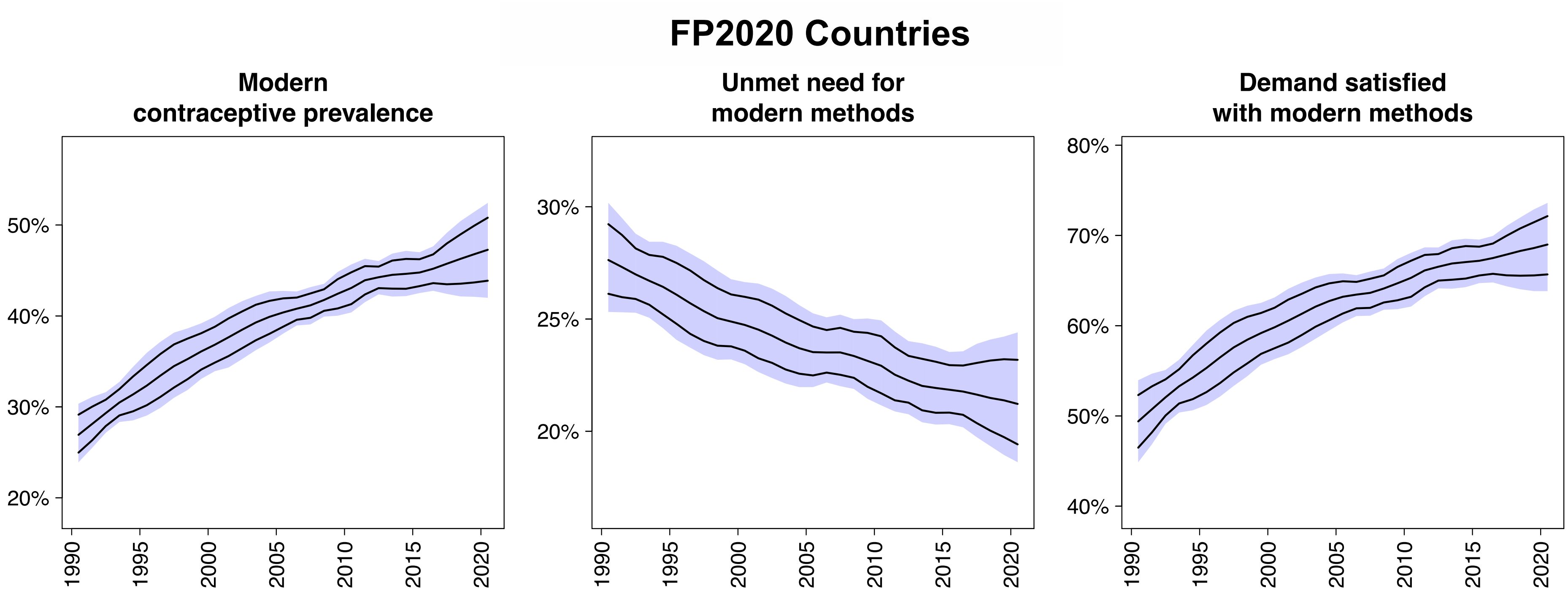
FP2020 Countries
Modern
contraceptive prevalence
Unmet need for
modern methods
Demand satisfied
with modern methods
20%
30%
40%
50%
25%
80%
70%
60%
1990
1995
2000
2005
2010
2015
2020
